# ﻿A taxonomic revision of *Cynanchumthesioides* (Apocynaceae) with two new synonyms

**DOI:** 10.3897/phytokeys.219.93514

**Published:** 2023-01-19

**Authors:** Cai-Fei Zhang, Dong-Juan Zhang, Miao Liao, Guang-Wan Hu

**Affiliations:** 1 CAS Key Laboratory of Plant Germplasm Enhancement and Specialty Agriculture, Wuhan Botanical Garden, Chinese Academy of Sciences, Wuhan 430074, China Wuhan Botanical Garden, Chinese Academy of Sciences Wuhan China; 2 Sino-Africa Joint Research Center, Chinese Academy of Sciences, Wuhan 430074, China Sino-Africa Joint Research Center, Chinese Academy of Sciences Wuhan China; 3 University of Chinese Academy of Sciences, Beijing 100049, China University of Chinese Academy of Sciences Beijing China

**Keywords:** Asclepiadeae, China, Mongolia, taxonomy, typification, *Vincetoxicum*

## Abstract

*Cynanchumthesioides*, a species widely distributed in north-eastern Asia, is revised to include two new synonyms: Vincetoxicumsibiricumf.linearifolium, described from Shandong, China in 1877, but long neglected and *Cynanchumgobicum*, previously believed to be endemic to Mongolia. Typification for *C.thesioides* and all its synonyms is given, including lectotypification of V.sibiricumvar.australe and V.sibiricumf.linearifolium. An updated description, three figures showing the diverse habitats, habits and variation in morphological characters, and a general distribution map are also provided.

## ﻿Introduction

*Cynanchum* L. is a large genus with more than 200 species worldwide (Endress et al. 2018). Recent morphological, chemical and molecular studies (Qiu et al. 1989; Liede 1996; Liede and Täuber 2002; Khanum et al. 2016) have significantly altered the circumscription of *Cynanchum*, resulting in the inclusion of several small genera and the transfer of several species to *Vincetoxicum* Wolf.

**Figure 1.**
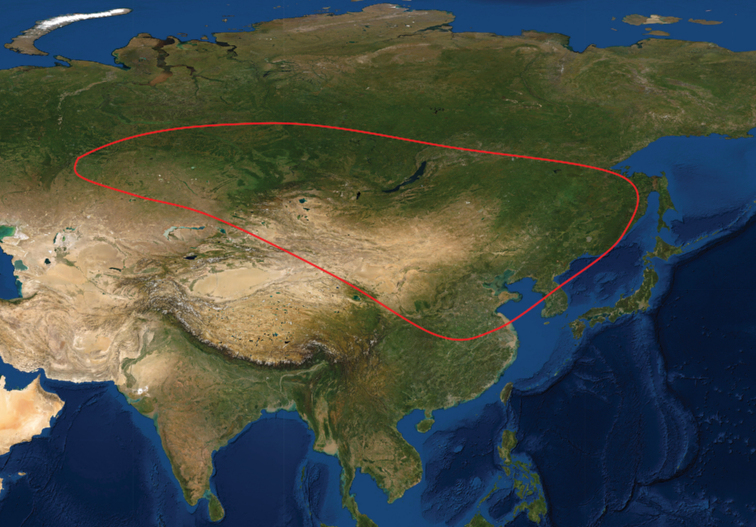
Distribution map of *Cynanchumthesioides* in north-eastern Asia. The base map was downloaded from the Microsoft Bing Satellite Map via QGIS 3.26 (https://qgis.org/). Map data 2022 (C) Microsoft.

One of the most widespread species in the genus, *Cynanchumthesioides* (Freyn) K. Schum., is found in temperate NE Asia, from eastern Kazakhstan, Mongolia, northern China or the Korean Peninsula (Fig. 1). It is distinguished by the erect/semi-erect stems, cuneate to rounded leaf bases and a 1-seriate corona, while most other species in *Cynanchum* display a climbing habit, cordate leaf bases and 2-seriate coronas (Khanum et al. 2016; Endress et al. 2018). As a result, some taxonomists placed it in *Vincetoxicum* (e.g. Freyn 1890; Pobedimova 1952; Kovtonyuk 1997). However, all species of the recently recircumscribed *Vincetoxicum* have clear, not white latex (Liede-Schumann et al. 2016; Endress et al. 2018), whereas the sap of *C.thesioides* is white. It had even been placed in its own genus, *Rhodostegiella* C.Y. Wu & D.Z. Li (Li et al. 1990), because of its distinctive chemical constituents. Recent molecular studies confirmed its position in *Cynanchum* (Khanum et al. 2016; Hu et al. 2020; Kang et al. 2021). It is a traditional medicinal and edible plant used by the Chinese (Tsiang and Li 1977); records of its usage can be traced back nearly one thousand years (Wu 2017: 778). Moreover, it provides good fodder resources for domestic animals (Huang and Liu 1992).

During taxonomic studies of *Cynanchum* from Central and East Asia, we found that the distinctions between *C.thesioides* and *C.gobicum* (Grubov) Grubov are questionable. The latter was considered to be endemic to Mongolia (Grubov 2000, 2002; Urgamal et al. 2014), or subendemic, meaning that it could be found in the neighbouring countries near their borders with Mongolia (Urgamal 2017). In fact, *C.gobicum* is very likely to be found in Nei Mongol (Inner Mongolia), China, since one of the localities listed by Grubov (2002: 109) lies about 5 kilometres from the border of Mongolia with China. In this study, we aim to confirm the identity of *C.gobicum* and provide an updated taxonomy of *C.thesioides*.

## ﻿Materials and methods

We examined more than 1600 specimens in 73 herbaria (ABGI, ANUB, AU, B, BJFC, BJTC, BM, BNU, BRNM, BRY, CDBI, CQNM, E, FSU, GXMG, GXMI, GZAC, HBNU, HEAC, HENU, HHBG, HIB, HIMC, HNWP, HSIB, IATM, IBK, IBSC, IFP, JJF, JLSLKY, K, KUN, KUZ, L, LBG, LE, LINN, M, MO, MW, NAS, NEAU, NEFI, NMAC, NMTC, NY, P, PE, PEY, PRC, QFNU, QYTC, RSA, S, SDFGR, SM, SVER, SXTCM, SXU, SYAU, SZG, TI, TIE, VBGI, WA, WAG, WH, WIS, WUK, XBGH, YAK and Z; herbarium abbreviations follow Thiers 2022 [continuously updated]). Fresh material was collected from the National Botanical Garden (South Garden), Beijing. Fresh flowers and those kept in FAA were examined under a stereomicroscope Nikon SMZ25. This enabled us to check the micromorphology of trichomes and floral characters. Digital images from online databases were checked: the Chinese Virtual Herbarium (CVH; https://www.cvh.ac.cn/), the Integrated Digitized Biocollections (iDigBio; https://www.idigbio.org/), the Global Biodiversity Information Facility (GBIF; https://www.gbif.org/), the JACQ specimen database (https://www.jacq.org/), the JSTOR Global Plants database (https://plants.jstor.org/), the BioPortal Naturalis collections (https://bioportal.naturalis.nl/) and the online herbarium catalogues of LE, P and S were examined. More than 2000 photos from the Plant Photo Bank of China (PPBC; http://ppbc.iplant.cn/sp/27237 [accessed in October 2022]) were also checked. A full list of specimens and selected observations examined is given in Suppl. material 1.

## ﻿Taxonomy

### ﻿ Cynanchum thesioides

Taxon classificationPlantae

(Freyn) K. Schum. in Engl. & Prantl, Nat. Pflanzenfam. 4 (2): 252 (1895).

B721F74A-D7A7-5F8C-8B97-44BE49567287

**Figure 2.**
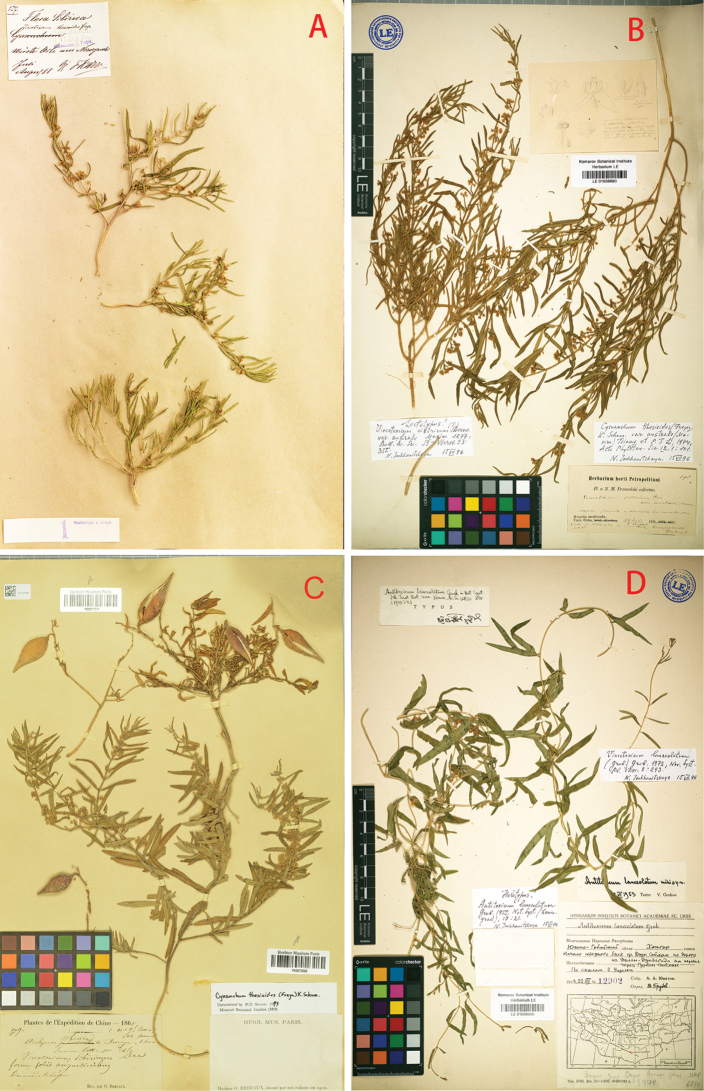
Type specimens **A** holotype of *Cynanchumthesioides* (BRNM [15481/36]]) **B** lectotype of Vincetoxicumsibiricumvar.australe (LE [LE01036690]) **C** lectotype of *Vincetoxicumsibiricum*. f.linearifolium (flowering plants, P [P03872669]), with fruiting plants (syntype, P [P00877371]) mounted on the same sheet: **D** holotype of *Antitoxicumlanceolatum* (LE [LE01036905]).

≡ Vincetoxicumthesioides Freyn, Oesterr. Bot. Z. 40: 124 (1890) ≡ Cynanchumsibiricumvar.thesioides (Freyn) Kom., Trudy Imp. S.-Peterburgsk. Bot. Sada 25(1): 282 (1905) — Holotype: Russia, wüste Orte um Nerczynsk, July-Aug. 1888, *K.F. Karo 127* (BRNM [15481/36], ex herb. J. Freyn) — Fig. 2A.
= Asclepiassibirica L., Sp. Pl.: 217 (1753) ≡ Cynanchumsibiricum (L.) R. Br., Mem. Wern. Nat. Hist. Soc. 1: 48 (1811), nom. illeg., non Willd. (1799). ≡ Vincetoxicumsibiricum (L.) Decne. in DC, Prodr. 8: 525 (1844) ≡ Vincetoxicumsibiricumvar.boreale Maxim., Bull. Acad. Imp. Sci. Saint-Pétersbourg 23: 355 (Mar 1877), nom. illeg., as ‘*borealis*’, ≡ Cynanchumsibiricumvar.boreale (Maxim.) Kom., Trudy Imp. S.-Peterburg sk. Bot. Sada 25(1): 281 (1905), nom. illeg. ≡ Antitoxicumsibiricum (L.) Pobed., Fl. USSR 18: 707–708, pl. 38: 1 (1952) ≡ Alexitoxiconsibiricum (L.) Pobed., Taxon 11: 174 (1962) ≡ Rhodostegiellasibirica (L.) C.Y. Wu & D.Z. Li, Acta Phytotax. Sin. 28(6): 466 (1990) — Lectotype (designated by Grubov [2000: 138]): Russia, Siberia, *Gmelin s.n.*, Herb. Linn. No. 310.35 (LINN; image available at https://linnean-online.org/2155/ and https://plants.jstor.org/stable/10.5555/al.ap.specimen.linn-hl310-35).
= Vincetoxicumsibiricumvar.australe Maxim., Bull. Acad. Imp. Sci. Saint-Pétersbourg 23: 355 (Mar. 1877), “*australem*” ≡ CynanchumsibiricumR. Br.var.australe (Maxim.) Kom., Trudy Imp. S.-Peterburgsk. Bot. Sada 25(1): 292 (1905). ≡ Cynanchumthesioidesvar.australe (Maxim.) Y. Tsiang & P.T. Li, Acta Phytotax. Sin. 12: 101 (1974) ≡ Rhodostegiellasibiricavar.australis (Maxim.) C.Y. Wu & D.Z. Li, Acta Phytotax. Sin. 28: 466 (1990) — Lectotype (designated here): China. Nei Mongol, “Mongolia occidentalis, Terra Ordos, valle fl. Hoang-ho”, 24 Jul – 5 Aug 1871 (fl.), *N.M. Przewalsky 298* (LE [LE01036690]); isolectotypes: K [000872724], LE [LE01036688, LE01036689], P [P03872677] — Fig. 2B.
= Vincetoxicumsibiricumf.linearifolium Debeaux, Actes Soc. Linn. Bordeaux 31(4): 235 (1877), “*linearifolia*”, syn. nov. — Lectotype (designated here): China, Shandong Prov., Yantai City, “Tchéfou dunes”, [1860], *O. Debeaux 79* (P [P03872669], ex herb. O. Debeaux) — Fig. 2C.
= Cynanchumsibiricumvar.gracilentum Nakai & Kitag., Rep. First Sci. Exped. Manch. sect. 4, 1: 43 (1934). ≡ Vincetoxicmsibiricumvar.gracilentum (Nakai & Kitag.) Kitag., Rep. Inst. Sci. Res. Manchoukuo 4(7): 85 (1940) — Holotype: China, Hebei Prov., Chengde, 19 Aug 1933 (fl.), *T. Nakai, M. Honda & M. Kitagawa s.n.* (TI [TI00204077]) — Suppl. material 2.
= Cynanchumsibiricumvar.gracilentumNakai & Kitag.f.hypopsilum Nakai & Kitag., Rep. First Sci. Exped. Manch., sect. 4, 1: 43 (1934) ≡ Vincetoxicmsibiricumf.hypopsilum (Nakai & Kitag.) Kitag., Rep. Inst. Sci. Res. Manchoukuo 4(7): 85 (1940) — Holotype: China, Hebei Prov., Chengde, 19 Aug 1933 (fl. & fr.), *T. Nakai, M. Honda & M. Kitagawa s.n.* (TI [TI00204078]) — Suppl. material 3.
= Cynanchumsibiricumvar.latifolium Kitag., Rep. First Sci. Exped. Manch. sect. 4, 4: 90, (1936) ≡ Cynanchumsibiricumvar.australef.latifolium (Kitag.) Kitag., Lineam. Fl. Mansh. 363 (1939) ≡ Vincetoxicmsibiricumf.latifolium (Kitag.) Kitag., Rep. Inst. Sci. Res. Manchoukuo 4(7): 85 (1940) — Holotype: China, Liaoning Prov., Dalian, Lingshui, Lingshui Temple, 15 Aug 1930 (fl.), *M. Kitagawa s.n.* (TI [TI00204080]) — Suppl. material 4.
= Cynanchumgobicum Grubov, Novosti Sist. Vyssh. Rast. 32: 135 (2000), non C.lanceolatum Poir. (1811), syn. nov. ≡ Antitoxicumlanceolatum Grubov, Bot. Mater. Gerb. Bot. Inst. Komarova Akad. Nauk S.S.S.R. (Leningrad) 17: 21 (1955) ≡ Vincetoxicumlanceolatum (Grubov) Grubov, Novosti Sist. Vyssh. Rast. 21: 208 (1984) — Holotype: Mongolia, Dzun-Saikhan mountains, commencement of northern trail along the road from Dalan-Dzadagad to pass through Gurban-Saikhan, 22 Jul 1943 (fl.), *A. Yunatov 12902* (LE [LE01036905]) — Fig. 2D.

#### Description.

**Figure 3.**
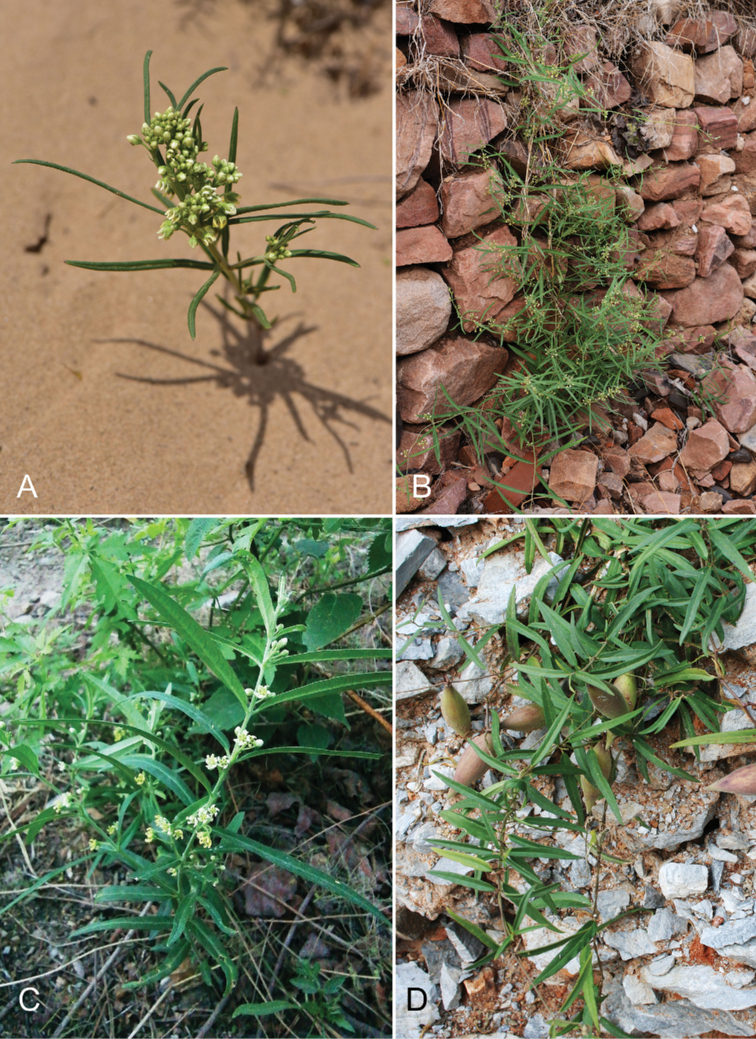
*Cynanchumthesioides***A** erect plant growing in desert **B** scandent plant climbing a stony wall **C** erect plant growing on mountain slope **D** prostrate plant on roadside wasteland **A** by Xin-Xin Zhu in Xilinhot, Nei Mongol **B, D** by Lei Xie in Beijing **C** from *C.F. Zhang 5588* (HIB) and photographed by Cai-Fei Zhang in Beijing.

**Figure 4.**
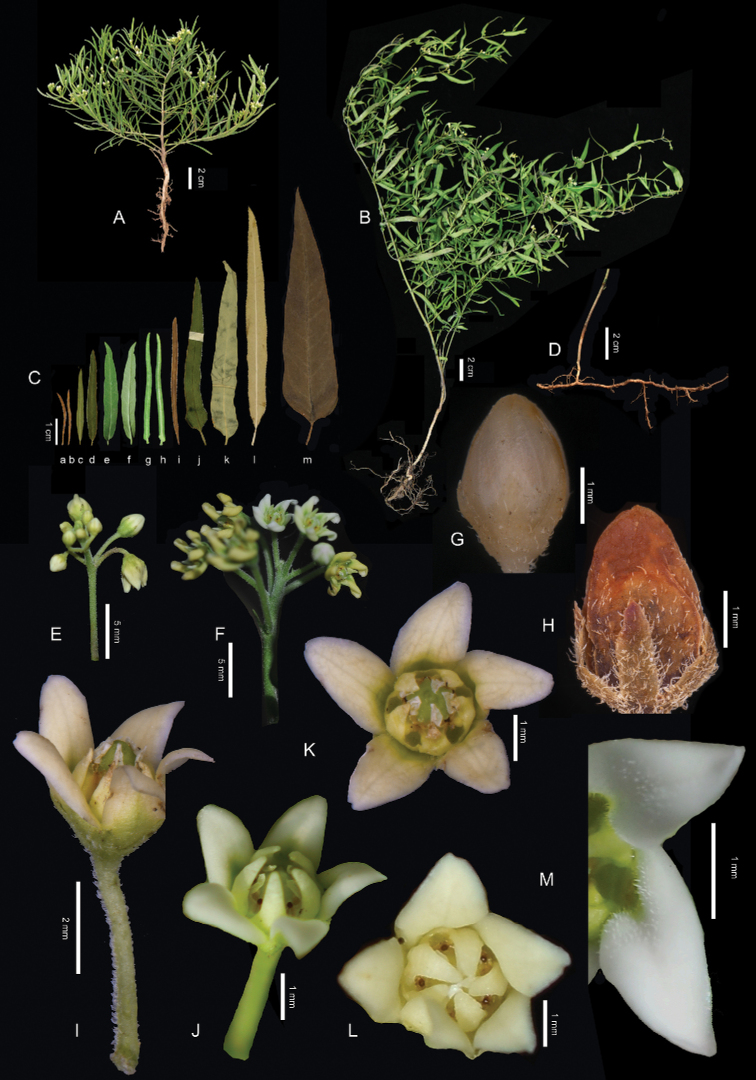
*Cynanchumthesioides***A** erect plant with part of horizontal rhizome **B** scandent plant with part of horizontal rhizome **C** leaves, **a, b, i** from the holotype of Cynanchumsibiricumvar.gracilentum (TI [00204077]) **c, d** from *I.A. Gubanov & Grubov 243* (MW [MW0187936]) which had been identified as *C.gobicum*; **j** from *I.A. Gubanov 3322* (MW [MW0187934]) which had been identified as *C.gobicum***k** from the holotype of *C.gobicum* (LE [LE01036905]) **l** from *G.M. Zhang et al. 070714* (BJFC [BJFC00062407]) **m** from the holotype of Cynanchumsibiricumvar.latifolium (TI [00204080]) **D** horizontal rhizome and root **E** inflorescence with buds **F** flowering inflorescence **G** bud with glabrous corolla **H** bud with puberulent corolla **I** flower with corona shorter than anther appendages (side view) **J** flower with corona longer than anther appendages (front view) **K** flower with corona shorter than anther appendages (vertical view) **L** flower with glabrous corolla and corona longer than anther appendages (vertical view) **M** part of flower showing two corolla lobes adaxially densely puberulent (vertical view) **B, C** (**e, f**), **E, G, I, K** and **L** from *Meng Wei in C.F. Zhang 6791* (HIB) **F** from *C.F. Zhang 5588***H** from *X.Y. Liu & F. Zhao 00283* (HIB [0101691]) **M** from *K.T. Fu 206* (HIB [0101693]) **A** by Shun-Bang Zhao in Xining; **B, C** (**e, f**), **E, I, K** and **L** by Miao Liao **C** (**g, h**) in Xilinhot and **J** in Beijing by Xin-Xin Zhu **D** by Jia-Hao Shen in Nanjing **F, G** and **H** by Cai-Fei Zhang **M** by Ye-Chun Xu in Beijing.

**Figure 5.**
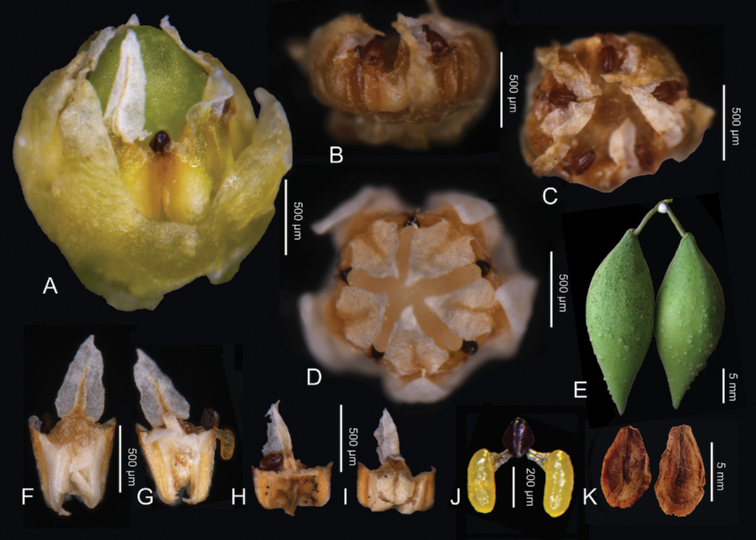
*Cynanchumthesioides***A** corona and gynostegium with long style-head (front view) **B** corona and gynostegium with short style-head (front view) **C** corona and gynostegium with short style-head (vertical view) **D** corona and gynostegium with long style-head (vertical view) **E** pair of fruits **F** stamen (front view) **G** stamen (back view) **H** stamen (front view) **I** stamen (back view) **J** pollinarium **K** seeds the concave surface (left) and convex surface (right) **A, D, F, G** and **J** from *Meng Wei in C.F. Zhang 6791* (HIB); **B, C, H, I** from *Zhongxiang Pubic Health Bureau s.n.* (HIB [0101695]). All photos by Cai-Fei Zhang, except **J** by Miao Liao, **E** by Bing Liu in Beijing and **K** by Qin-Wen Lin in Beijing.

Perennial suffrutescent sometimes lianescent herbs, usually densely shortly pubescent throughout, with white latex, arising from monopodial slightly woody creeping slender brown rhizome up to 3 m × 3 mm, with wiry horizontal offshoots. ***Stem and branches*** straight or twining above, green to dark purple; when straight 10–40 cm long, erect or ascending, simple or much divaricately branched from base, with internodes 2–30 mm long; when twining up to 2 m long, little branched, with internodes 4–8 cm long. ***Leaves*** opposite or sometimes subopposite, rarely 3- or 4-whorled, with petioles 0.5–10 (–12) mm long or subsessile; blade green, thin, linear, narrowly lanceolate, oblong-lanceolate or occasionally broadly lanceolate, 2–10.5 × 0.1–2 (–2.3) cm, apex shortly acute, obtuse or acuminate, rarely rounded, base slightly oblique, attenuate, cuneate, truncate or rounded, with colleters at middle of leaf base, margins entire, ciliolate, often revolute; mid-vein elevated abaxially, lateral veins obscure; both surfaces densely pubescent, sometimes glabrate, except the mid-veins on lower surface. ***Inflorescences*** alongside the leaf axils to terminal, with 1–10 fragrant flowers, shortly umbel- to raceme-like; ***peduncles*** 1–10 (–50) × 0.5–1.5 mm, puberulent; ***pedicels*** 1.2–10 × 0.2–0.6 mm, puberulent. ***Sepals*** ± half length of corolla, oblong, triangular or lanceolate, 1–2.8 × 0.3–0.8 mm, puberulent, ciliate, apex obtuse, acute or acuminate. ***Corolla*** white or greenish-white to yellow, 3–5 × 3–8 mm, usually glabrous, sometimes sparsely puberulent on dorsal surface and inside tube, rarely densely puberulent on both surfaces; ***tube*** 0.5–1.5 mm long; ***lobes*** 4.3–5.2 × 0.8–1.5 mm, narrowly triangular, lanceolate, oblong or oblong-ovate, apically twisted clockwise, apex ± acute or obtuse, sometimes retuse. ***Corona*** of 5 slightly fleshy lobes partly fused at bases, cupular, 0.8–1.8 mm long, shorter or longer than gynostegium; ***tube*** shorter than anthers, 0.3–0.7 mm long; ***lobes*** oval, triangular-lanceolate to linear, apices erect or incurved, acute, acuminate, obtuse or rounded, 0.5–1.4 mm long, sinuses between theses each with or without a minute triangular tooth. ***Follicles*** paired or single, ovoid-fusiform, ventricose, 4–10 × 0.8–2.5 cm, apex obtuse or attenuated, smooth or colliculate, puberulent; ***seeds*** reddish-brown, 5–10 × 3–5 mm, with white coma 1–2.4 cm long attached to micropylar end — Figs 3–5.

#### Vernacular names.

Chinese: 地梢瓜 (dì shāo guā); Mongolian: Sibir temeen khukh; Korean: 양반풀 (Yang-ban-pul); Russian: Ластовень сибирский (Siberian Lastoven).

#### Distribution.

China, Kazakhstan, North Korea, South Korea, Mongolia, Russia — Fig. 1.

#### Habitat.

Thickets and/or grasses on mountain-slopes, dry valleys, sand-dunes, grasslands, roadsides, flood plains, river banks, farm land; 0–3200 m alt. — Fig. 3.

#### Phenology.

Flowering March–September; fruiting June–October.

#### Notes.

In some online databases, such as World Flora Online (WFO 2022), Plants of the World Online (POWO 2022), the ‘accepted’ name is *Vincetoxicumsibiricum* (L.) Decne. This is incorrect because phylogenetic studies confirmed this species is a member of *Cynanchum* and far from *Vincetoxicum* (Khanum et al. 2016; Hu et al. 2020; Kang et al. 2021). In addition, many online databases (Freiberg et al. 2020; Hassler 2022; POWO 2022; WFO 2022; WCVP 2022) give *C.longifolium* Decne. and/or C.sibiricumWilld.var.triangularilobatum Rassulova & B.A. Sharipova as synonyms of *C.thesioides*. They should be excluded because their types have cordate leaves and they, therefore, belong to Cynanchumacutumsubsp.sibiricum (Willd.) K.H. Rech. (Tsiang and Li 1977; Rasulova and Sharipova 1984).

Freyn (1890) based *V.thesioides* on *Ferdinand K. Karo 127* with flowers and young fruits collected during July and August 1888 from desert places around Nerczynsk. Although Freyn did not give a direct indication of the herbarium, he had seen three sets of Karo’s specimens (Freyn 1889: 356): the first set kept in his private herbarium which was purchased in 1905 by the Moravian Museum in Brno (BRNM, Sutorý 2012); the second set kept by Ladislav Josef Čelakovský in the National Museum in Prague (PR); the last set kept by Josef Emmanuel Kabát which is now also kept at PR (Stafleu and Cowan 1979: 482). The only specimen bearing the number *127* is that in BRNM (Fig. 2A) and is, thus, the holotype of *V.thesioides*. Another two specimens in PR (PR793598, PR793599) collected by Karo do not have the number *127* on them, but were also annotated by Freyn as *Vincetoxicumthesioides*. They may be isotypes, but equally, they may have been other gatherings made by Karo on different dates or in another place near Nerczynsk. Consequently, they are not considered here to be isotypes.

Maximowicz (1877) mentioned in the protologue of V.sibiricumvar.australe a collection by *N.M. Przewalsky* from the Ordos land towards the Yellow River (“Hoang-ho”) in Nei Mongol, China and a collection(s) from Beijing without any further information. We have selected Przewalsky’s specimen at LE as the lectotype following unpublished annotations by N. Imkhanitzkaya (Fig. 2B).

In the protologue of Vincetoxicumsibiricumf.linearifolium Debeaux, specimens collected by Debeaux from “sables maritimes de la presqu’ile de Yan-tai” and “dunes de Fou-chan-yen” with flowers on 14 July and fruits on 23 August [1860] were cited. We have found five sheets of Debeaux’s specimens at P. Of these, one sheet with flowering plants (barcode P03872669) and fruiting plants (barcode P00877371) was annotated by Debeaux as “*Vincetoxicum sibiribicum* Dec. forma *foliis augustioribus*”. We designate the flowering plants from this sheet as the lectotype (Fig. 2C).

#### Selected specimens examined.

**China**: Beijing, *C.F. Zhang 5588* (HIB); Shandong, *C.Y. Chiao 2878* (E, IBK, IBSC, NAS, PE); Shaanxi, *Y.W. Tsui 10389* (CDBI, KUN, PE); Sichuan, *Guangyuan Exped. 6001* (SM); Xinjiang, *G.L. Zhu et al. 6689* (NAS, PE, WUK). **Kazakhstan**: Lake Zaysan, *Anonymous s.n.* (E, P). **North Korea**: Pyongyang, *Pyongyang Bot. Garden s.n.* (PE [01572927]); Nampo, *U. Faurie 736* (P). **Mongolia**: Arkhangai, *I.A.Gubanov 341* (MW); Dornod, *I.A. Gubanov 5729* (MW); Dornogovi, *I.A. Gubanov 5196* (MW); Govisümber, *G.N. Ogureeva s.n.* (MW); Khentii, *I.A. Gubanov 10122* (MW). **Russia**: Far East. Amur, *E. Boyko & V. Starchenko s.n.* (RSA [RSA0286750]); Zabaykalsky, *F.K. Karo 359* (E, P, WIS). Siberia. Altai Republic, *T.S. Elias et al. 4394* (NY, PE, RSA); Irkutsk, *H.H. Iltis et al. 252* (NY, WIS); Tuva, *V.V. Nikitin et al. 1268(2)* (PE) [For a full specimens examined see Suppl. material 1].

## ﻿Discussion

*Cynanchumgobicum* was first placed in the genus *Antitoxicum* (Grubov 1955), an illegitimate replacement name for *Vincetoxicum* and was then transferred to *Cynanchum* (Grubov 2000). It was distinct from *C.thesioides* because of the long and scandent stem, broader lanceolate leaves and puberulent, but not glabrous outer surface of the corolla (Grubov 1955, 2000, 2002). However, we found those diagnostic characters to be within the range of variation of *C.thesioides*. Vegetatively, *C.thesioides* varies considerably (Figs 3, 4) over its wide range of habitats from sandy seasides to steep, crumbling, mudstone slopes at elevations of more than 3000 m (Tsiang and Li 1974: 101). This variation and wide variation also in floral parts was observed by previous taxonomists (Maximowicz 1877; Freyn 1890; Komarov 1905: 291; Tsiang and Li 1974; Ma 1980; Li et al. 1995) and reconfirmed by us (Figs 4, 5). We could not find any other significant differences separating *C.gobicum* from *C.thesioides*. Thus, we place *C.gobicum* in synonymy under *C.thesioides*.

The name Vincetoxicumsibiricumf.linearifolium Debeaux has been neglected since its publication. Debeaux (1877) described it from specimens collected in Tché-foû (now part of Yantai City, Shandong Province), China. Its narrowly linear-lanceolate leaves are narrower than the typical ones from Siberia. This form is placed here in synonymy because leaf shapes and sizes vary greatly and continuously in *Cynanchumthesioides* (Fig. 4C).

Floral dimorphism was observed in certain plants of *Cynanchumthesioides*. This dimorphism takes the form of the style-head exceeding the corona lobes (Figs 4I, K, 5A, D) or the style-head covered by the corona lobes (Figs 4J, L, M, 5B, C). In the former case, the corona lobes are erect or slightly bent over the style-head. In the latter case, the corona lobes are slightly to greatly bent towards the centre of the style-head. This is similar to distyly in simple flowers where one flower has a long style and short stamens, but other flowers have a short style and long stamens. We also found that flowers from the same inflorescence usually have the same floral morphology, though sometimes with varying colours (Fig. 5F). From the photographs in PPBC, short style-heads seem to be rarer. The purpose of these dimorphic flowers is unknown. In other Chinese species of *Cynanchum*, the lengths of corona lobes relative to the gynostegia have been described as either longer or shorter than or as long as the gynostegium; only *Cynanchumofficinale* has been described with slightly variable corona lobes, as long as or slightly longer than the gynostegium (Li et al. 1995).This character had been used to distinguish sections in *Cynanchum* (Tsiang and Li 1977), but these sections were not supported by recent molecular phylogenetic studies (Liede and Täuber 2002; Khanum et al. 2016).

## Supplementary Material

XML Treatment for Cynanchum thesioides

## Supplementary materials

Supplementary material 1 A full list of specimens and selected observations of *Cynanchumthesioides* examined Data type occurrence and distribution https://binary.pensoft.net/file/796993This dataset is made available under the Open Database License (http://opendatacommons.org/licenses/odbl/1.0/). The Open Database License (ODbL) is a license agreement intended to allow users to freely share, modify, and use this Dataset while maintaining this same freedom for others, provided that the original source and author(s) are credited.Cai-Fei Zhang, Dong-Juan Zhang, Miao Liao, Guang-Wan Hu

Supplementary material 2 Holotype of Cynanchumsibiricumvar.gracilentum (TI [TI00204077]) Data type image (jpg file) https://binary.pensoft.net/file/796994This dataset is made available under the Open Database License (http://opendatacommons.org/licenses/odbl/1.0/). The Open Database License (ODbL) is a license agreement intended to allow users to freely share, modify, and use this Dataset while maintaining this same freedom for others, provided that the original source and author(s) are credited.Cai-Fei Zhang, Dong-Juan Zhang, Miao Liao, Guang-Wan Hu

Supplementary material 3 Holotype of Cynanchumsibiricumf.hypopsilum (TI [TI00204078]) Data type image (jpg file) https://binary.pensoft.net/file/796995This dataset is made available under the Open Database License (http://opendatacommons.org/licenses/odbl/1.0/). The Open Database License (ODbL) is a license agreement intended to allow users to freely share, modify, and use this Dataset while maintaining this same freedom for others, provided that the original source and author(s) are credited.Cai-Fei Zhang, Dong-Juan Zhang, Miao Liao, Guang-Wan Hu

Supplementary material 4 Holotype of Cynanchumsibiricumvar.latifolium (TI [TI00204080]) Data type image (jpg file) https://binary.pensoft.net/file/796996This dataset is made available under the Open Database License (http://opendatacommons.org/licenses/odbl/1.0/). The Open Database License (ODbL) is a license agreement intended to allow users to freely share, modify, and use this Dataset while maintaining this same freedom for others, provided that the original source and author(s) are credited.Cai-Fei Zhang, Dong-Juan Zhang, Miao Liao, Guang-Wan Hu
