# Supplementary material for: ﻿A taxonomic revision of Cynanchumthesioides (Apocynaceae) with two new synonyms
Source: PhytoKeys. 2023 Jan 19;219:11–25. doi: 10.3897/phytokeys.219.93514 (PMC10210048; doi:10.3897/phytokeys.219.93514)
Supplement: Supplementary material 1 — A full list of specimens and selected observations of Cynanchumthesioides examined [file phytokeys-219-011_article-93514__-s001.pdf]

A full list of examined specimens and selected observations of *Cynanchum thesioides*

Cai-Fei Zhang[1,2], Dong-Juan Zhang[1,2], Miao Liao[1,2], Guang-Wan Hu[1,2,3]

1 *CAS Key Laboratory of Plant Germplasm Enhancement and Specialty Agriculture, Wuhan Botanical Garden, Chinese Academy of Sciences, Wuhan 430074, China* **2** *Sino-Africa Joint Research Center, Chinese Academy of Sciences, Wuhan 430074, China* **3** *University of Chinese Academy of Sciences, Beijing 100049, China*

I. CHINA.

I1. Anhui Prov. Bengbu: Huaiyuan County, Huaihe Jingtu Gorge Carp and Longsnout Catfish Reserve, 33.958°N, 118.209°E, 26 m, roadside, 17 Aug. 2016 (fl.), *Jian-Wen Shao et al. ANUB00646* (ANUB [ANUB000759]). **Bozhou City**: Huatuo Town, Lizhuang, 40 m, sandy land, 23 Aug. 2007 (fl.), *Ce-Ming Tan et al. 07623* (JJF [JJF00026778, JJF00026779], SZG [SZG00049138]). **Chuzhou City: Dingyuan County,** Huangshi Jinxiu, 2 July 1959 (fl. & fr.), *Bengbu Dingyuan Exped. 126* (NAS [NAS00208879]); Ibid., 2 July 1959 (fl.), *Bengbu Dingyuan Exped. 129* (NAS [NAS00208878]); **Quanjiao County,** Tongjing, farmland side, 14 Sept. 1951 (fl.), *NAS Colleagues 3717* (NAS [NAS00208881, NAS00208875], KUN [0267390], PE [01023759]). **Fuyang City: Funan County,** 6 Oct. 1960 (fr.), *Anhui Funan Exped. 34* (NAS [NAS00208880]). **Hefei City: Chaohu City,** vicinity of Zhemao, in the farmland, 18 Sept. 1951, *NAS Colleagues 3744* (PE [01023760, 01023761]). **Suzhou City: Dangshan County,** Guoyuanfang, Sifenfang, in soybean farmland, 6 Oct. 1960 (fr.), *Anhui Dangshan Exped. 0020* (NAS[NAS00208877]); **Xiao County**: Tiger Hill, side of farmland, 10 July 1957 (fl.), *Fang-Xun Liu et al. 10083* (NAS [NAS00208876], PE [01023765]); Huangzangyu, in grasses on mountain slope, 7 Sept. 2008 (fl.), *Kang Ye et al. YZH-194* (NAS [NAS00597041]) *& YZH-195* (NAS [NAS00597042]); Tianmen Temple, 7 Sept. 2008 (fl. & fr.), *Kang Ye et al YZH- 277* (NAS [NAS00597040]). **Unknown:** “Busu”, *Anonymous 272* (PE [01023398]).

I2. Beijing. **Dongcheng District:** Temple of Heaven, 10 Aug. 1906 (fl.), *Y. Yabe s.n.* (NAS [NAS00208853]); Zuo’anmen, Aug. 1905 (fr.), *Y. Yabe s.n.* (NAS [NAS00208861]). **Changping District:** Nankou to Longtan, Roadside, 27 May 1975 (fl.), *236-6 Team Exped. 0076* (PE [01023839]). **Chaoyang District:** Shuangqiao Farm, roadside, 8 July 1953 (fl.), *Xiu-Qin Shi 2040* (PE [01023588]). **Daxing District:** Nanyuan, Nanhaizi, Milu Garden, in forest, 19 Aug. 1985 (fl.), *Bo-Sheng Li 037* (PE [01647803]). **Fangshan District:** Shangfangshan, 18 July 1933 (fl.), *K.M. Liou 888* (PE [01023645, 01023612, 01023326]); Ibid., 2 Aug. 1933 (fr.), *K.M. Liou 1118* (PE [01023324, 01023644]); Liuli River, July 1935 (fl.), *Ying Liu 10525* (PE [01023594]); Zhoukoudian, Mt. Longgu, 39°41′19.6″N, 115°55′28.1″E, 124 m, 11 July 2008 (fl.), *Zhoukoudian Exped. 53* (PE [02112140]). **Haidian District:** vicinity of Reclining Buddha Temple, 1955, *Botanical Garden Team 041* (PE [01023567, 6010237]); Jinshan, 650 m, roadside, 16 Aug. 1986 (fl.), *Zhong-Tao Wang et al. 278* (L [L.2723149]); Jinshan, no date (fl.), *Shi-Yuan He s.n.* (FSU [00051140]); Jinshan Ying, Jiufeng, on barren slope, in grasses, 22 May 1964 (fl.), *Woon-Young Chun 12304* (IBSC [0519244, 0519279]); Xijiao Park, 1952 (fl.), *Jia-Wen Feng s.n.* (PE [01023563]); Western hills, 1964 (fl.), *Jing-Quan Tian s.n.* (PE [01828828]); Western Hills, Huan-Ku-Yuan, 2 Aug. 1930 (fl.), *Kun-Tsun Fu 206* (HIB [0101693], IBSC [0519250]); Fan Institute, 5 May 1936 (fl.), *De-Fu Jin K.212* (PE [01023826]); Botanical Garden of Peiking, 24 May1935 (fl.), *Si-Tchen Wang 96* (PE [01023693], WUK [0020690]); Yuanmingyuan, Daobian, 18 Aug. 1953 (fl. & fr.), *Fu Zhao 435* (KUN [0267366], PE [01023647], RSA [RSA0286736], TIE [00026707]); Campus of Peking University, 26 June 1957 (fl.), *Shi-Pei Kong & Fu-Xiu Gong 178* (PEY [PEY0025514]); Jinshan, 24 June 1956, *II(4) Team 17* (PEY [PEY0025515]); Hongshantou, hillside, 24 June 1957 (fl.), *Anonymous 222* (PE [01478383]);

Western Hills, Temple of the Reclining Buddha, back hill of Xitong Valley Village, in grasses on the hillside, 6 Sept. 1955 (fr.), *Temple of the Reclining Buddha Exped. 302* (PE [01478388, 01478389, 01478390]); Heishibao, on sunny hillside, 9 Aug. 1955, *Temple of the Reclining Buddha Exped. 116* (PE [01478391, 01478392, 01478393]); Western Hills, Lishigou, on northern slope, 25 May 1955 (fl.), *Temple of the Reclining Buddha Exped. 41* (PE [01478394]); Western Hills Orchard, roadside, 8 Aug. 1959 (fl.), *Jing-Quan Tian 199* (PE [01478395]); Western Hills, Reclining Buddha Temple, in sunning grassland, 28 June 1956 (fl.), *Anonymous 562* (PE [01478396]); Zhou Family Garden, in sunny plain land, 10 July 1956 (fl.), *Chang-Jiang Liu et al. 619* (PE [01478397]); Dong-bai-wang, in northern-exposed mountain woodland, 4 May 2012, *R. Prasse s.n.* (B [B 10 0725945]); Xiaoxishan, 21 May 2019 (fl.), *Cai-Fei Zhang 5588* (HIB); Beijing Botanical Garden of CAS, 70 m, in waste land, 15 July 2022 (fl.), *Meng Wei* for *Cai-Fei Zhang 6791* (Figure 5: A, D, F, G and J; HIB); Western Hills, near Huan-Ku-Yuan, 2 Aug. 1936 (fl.), *T. Tohen 19* (HIB [0101698]). **Huairou District:** Liulimiao Temple, western slope to Laogongying, 460 m, in sandy soil, in rock crevice, on the upper hillside, 27 May 1964 (fl.), *Beijing Mountain Exped. 71* (HNWP [131762], PE [01245513]); Nianzi, Wangjiaying, 21 June 1964 (fl.), *Beijing Mountain Exped.* 248 (HNWP [136814], PE [01245514]); Back hill of Forest Farm of Science Park, Beijing University of Agriculture, 40°53′8″N, 116°26′15″E, 851 m, 30 June 2019 (fl.), *Yu Sun & Xiao-Chun Ren BJTC009528* (BJTC [BJTC009528]). **Mentougou District:** Xiaolongmen, 39°57′18″N, 115°26′04″E, 1250-1350 m, in badly disturbed secondary mixed deciduous forest, 17 July 1996 (fl.), *D.E. Bofford et al. 27115* (IBSC [0519278], E [E00074683, E00068145]); Po-Hua-Shan, Aug. 1936 (fl.), *T.F. King 404* (AU [AU047565]); Zhaitang, 21 July 1957 (fl.), *Anonymous 100* (PE [01023608]); Baihua Mountain, 1200 m, among the dry fields in the sun, 26 July 1956 (fl. & fr.), *Chang-Jiang Liu & De-Yu Xing 197* (PE [01478382]); Mt. Jiulong, 10 Sept. 2006 (fr.), photo by Bing Liu (Figure 5E; http://ppbc.iplant.cn/tu/20366). **Miyun District:** Potou, Dadianzi, 1000 m, in forest, 31 May 2014 (fl.), *Xian-Yun Mu et al. 1567* (BJFC [BJFC00085301, BJFC00085299]); Yaoqiaoyu, Yunxiugu, 445 m, *Gang-Min Zhang et al. 070714* (BJFC [BJFC00062407]). **Pinggu District**, Thousand Buddha Cliffs, 14 July 2009 (fl. & fr.), *Xin Zhang 09714046* (BJFC [BJFC00060450]), Bolitai, 11 July 2018 (fl.), photos by Lei Xie (pers. comm.). **Shijingshan District:** Graduate School of Chinese Academy of Sciences, 17 July 2010 (fl.), photo by Xin-Xin Zhu (Figure 4J; http://ppbc.iplant.cn/tu/3732646). **Xicheng District:** Sipianmen, 26 July 1931 (fl.), *H.F. Chow 41374* (PE [01023829]). **Yanqing District:** “Yanquing”, by roadside, 1982 (fl.), *K.S. Chow 82029* (IBSC [0519245]); Dazhuangke, by farmland, 7 June 1971 (fl.), *Beijing Herb. Med. Exped. Yan126* (PE [01204812]); 29 May 2019 (fl.), photos by Ye-Chun Xu (Figure 4: M; http://ppbc.iplant.cn/tu/9748601). **Unknown:** “Pekin”, ex Herb. Fischeri, 1843 (fl.), *Kirilov s.n.* (syntype of *Vincetoxicum sibiricum* var. *australe* Maxim., LE [LE01036911]); “Pekin”, 1843 (fr.), ex Herb. Fischeri, *Kirilov s.n.* (syntype of *Vincetoxicum sibiricum* var. *australe* Maxim., LE [LE01036910]); “Pekin”, no date (fl.), *Tatarinow s.n.* (syntypes of *Vincetoxicum sibiricum* var. *australe* Maxim., LE [LE01036914, LE01036915, LE01036916]); 24 Sept. 1965 (fr.), *Anonymous s.n.* (SM [SM716700592]); Peking Hills, 15 Aug. 1885 (fl.), *W.R. Carles s.n.* (E [E00780688]); Peking, 12 June 1885 (fl.), *W.R. Carles 145* (E [E00780678]); “Pechin”, May 1888 (fl.), *Em. Bodinier 7* (E [E00780683]); Peking, *Hemeling 332* (E [E00780681]); Peking, *Hemeling 409* (E [E00780679]); In collinis prope Peking, Aug. 1864 (fl.), *S.W. Williamson in H.F. Hance* 10510 (P [P05341433]); environs de Pekin, 1865 (fl.), *A. David s.n.* (P [P03872686]); environs de Pekin, 1869 (fl.), *A. David 390* (P [P03872685, P03872668]); mont Pek[in], Aug. 1862 (fl.), *A. David 459* (P [P03872530]); Peiping, 18 Aug. 1935 (fl.), *S.T. Wang 239* (HIB [0101697], IBSC [0519248, 0519249]); Prince Park, 9 Aug. 1930 (fl.), *T.N. Liou 1558* (PE [01023651]); Peiping, 1929 (fl.), *F.T. Wang 20223* (IBSC [0519255, 0519256], PE [01023796]);.

I3. Gansu. **Baiyin City:** 14 Aug. 1959 (fl. & fr.), *Ye-Qi He 6182* (PE [01023721], WH [08054697], WUK [0392215]); Wangyan, Jiwei Valley, 1900 m, in ditch bottom, 4 Aug. 1982 (fl.), *Zhao-Ying Yu & Yang-Peng Xu 3391* (WUK [0434975]); **Huining County**, Shaochagou, hillside, 15 Aug. 1955 (fr.), *Yellow River Exped. 5879* (PE [01023724, 01614051]); **Jingtai County**, suburban, 1600 m, by farmland, 12 Aug. 1982 (fl.), *Zhao-Ying Yu & Yang-Peng Xu 3355* (WUK [0435011]); **Jingyuan County**, Wulan Town, 36.5321463°N, 104.7703673°E, 1495 m, by farmland, 6 Aug. 2019 (veg.), *Lei Zhang et al. QTP-LJQ-1337-3061* (KUN [1477013, 1477015]). **Dingxi City: Anding District**, Lang'erwazigou, hillside, 7 July 1956 (fl.), *Yellow River Exped. 1134* (PE [01023746, 01614036], WUK [0081920]); Maziwengou, Hillside, 2 Sept. 1956 (fl.), *Yellow River Exped. 2570* (PE [01023720, 01614050], WUK [0083008]). **Gannan Tibetan Autonomous Prefecture: Têwo County,** near Daigu Temple, 34°00′02″N, 103°55′26″E, 1500 m, in shrubs and grassland on hillside, 25 July 1998 (fl.), *Bailongjiang Exped. 713* (PE [01560446]); **Zhugqu County**, near downtown, 1500 m, dry hillside, 21 July 1964 (fr.), *Ben-Zhao Guo 5465* (WUK [0231710, 0396606]). **Jiayuguan City:** Jiayuguan, Nebigou, 13 July 1959 (fl.), *Shu-Zhen Mao et al. 138* (WUK [0248096]). **Jinchang City: Yongchang County,** 3 June 2017 (fl.), photos by De-Shan Zhao (https://ppbc.iplant.cn/tu/9357317). **Jiuquan City: Suzhou District**, Qingshui Town, Cuijiazhuang, 39.3075994°N, 99.185446°E, 1721.88 m, 7 Aug.2020 (fl.), *Lei Zhang et al. QTP-LJQ-CHNO027-3017* (KUN [1499659]). **Lanzhou City: Anning District**, Shajingyi, 16 May 2021 (fl.), photos by Le-Le Liu (https://ppbc.iplant.cn/tu/8334437); **Gaolan County**, Bami Mountain, 1720 m, river beach, 27 July 1957 (fl.), *Tao River Exped.* 3766 (WUK [0098458]); **Yongdeng County,** Liancheng, Zhulingou, 1900 m, hillside, 20 July 1959 (fl.), *Ye-Qi He 4933* (PE [01023729], WUK [0391467, 0157186]); **Yuzhong District**, Yuzhong Campus of Lanzhou University, photos by Yan-Li Wei (https://ppbc.iplant.cn/tu/8573371). **Linxia Hui Autonomous Prefecture: Yongjing County**, Lianhua, on Yellow River rapids, 1720 m, 27 Jul. 1957 (fl.), *Tao River Exped. 3766* (KUN [0267392], NAS [NAS00208840, NAS00208841, NAS00208843, NAS00208844], PE [01023723], WUK [0098458]). **Longnan City: Cheng County ("Huicheng County")**, Xiguan, 910 m, hillside meadow, 31 Aug. 1958 (veg.), *Zhi-Ying Zhang 380* (WUK [0103167]); **Hui County**, Shizhugou, 1200 m, 15 Aug. 1939 (fl.) , *W.Y. Hsia 6109* (PE [01023768]); **Li County**, without precise locality, 1450 m, 20 June 1936 (fl.), *T.P. Wang 4441* (KUN [0279774], PE [01023767], WUK [0067263]); **Wen County**, Danbao, Yangjiagou, 1300 m, in ditch and roadside, 6 Aug. 1959 (fl.), *Zhi-Ying Zhang 10246* (WUK [0145705]); **Wudu District**, Liangshui, Bailong Port, 1050 m, by farmland, 12 Sept.1958 (fl.), *Zhi-Ping Wei 2372* (WUK [0109036, 0404997]). **Qingyang City:** 1560 m, on the plain of the Loess Plateau, 6 June 1953 (fl.), *You-Wen Cui 10141* (PE [01023345, 01023869]); **Heshui County**, Lianjiabian, south of Yangwan, in mountain forest, 28 June 1987 (fl.), *Li-Pin Liu 0166* (QYTC [QYTC0002331]); Taibai Town, 9 July 2018 (fl.), *Kai-Ning Yang 20180709030* (QYTC [QYTC0004439]); Taibai Town, 35°49′47.09″N, 108°29′ 25.62″E, 1568 m, 5 July 2015 (fl.), *Dong-Liang Luo QYTC20150705017* (QYTC [QYTC0004437]); Ibid., 35°42′20.22″N, 108°20′41.62″E, 1511 m, 23 July 2015 (fl.), *Dong-Liang Luo QYTC20150723026* (QYTC [QYTC0004436]); Ibid., 35°46′48.57″N, 108°29′ 19.45″E, 1469 m, 29 July 2015 (fl.), *Dong-Liang Luo QYTC201507290004* (QYTC [QYTC0004435]); Ibid., 35°41′04.17″N, 108°29′24.56″E, 1573 m, 9 July 2015 (fl.), *Dong-Liang Luo QYTC20150709028* (QYTC [QYTC0004421]); Ibid., 35°49′21.76″N, 108°24′ 04.38″E, 1518 m, 13 July 2015 (fl.), *Dong-Liang Luo QYTC20150713027* (QYTC [QYTC0004412]); 9 July 2018 (fl.); **Huan County**, Tan Sheep Breeding Farm, Bailaozhuang, 1860 m, waste land and grassy slope, 18 July 1981 (fl.), *Zhao-Ying Yu & Yang-Peng Xu 1269* (WUK [0449345, 0432178]); **Xifeng District**, Nanxiaohegou, 20 Sept.

1954 (fr.), *T.P. Wang 17533* (WUK [0070972]); **Zhengning County**, Zhongwan Forest Farm, 35°26′50.13″N, 108°33′18.66″E, 1465.9 m, 17 July 2014 (fl.), *Dong-Liang Luo QYTC20140717027* (QYTC [QYTC0004438]); **Zhenyuan County**, Tiebashengou, 1300 m, in ditch, 25 Aug. 1953 (fr.), *T.P. Wang 17273* (WUK [0070900]). **Pingliang City: Huating County**, suburben, 1520 m, by roadside and in grasses, 28 July 1953 (fl.), *T.P. Wang 17011* (WUK [0070966]); **Jingchuan County**, Hejiaping, 1000 m, village side, 22 June 1953 (fl.), *T.P. Wang 16784* (WUK [0070967]); **Kongtong District**, Kongtong Mountains, hillside, 7 July 1956 (fl.), *Yellow River Exped. 1886* (NAS [NAS00208846], PE [01023739, 01614049], WUK [0082678]). **Tianshui City: Gangu County**, Xingshan Temple, 6 Aug. 1940 (fl.), *Wei-Ying Hsia 8069* (WUK [0079770]); **Qinzhou District**, Lv'ergou, on dry slope, 15 Aug. 1950 (fl.), *Zhong-Lun Wu 20009* (PE [01023735]). **Wuwei City: Tianzhu Tibetan Autonomous County**, Qilian Township, 37°43′00.44″N, 102°25′51.63″E, 2213 m, roadside and dry mountain slope, 4 July 2014 (fl.), *Jie Cai et al. 14CS9296* (KUN [1375681]). **Zhangye City: Ganzhou District**, Huazhai Township Z075, 38.6765211°N, 100.3784325°E, 1882.51 m, 14 Aug. 2020 (fl. & fr.), *Lei Zhang et al. QTP-LJQ-CHNO028-3190* (KUN [1499660]); Jing'an, 39.0771242N, 100.352582E, 1404.9 m, *Lei zhang et al. QTP-LJQ-CHNO028-3108* (KUN [1499657]); **Sunan Yugu Autonomous County**, Dahe, Shaquan, 2400 m, by ditch, 5 Aug. 1967 (fr .), *Hexi Exped. 141* (PE [01023725, 01023726, 01023727, 01023730]); **Shandan County**, North Mountain, 1800 m, hillside, 27 Aug. 1958 (fr.), P.*C. Tsoong 8757* (PE [01023346, 01023722], WUK [0101200]). **Unknown:** a strange pod. from hot dry loers banks on the Akropolis of Siku, Sept. 1916 (fr.), *R. Farrer & W. Purdom 327* (E [00780673]); Excursion a la source de petrole de Che yeou Ho (Nan-Chan), 2300 m, 18 June 1908 (fl.), *Pelliot & L. Vaillant 733* (P [P03872513, P03872514]).

I4. Hebei. **Baoding City: Fuping County**, 39°3′16.57″N, 114°21′33.5″E, 942 m, roadside, 24 July 2015 (fl.), *Rong Yang YR20150724-1* (BNU [0018588]); **Jingxiu District**, Yimuquan, sunning place on plain, 23 June 1959 (fl.), *No.4 4033* (PE [01023620]); **Laiyuan County**, Mt. Baishi, 1180 m, 11 July 1959 (fr .), *Anonymous 3131* (HNWP [136793]); **Yi County**, Xiling, vicinity of Guanzuo Ridge, hillside, 9 June 1953 (fl.), *Fu Zhao 243* (KUN [0267368, 0267369], PE [01023599]); Angezhuang, 39.247°N, 115.248°E, 152 m, 3 Oct. 2019 (fr.), *BNU Exped. 20191003YX006* (BNU [0049842]);. **Chengde City:** Without precise locality, 19 Aug. 1933 (fl.), *T. Nakai, M. Honda & M. Kitagawa s.n.* (TI [TI00204077], holotype of *Cynanchum sibiricum* var. *gracilentum*); Ibid., 19 Aug. 1933 (fl. & fr.), *T. Nakai, M. Honda & M. Kitagawa s.n.* (TI [TI00204078], holotype of *Cynanchum sibiricum* var. *gracilentum* f. *hypopsilum*); Ibid., 1959 (fl. & fr.), *Nankai Univ. 0252* (PE [01023557, 01023609, 01023610, 01023872, 01023601, 01023816]); Ibid., 20 June 1971 (fl.), *Chengde Exped. 4* (PE [01245523, 01245526]); **Longhua County**, Mt. Dayakou, May 1984 (fl.), *Duan-Zheng Lu s.n.* (BJFC [BJFC00017450]); **Pingquan City (former Pingquan County)**, Huba Town, Sijia Village, 1000 m, grasses, 18 July 2013 (fl.), *No.4 Group 130823130718066LY* (HBNU [HBNU10013965]); **Weichang Manchu and Mongolian Autonomous County**, side of farmland, 6 July 1971 (fl.), *Chengde Exped. 253* (PE [01245521]); **Xinglong County**, near the railway station, 3 Aug. 1982 (fl.), *Yong-Sheng Zhang et al. 207* (GXMI [GXMI011390]); Huangyangtan, 13 July 1954 (fl.), *Qing-He Zhang 7* (PE [01023582]); Wulingshan, 950 m, road side, 13 June 1931 (fl.), *K.M. Liou 239* (PE [01023561]). **Handan City: Ci County**, Mt. Lufeng, hillside, 24 July 1958 (fl.), *Ke-Jian Guan 6069* (PE [01023836], TIE [00026704]); **Cheng'an County**, to Beixiangyi, 100 m, by the canal, 1 June 1972 (fl.), *Anonymous Han144* (PE [01245515]); **Quzhou County**, 25 May 1989 (fl.), *Yi-Qiao Liu 875018* (BJFC [BJFC00017453]); **She County**, near Qingta, 26 June 1958 (fl.), *Ke-Jian Guan 5643* (PE [01023838], TIE [00026702]); Ibid., 26 June 1958 (fl.), *Ke-Jian Guan 5659* (TIE [00026701]); **Wu'an City (former Wu'an County)**, from Majiazhuang to Dashui, 14 July 1958 (fl. & fr.), *Ke-Jian Guan 5920* (PE [01023837], TIE

[00026705]); Majia, hillside, 12 June 1972 (veg.), *Handan Exped. 324* (PE [01245522]); **Yongnian District (former Yongnian County)** , Yonghehui Town, Chenyao Village, 234 m, in grasses, 12 Aug. 2013 (fl.), *No. 15 Group 130429130812001LY* (HBNU [HBNU10013999]). **Hengshui City: Taocheng District**, Binhu Development Zone, wasteland near Hengshui Lake, 20 m, grassland and wetlands near the lake in the temperate plain, 15 Sept. 2018 (fr.), *Yu-Lu Niu et al. NiuYL663* (KUN [1454131]). **Qinhuangdao City:** "Tsin Wang tao", July 1916 (fl.), *P. Courtois 23556* (NAS [NAS00208854]); 7 Sept. 1971 (veg.), *Anonymous 1639* (PE [01245517]); **Beidaihe District**, 25 Aug. 1951 (fl. & fr.), *F.T. Wang 0343* (PE [01245499]); Geziwo, hillside, 25 Aug. 1982 (fl.), *Cai-Ling Wang & Xiang-Nong Dong 0859* (TIE [00026709, 00026710]); Niutou Cliff, earth slope, 19 Aug. 1959 (fl. & fr.), *F.T. Wang 26* (PE [01023652, 01023325], TIE [00026706]); Beidaihe seashore, roadside among farmlands, 27 Aug. 1980 (fl. & fr.), *Cheng-Yun Duan Jin-00610* (TIE [00026700]); Southeast of Nanda Temple, sand Dunes to the seaside, 20 June 1952 (fl.), *Xue-Yu Hou 20165* (PE [01553830]); front of the guest house of Beidaihe Academy of Sciences, 15 June 1952 (fl.), *Xue-Yu Hou 20018d* (PE [01553831]); **Changli County**, at the foot of the mountain outside the north downtown, 14 July 1950 (fl.), *W.T. Wang et al. 10425* (PE [01245512]); **Funing County**, Nandaihe River, seaside sand, 11 Sept. 1972 (fr.), *Tangshan Exped. 96* (PE [01245524]); Qinhuangdao City, in front of the guest house of the Chinese Academy of Sciences, seaside, 15 June 1952 (fl.), *Xue-Yu Hou 20016* (PE [01553829]); **Qinglong Manchu Autonomous County**, Mt. Niuxin, 340 m, side of farmland, 3 Sept. 1971 (fr.), *Qinglong Exped. 854* (PE [01245520]); **Shanhaiguan District**, Out of Shanhai Pass, Temple of Lady Meng Jiang, June 1978 (fl.), *C.Y. Wu s.n.* (KUN [0237396]). **Shijiazhuang City:** Urban, west of Xigang Village, 29 Sept. 1955 (fr.), *Ying Liu, Guo-Fang Zhu 45* (PE [01023815]); campus of Hebei Normal University, 65 m, flat, 27 July 2016 (fl.), *Ping-Ping Wan sz4475* (HBNU [HBNU10013349]); **Jingxing County**, Nanhaoting, Nansizhang Forest Farm, gravel crevices in the valley, 23 June 1971 (veg.), *Anonymous 51* (PE [01245518]); **Lingshou County**, Nanyanchuan Township, Laoshugou Village, 235 m, bushes, 25 Aug. 2013 (fr.), *Yu-Ping Yan 130126130825002LY* (HBNU [HBNU10014010]); **Pingshan County**, without precise locality, 800 m, bushes, 20 May 2016 (fl.), *Rong Wang LT0063* (HBNU [HBNU20001473]); without precise locality, 800 m, 2 May 2016 (possible wrong date; fr.), *No. 5 Team ZW0060* (HBNU [HBNU20001472]); **Xinji City**, "Donglu County", Gengzhuang, side of farmland, 13 Sept. 1971 (fr.), *Shijiazhuang Exped. 544* (PE [01245497]); "Sulu Hsien", Xinji ("Hsin Chi"), 5 July 1948 (fl.), *Kay H. Beach 34* (IBSC [0519263]); **Xingtang County**, Jiukouzi Township, Dongsi Village, 236 m, grasses, 4 July 2013 (fl.), *Xiao-Min Yan 130125130704016LY* (HBNU [HBNU10014008]); Koutou Town, Mahua Village, 215 m, in grassses, 15 Sept. 2013 (fr.), *Le Zhang 130125130915326LY* (HBNU [HBNU10014044]); **Zanhuang County**, Zhangshiyan, Upper Dafan Village, 532 m, in grasses, 19 Sept. 2013 (fl.), *No. 2 Group SJX0300* (HBNU [HBNU20001941]). **Tangshan City: Qian'an City (former Qian'an County)**, 96 m, in grasses, 29 July 2014 (fl.), *Yu-Yang Yang 130283140729349LY* (HBNU [HBNU10014042]); **Qianxi County**, Santun, Mt. Jingzhong, 120 m, hillside, 22 July 1972 (fr.), *Qianxi Exped. 453* (PE [01245525]); **Yutian County**, Mt. Shigu, 5 Aug. 2018 (fl.), photos by Mao Li (https://ppbc.iplant.cn/tu/6084763); **Zunhua City**, near Eastern Tomb, 910 m, 15 June 1931 (fl.), *Ki-Mon Liou 281* (IBSC [0519253], PE [01023560, 01023646], WUK [0012168]); Dongling, open slope, Aug. 1935 (fl.), *Ying Liu 11867* (IBSC [0519242], PE [01023596]); Ibid., June 1930 (fl.& fr.), *H.T. Tsai 50079* (IBK [IBK00344713], IBSC [0519254]); Malanku, mountain grassy slope, 26 May 1951 (fl.), *Ying Liu & Jing Zhang 15401* (IBK [IBK00238276], PE [01023584, 01023322]); Ibid., on hills, 26 May 1951 (fl.), *Ying Liu 15418* (IBK [IBK00238278], PE [01023586]); Ibid., road side , 20 May 1951 (fl.), *Ying Liu & Jing Zhang 15389* (PE [01023323, 01023585]). **Xingtai City: Julu County**, Tuancheng Township, 23 m, in grasses, 24 Aug.

2013 (fl.), *Wei Tian 130529130824050LY* (HBNU [HBNU10013925]); **Lincheng County**, Feng Village, 200 m, roadside or grassy land, 14 June 1950 (fl.), *Ying Liu & Xin-Yuan Liu 12789* (PE [01023648]); **Neiqiu County**, Dongyangelao, humid and shady ditch, 25 June 1951 (fl.), *Xin-Yuan Liu & Fu Zhao 492* (KUN [0267371], NAS [NAS00208860], PE [01023813, 02108826, 02108827]); Jiguanzhai, Mt. Tongshan, hillside, 2 June 1951 (fl.), *Xin-Yuan Liu & Fu Zhao 00283* (HIB [0101691], KUN [0267367], PE [01023409, 01023614]); **Shahe City**, Kongzhuang Village, 483 m, in grasses, 24 July 2 013 (fl.), *Xiao-Liang Xie 130582130724205LY* (HBNU [HBNU10014007]); **Xindu District**, Jiangshui Town, Nansigou Village, 400 m, in grasses, 19 July 2013 (fl.), *Wei Tian 130521130719161LY* (HBNU [HBNU10013927]). **Zhangjiakou City: Chicheng County**, Dahaituo, 1280 m, ditch side, 8 Sept. 1959 (veg.), *Anonymous 6518* (PE [01023626, 01023313]); Zhen'anbao, Quanjiagou, 1350 m, top of ditch, 8 Sept. 1959, *Anonymous 4466* (HNWP [136795], PE [01023321, 01023639]); **Chongli District**, Si ouan tze, no date (fl.), *Anonymous s.n.* (NAS [NAS00208958]); **Qiaodong District/Qiaoxi District**, Mt. Taiping, 950 m, sunny slope, 20 Sept. 1959 (veg.), *Anonymous 4707* (PE [01023314, 01023631]); **Shangyi County**, Manketutan, 22 June 2008 (fl.), photos by Shang-De Shi (https://ppbc.iplant.cn/tu/117523]); **Wei County**, Chiyabao, river beach, 1200 m, rocks in sunny place, 17 July 1956 (fl.), *Jun-Xi Duan 615* (PE [01478386]); **Wei County, Mt. Xiaowutai**, Nantaigou, 1300 m, 9 July 1974 (fl.), *K.S. Chow [Gen-Sheng Zhou] 74300* (PE [01023566], WIS [v0395876]); West Terrace, Xijinhe River, 1320 m, shady slope, 25 June 1959 (fl.), *Anonymous 1452* (HNWP [136796], PE [01023634, 01023319], TIE [00026708]); Xiaowutai Forest Farm, farmland side, 25 June 1962 (fl.) , *Anonymous 449* (BJM [0148523, 0239847]); Middle Terrace, Duijugou, 1100 m, both sides of the stream, 16 July 1959 (fl.), *Anonymous 2682* (PE [01023316, 01023641]); South Terrace, Hui Village, 1280 m, sandy river beach, 24 July 1959 (fl.), *Anonymous 5664* (HNWP [136794], PE [01023317, 010123636]); West Terrace, 1400 m, Goubian, 23 Jun 1959 (fl.), *Anonymous 1602* (HNWP [136790], PE [01023318, 01023633]); mouth of Xijinhe River, 1320 m, shady slope, 25 June 1959 (fl.), *Anonymous 1452* (HNWP [136796], PE [01023319, 01023634], TIE [00023708]); East Terrace, Caojiawan, Sizhuangzi, hillside beside the village, 27 July 1959 (fl.) , *Anonymous 3381* (PE [01023320, 01023637]); Huichuan, 1400 m, in the valley, 18 July 1972 (fl.), *Hebei Herbal Med. Zhangjiakou Exped. 510* (PE [01245519]); July-Aug. 1936 (fl.), *Wen-Chen Wu & Cheng-Yuan Yang 37448* (PE [01023554]); Sept. 1935 (fr.), *Cheng-Yuan Yang & Zu-Gui Li 36418* (PE [01023555, 01023606]); **Zhangbei County**, Kangbao, Dayingtu, 1500 m, 20 Aug. 1959 (veg.), *Anonymous 8478* (PE [01023315, 01023640]); **Zhuolu County**, Dong'ansi, 16 July 1951 (fl.), *Chao-Guang Yang 347* (PE [01023593, 01023574]); Lvjiawan, 900 m, 2 July 2016 (fl.), *Ping-Ping Wan wp0023* (HBNU [HBNU10013351]) & *wp0024* (HBNU [HBNU10013350]); Yangjiaping, Hejiagou, 953 m, 10 July 2017 (fl.), *No. 11 Team DC074* (HBNU [HBNU10016533]) & *DC076* (HBNU [HBNU10016535]); Yangjiaping, Beigou, 912 m, 2 July 2017 (fl.), *No. 13 Team DC075* (HBNU [HBNU10016534]). **Unknown:** Tche-Ly, Tiug-Tcheou, July 1910 (fl.), P. *Chunch 586ter* (E [E00780692]); Tche-ly, Ting-Tchou, July 1910 (fl.), *P. Chonet 589gci* (E [E00780691]); "Chili", 1929 (fl.), *C.F. Li 614* (CQNM [0007909], PE [01023799]); "Chili", 1929 (fl.), *F.T. Wang 223* (PE [01023797]); 10 June 1958 (fl.), *Ke-Jian Guan 5340* (PE [01023835], TIE [00026703]); Hopei, 1930 (veg.), *H.F. Chow 41378* (CQNM [0007910]); Hopei, 1930 (fl.), *C.F. Li 11863* (CQNM [0007911]); 14 Aug. 1930 (fl. & fr.), *Licent 9852* (TIE [00053534, 00053535]); 7 Sept. 1971 (fr.), *Chengde Exped. 19*39 (PE [01245516]); Province of Pechiley, *G. Staunton s.n.* (BM [BM001014199]); Géhol, Partout en lieu sec, sur les chemins, June 1864 (fl. & fr.), *A. David 1806* (P [P03872528, P03872531]); Géhol, en lieu sec, bord des chemins sablonneux [in a dry place, along sandy paths], May-June 1864 (fl. & fr.), *A. David 1809* (P [P03872529]).

I5. Heilongjiang. **Anda City:** 13 Sept. 1951 (fr.), *Yu-Liang Zhang et al. 803* (PE [01023513]). **Daqing City: Dulbert Mongolian Autonomous County**, Taikang Town (former Taikang County), Green Grassland Meadow, sand dunes, July 1987 (fr.), *Grade 1986 Students of Animal Husbandry Depart. 87-344* (NEAU [NEAU0000952, NEAU0004512]); Green Grassland Meadow, 143 m, 3 July 2016 (fl.), *Wei Cao CaoW6936* (IFP [IFP0243030, IFP0243083, IFP0243135]); **Lindian County**, No. 5 Branch of Xinxing Ranch, 177 m, grassland, 1 July 2016 (fl.), *Wei Cao CaoW6841* (IFP [IFP0242480, IFP0242433, IFP0242386]); **Ranghulu District**, Lamadian Town, roadside, 194 m, July 1986 (fl.), *Harbin Normal Univ. 8905* (IBSC [0519271]); **Zhaoyuan County**, dry land, Aug. 1981 (fl.), *Xue-Ren Yu s.n.* (NEAU [NEAU0004948]); Wangjiatun, 45.7438°N, 125.1041°E, 141 m, 6 July 2016 (fl.), *Wei Cao CaoW7087* (IFP [IFP0242914, IFP0242992, IFP0242953]); **Zhaozhou County**, Qingshan, 147 m, 3 July 1981 (fl.), *Harbin Normal Univ. 8055* (IBSC [0519272]). **Harbin City:** vicinity of Harbin Agricultural College [currently Northeast Agricultural University], 12 Aug. 1950 (fl. & fr.), *Guang-Zheng Wang 124* (IBSC [0519273], KUN [0267401], NAS [NAS0208848], PE [01023503, 01023523], WUK [0187718]); Harbin, June 1937 (fl.), *Anonymous s.n.* (IBSC [0519280]); **Acheng District**, dry land, June 1982 (fl.), *Anonymous 127* (NEAU [NEAU0000744]); **Shangzhi County**, Mt. Maoer, dry land, July 1964 (fl.), *Qi-Yan Xing & De-Chang Tian s.n.* (NEAU [NEAU0004949]); **Wuchang City**, dry land, 7 Aug. 1980(fl.), *Anonymous s.n.* (NEAU [NEAU0000746]). **Heihe City: Nenjiang County**, Aug. 1980 (fr.), *Qi-Yan Xing s.n.* (NEAU [NEAU0004947]). **Jixi City: Hulin City (former Hulin County)**, July 1987 (fl.), open space, *Gui-Lin Guo s.n.* (NEAU [NEAU0004953]). **Mudanjiang City: Ning'an County**, Jingpo Lake, dry land, July 1981 (fl.), *Gui-Lin Guo & Qi-Yan Xing s.n.* (NEAU [NEAU0004946]). **Qiqihar City:** 0.5 km west of the city, well-drained mound, fine sandy soil, 20 Aug. 1950 (fl. & fr.), *Xue-Yu Hou 10626* (PE [01581588, 01581589]); **Ang'angxi District**, 47.1246°N, 123.8288°E, 145 m, 20 July 2015 (fl.), *Ying-Xin Huang 325* (IFP [IFP0228495, IFP0228564, IFP0228622]); 14 Aug. 2010 (fl.), a photo by Chuan-Qi Shi (https://ppbc.iplant.cn/tu/808002); **Fuyu County**, 27 Aug. 1950 (fr.), *Da-Quan Zhu 0250* (NAS [NAS00208847]); Qunli stud farm, 47.611°N, 124.3263°E, 157 m, 27 July 2015 (fl.), *Ying-Xin Huang 883* (IFP [IFP0229940, IFP0229902]); **Gannan County**, dry land, July 1986 (fr.), *Gui-Lin Guo s.n.* (NEAU [NEAU0004951]); **Longjiang County**, Lahai Township, 158 m, meadow, 21 July 2015, *Ying-Xin Huang 1591* (IFP [IFP0231624, IFP0231571]); **Tailai County**, Chaguluo, lichen soil, 11 Sept. 1950 (fr.), *Xue-Yu Hou 11407* (PE [01581590]). **Suihua City: Mingshui County**, dry land, Aug. 1980 (fl.), *Xue-ren Yu s.n.* (NEAU [NEAU0004945]); **Zhaodong City**, dry land, 10 Aug. 1986 (fr.), *Huai-Liang Shen s.n.* (NEAU [NEAU0004862]). **Yichun City:** Liangshui River, 18 July 1984 (fl.), *Anonymous 304* (NEFI [089001007005001]).

I6. Henan. **Anyang City: Huaxian County**, Gaoping, 16 July 2008 (fl.), Sui-*Gen Xu*13 (HENU [1003298]); **Linzhou City (former Lin County)**, Taoyuan, Western Hill, 600 m, 30 June 1983 (fl. & fr .), *Loess Team 776* (WUK [0446484, 0446485]); Linqi Town, 7 Aug. 2008 (fl.), *Pei-fang Wan 1* (HENU [1003295]). **Hebi City: Xunxian County**, Shantang Town , 9 July 2008 (fl.), *Bing-Xiao Sun 7* (HENU [1003287]). **Jiyuan City:** Mt. Tiantan, 100 m, July 1996 (fr.), *Xiao-Quan Zhao 468* (HENU [1008566]); Ibid., 200 m , hillside, July 1996 (fl.), *Li-Zhi Lv 709* (HENU [1008567]); Macaque Reserve, Neem Forest Farm to Heilonggou, 30 July 2015 (veg.& fl.), *Chang-Shan Zhu et al.150730062* (AU [AU068618, AU064538]). **Jiaozuo City:** July 1956 (fl.), Xu Cuixin et al. 24; 10 Oct. 1928 (fl.), Anonymous 738 (PE [01023684]); **Qinyang City:** 90 m, July 1997 (fl.), *Xiao-Quan Zhao 214* (HENU [1008564]); **Xiuwu County:** Yuntai Mountain, 110 m, July 1997 (fl.), Zhao Xiaoquan 213 (HENU [1008565]); Yuntai Mountain, 35°26.647′N, 113°18.421′E, roadside grass, 18 June 2009 (fl.), *Yuntai Mountain Collection Team 955* (PE [02026207]); cloud

Taishan Baijiayan, Mountain Roadside Grass, 16 Aug. 2009 (fl. & fr.), *Yuntai Mountain Collection Team 1026* (PE [02022582, 02022583]). **Kaifeng City**: "Kaifing", 22 June 1932 (fl.), *Kin-Shen Hao 3474* (PE [01023332, 01023687, 01023686]; Northern of Kaifeng City, north of Yellow River, 28 Aug. 1952 (fl.), *Xian-Pu Wang 20349* (PE [01552346]); Northwest suburbs, Aug. 1961 (fl. & fr.), *Cui-Xin Xu et al. 214* (HENU [0170214]); Aug. 1961 (fl. & fr.), *Cui Lu 211* (HENU [0170211]); Oct. 1954 (fr.), *Cui Lu 401* (HENU [0170212]); 19 Aug. 2008 (fl.), *Ming-Zhe Mao 12* (HENU [1003291]); 2 Aug. 2008 (fl.), *Guo-Long Liu 13* (HENU [1003289]); 24 Aug. 2008 (fl.), *Ming-Zhe Mao 23* (HENU [1003297]); 21 Aug. 2008 (fl.), *Ming-Zhe Mao 9* (HENU [1003293]); 23 Aug. 2008 (fl.), *Ming-Zhe Mao 16* (HENU [1003292]); 20 June 1932 (fl.), *K.S. Hao 3457* (PE [01023685]). **Luoyang City:** 7 Aug. 2008 (fl.), *Xiao-Cui Yang 3* (HENU [1003294]); **Jili District**, Jili Township, 15 Aug. 2018 (fl.), *Wen-Na Fan 67* (HEAC [HEAC0020061]) *& 678* (HEAC [HEAC0020076]); **Yiyang County**, 730 m, wet land in the shade of the mountain, 14 Aug. 1959 (fl. & fr.), *Kaifeng Teachers College 22012* (PE [01023690]); **Song County**, southwest of downtown, Yang Village, 530 m, in the middle of the valley, 10 Sept. 1959 (fl. & fr.), *Anonymous 35239* (PE [01023335, 01023336]). **Nanyang City: Neixiang County**, Mashankou, 7 July 1960 (fl.), in plain, *Ke-Jian Guan & Tian-Lun Dai [Henan Exped.] 893* (PE [01023689, 01647795]); **Tanghe County**, Heilong Town, Zhao Village, 10 Aug. 2018 (fl.), *Hao-Zhe Yang 30001* (HEAC [HEAC0020657]); **Tongbai County**, Funiu Mts, Tanghe, Xigang, sunny place, 23 Aug. 1956 (fl.), *Henan Forestry Department 522* (PE [01023337, 01023338]); Tongbai County, Funiu Mts., West of Wucheng, hillside and waterside, 8 Aug. 1956 (veg.), *Henan Forestry Department 152* (PE [01023339]); **Wolong District**, Dushan, Hillside, 28 Aug. 1956 (fl.), *Henan Forestry Department 558* (PE [01023340, 01023341]). **Pingdingshan City: Jia County**, Ciba Town, 1 Sept . 2018 (fl.), *Xiao Guo 1156* (HEAC [HEAC0020423]); **Ruzhou City**, Mangchuan, Wangling Village, 3 Sept. 2018 (fl.), *Xiao Gu 91* (HEAC [HEAC0020570]); without precise locality, 11 July 2008 (fl.), *Rui-Rui Pan 6* (HENU [1003296]). **Sanmenxia City: Lushi County**, Wulichuan, Songjiazhuang, 800 m, grassland at the foot of the mountain, 24 July 1959 (fl.), *Anonymous 34344* (PE [01023691, 01023692]). **Xinxiang City: Huixian County**, Mt. Wanxian, 6 July 2010 (fl.), *Shi-Chuang Wang 4* (HEAC [HEAC0002892]) *& 7* (HEAC [HEAC0002895]); Ibid., 5 July 2015 (fl.), *Bio. Sci. 2nd Class 41* (HEAC [HEAC0012152, HEAC0012153]); Ibid., 3 July 2013 (fl.), *Biol. Sci. 2012 Class 852* (HEAC [HEAC0001639]); Ibid., 3 July 2011 (fl.), *Bio. Sci. 2010 Class 546* (HEAC [HEAC0001341]); Ibid., 4 July 2011 (fl.), *Bio. Sci. Class 4650* (HEAC [HEAC0000689]); Ibid. , 3 July 2011 (fl.), *Bio. Sci. Class 350* (HEAC [HEAC0000861]); Ibid., 3 July 2012 (fl.), *Bio. Sci. Class 433* (HEAC [HEAC0001034]); Ibid., 4 July 2015 (fl.) , *Bio. Sci. Class 113* (HEAC [HEAC0010919]); Ibid., 4 July 2015 (fl.), *Bio. Sci. Class 09* (HEAC [HEAC0012303]); Ibid., 4 July 2015 (fl.), *Bio. Sci. Class 57* (HEAC [HEAC0012590]); Ibid., *Bio. Sci. Class 28* (HEAC [HEAC0017888]); Ibid., 4 July 2015 (fl.), *Bio. Sci. Class 32* (HEAC [HEAC0017889]); Ibid., 3 July 2018 (veg.), *Bio. Sci. 1st Class No. 1 Team 8* (HEAC [HEAC0018760]); Ibid., 1 July 2018 (fl.), *Bio. Sci. 2016 Class No. 3 Team 62* (HEAC [HEAC0019045]); Ibid., 3 July 2017 (fl.), *Bio. Sci. 1st Class No. 2 Team 65* (HEAC [HEAC0014762]); Ibid., 2 July 2017 (fl.), *Bio. Sci. 2st Class No. 2 Team 2* (HEAC [HEAC0014085]); Ibid., Kunlunpo, 30 June 2019 (fl.), *3-1 Team 70* (HEAC [HEAC0021234]); [Wanxian Mountain] Jiangjun Peak, 4 July 2015 (fl.), *Bio. Sci. 125* (HEAC [HEAC0012294]); **Fengqiu County**, Yingju, 5 Sept. 1964 (fl.), *N. Henan Veg. Exped. 114* (HENU [0170205], HNWP [136816], PE [01023853]); Lizhuang, wet place and roadside, 30 July 1964 (fl.), *N. Henan Veg. Exped. 54* (HENU [0170213], HNWP [136817], NAS [NAS00208869], PE [01023688]); **Yuanyang County**, 20 Aug. 2018 (fl.), *Shan-Shan Shen 20180820* (HEAC [HEAC0025822]); **Laihe District,** Jigong Mts., Dadonggou, 11 July 1989 (veg.), *Duan-Zheng Lu 198907* (BJFC [BJFC00017466]). **Xuchang City: Yuzhou**

City, 6 Aug. 2008 (fl.), *Tian Feng 9* (HENU [1003288]). **Zhengzhou City:** 10 Aug. 2008 (fl.), *Zhe-Yi Yang 13* (HENU [1003299]); **Dengfeng City**, Taishi Mountain, 750 m, 21 June 1984 (fl.), *Loess Plateau Exped. 3045* (WUK [0456289, 0456290]); 28 July 2018 (fl.), *Yun-Xia Zhang 1203* (HEAC [HEAC0020170]); **Jinshui District**, Forest Park, 25 May 2009 (fl.), *Pl. Pathology 2nd Team* 8174 (HEAC [HEAC0008174]); **Dancheng County**, Fishing Farm, 33°52′N, 115°20′E, 40 m, 28 Sept. 2009 (fr.), *Jin-Ting Dong HNP002* (AU [AU078059]); 29 Aug. 2018 (fl.), *Yan-Ling Li 1023* (HEAC [HEAC0020106]); **Huaiyang District (former Huaiyang County)**, 1935 (veg.), *Tsui Hwa 40* (PE [01023854]). **Zhoukou City: Xihua County**, 8 July 2008 (fl.), *Yi-Lei Zhao 12* (HENU [1003290]). **Unknown:** Im Kreise Yen Shih, Aug. 1907 (fl.), *A.K. Schindler 195* (E [E00780687], L [L.2723147]); 2 June 1958 (fl.), *Cui-Xin Xu 008* (HENU [0170203]); 18 May 1958 (fl.), *Cui-Xin Xu 200* (HENU [0170206]); 9 July 1959 (fl.), *Xiao Fang 71* (HENU [0170207]); 24 Jul y 1959 (fl.), *Xiao Fang 615* (HENU [0170208]); July 1958 (fl.), *Xiao Fang 51133* (HENU [0170209]); 11 Sept. 1959 (fl. & fr.), *No.3 Team 151133* (HENU [0170215]); 19 Aug. 1959 (fr.), *No.3 Team 89* (HENU [0170216]); no date (fr.), *Anonymous 18584* (NAS [NAS00208871]); *Anonymous s.n.* (PE [01023683]); *Anonymous 2* (PE [01023682]).

I7. Hubei. **Danjiangkou City (former Jun County):** Wudang Mts., Zixiao Hall, Chenkouwo, 780 m, hillside, 7 Aug. 1959 (fl.), *Ji-Qing Xing 10621* (WUK [0129021, 0385575]). **Enshi City:** intentionally introduced from Henan Province, 12 July 2022 (fl.), photos by Ren-Kun Li (http://ppbc.iplant.cn/tu/10960727). **Jinmen City: Zhongxiang City**, without precise locality, no date (fl.), *Zhongxiang Pubic Health Bureau s.n.* (Figure 5: B, C, H, I; HIB [0101695]). **Shiyan City:** Zhangwan, Huaguo, Taozigou, 700 m, 30 July 1974 (fl.), *Ren-Huang Huang 2940* (HIB [0101694]); **Fang County**, Guanyin Cave, 800 m, borde des chemins, 5 Aug. 1938 (fl.), *K.M. Liou 9041* (IBSC [0519223], PE [01023877], WUK [0056206]); Zhongba, Zhongba Public Health Clinic, 400 m, 21 Aug. 1974 (fl.), *Ren-Huang Huang 3079* (HIB [0101692]). **Wuhan City:** cultivated at the College of Life Science and Technology, Huazhong University of Science and Technology, 2 July 2020 (fl.), photos by Mao-Teng Li (http://ppbc.iplant.cn/tu/8644616).

I8. Hunan (introduced): Changsha City: Hunan Agricultural University, on green belts, 17 Sept. 2016 (fl.), photos by Bu-Yun Zhang (https://ppbc.iplant.cn/tu/3045467). **Zhangjiajie City**: Dayong Bridge Park, on gree belts, 16 Aug. 2017 (fl.), photos by Wei-Qiang Qin (https://ppbc.iplant.cn/tu/3761946).

I9. Jilin. **Baicheng City: Da'an City**, Heli, 45.5554°N, 123.4071°S, 139 m, meadow, 19 July 2015 (fl.), *Ying-Xin Huang 1092* (IFP [IFP0230269, IFP0230316, IFP0230360]); Ping'an, 45.9394°N, 123.8902°E, 150 m, 20 July 2015 (fl. & fr.), *Ying-Xin Huang 1241* (IFP [IFP0230786, IFP0230718]); **Taonan County**, southern county, 45.7634°N, 122.9187°S, 151 m, meadow, 17 July 2015 (fl.), *Ying-Xin Huang 208* (IFP [IFP0228216, IFP0228302]); Heishui Town, 45.268°N, 122.8197°E, 153 m, 15 July 2015 (fl.), *Ying-Xin Huang 532* (IFP [IFP0229100, IFP0229048, IFP0229151]); **Tongyu County**, the Forage Experiment Station, alkaline grassland, 23 Aug. 1959 (fr.), *Baicheng Exped. 325* (JLSLKY [JLSLKY02855], PE [01023508, 01023507]); Yongqing, 44.844°N, 123.059°E, 150 m, 15 July 2015 (fl.), *Ying-Xin Huang 500* (IFP [IFP0229073, IFP0229017, IFP0229124]); **Zhenlai County**, suburban, roadside and sand land, 15 Aug. 1959 (fl. & fr.), *Baicheng Exped.178* (JLSLKY [JLSLKY02856], PE [01023510, 01023511]). **Changchun City:** 1973 (fl. & fr.), *Pharmacy Department of 243 Army s.n.* (IBSC [0519275, 0519276]). **Siping City: Shuangliao City**, Zhengjiatun, Mt. Aobao, sand land, 4 June 1950 (fl.), *Pei-Yun Fu et al. 43* (PE [01023524]). **Songyuan City: Changling County**, south mountain of the Stallion farm, 150 m, meadow, 4 June 2015 (fl.), *Ying-Xin Huang 78* (IFP [IFP0227805, IFP0227903]); **Fuyu City,** Sanjingzi Town, 44.1693°N, 125. 3 669°S, 152 m, meadow, 28 July 2015 (fl. & fr.), *Ying-Xin Huang* 3374 (IFP [IFP0233687, IFP0233922, IFP0233806]); Fangpengzi, 45.1299°N, 125.8313°E,

185 m, 28 July 2015 (veg.), *Ying-Xin Huang 3419* (IFP [IFP0233849, IFP0233964, IFP0233730]); **Qian'an County**, Daxia, 44.0134°N, 124.2972°S, 144 m, meadow, 23 July 2015 (fl.), *Ying-Xin Huang* 1350 (IFP [IFP0230948, IFP0231017, IFP0231085]); **Qianguo County**, Chaganhua, near Harjin, 161 m, meadow, 25 July 2015 (fl.), *Ying-Xin Huang* 3217 (IFP [IFP0233005, IFP0233065, IFP0233125].

I10. Jiangsu. **Changzhou City: Liyang City (former Liyang County)** , 23 June 1963 (fl.), *Shou-Rong Zhang 509* (NAS [NAS00129974]). **Huai'an City: Huaiyin District (former Huaiyin County)**, Jichang, by the former Yellow River, 12 Sept. 1958 (fl.), *Fang-Xun Liu et al. 10996* (IBSC [0519227], NAS [NAS00129975], PE [01023763]); **Xuyi County**, Fengmoding, 13 Aug. 1961(fr.), *Xuyi Med. Exped. 011* (NAS [NAS00579502]). **Lianyungang City: Ganyu County**, Haitou Town, Kuangkou Village, coastal beach, 30 Oct. 2011 (veg.), *Bao-Cheng Wu et al. TT1247* (NAS [NAS00603206, NAS00603207]); **Guanyun County**, Sihe Township, Yeshan, southeast of the hillside, 29 July 1958 (fl. & fr.), *Anonymous 9553* (NAS [NAS00129958]); Shie-kou, climbing on wall, 26 Oct. 1932 (fr.), *Chang & Cheng 917* (IBSC [0519238], NAS [NAS00129987]); **Haizhou District**, "Hai-tcheou", 1 June 1924 (fl.), *P. Courtois 35639* (NAS [NAS00129980]); Ibid., 15 Aug. 1925 (fl.), *P. Courtois 36609* (NAS [NAS00129957]); Ibid., 1 Sept. 1925 (fr.), *P. Courtois 37007* (IBSC [0519236], NAS [NAS00129983]); Ibid., 16 Oct. 1926 (fr.), *P. Courtois 39508* (NAS [NAS00129976]); Ibid., 17 Oct. 1926 (fr.), *P. Courtois 31553* (NAS [NAS00129967]); Ibid., 5 June 1926 (fl.), *P. Courtois 39035* (NAS [NAS00129960]); "Hai chow", foot of south city wall, 5 July 1928, *Wei-Yi Yang 3241* (NAS [NAS00129991]); **Lianyun District**, "Xinhailian City", Dongshan, damp place, 17 Aug. 1958 (fl.), *Anonymous 20835* (HHBG [HZ048324], IBSC [0519233]). **Nanjing City:** Hiu-k'eou, 15 July 1931 (fl.), *Anonymous 2579* (NAS [NAS00129981]); Ibid., 8 June 1925, *P. Courtois 36553* (IBSC [0519237]); Ibid., 22 June 1925 (fl.), *P. Courtois 36706* (NAS [NAS00129968]); Ibid., 22 June 1925 (fl.), *P. Courtois 46705* (NAS [NAS00129972]); Ibid., 22 June 1925 (fl.), *P. Courtois 26707* (NAS [NAS00129969]); Near the top peak of the Zijin mountain, in the grass on the top of the mountain, 29 Aug. 1934 (fl.), *Xian-Yu Ho* 2698 (HIB [0101689], NAS [NAS00129962, NAS00129970]); Ming Tomb, 7 July 1934 (fl.), *C.C. Kung [Jia-Ji Gong] 141* (NAS [NAS00129999]); Purple Mountain Observatory, roadside, 12 Aug. 1935 (fl.), *Bin-Quan Chen 499* (NAS [NAS00129986]); Linggu Temple, 22 Oct. 1940 (veg.), *Anonymous 885* (NAS [NAS00129990]); Pede montis Tzuehin-shan (Purple Mountain), 22 Oct. 1940 (veg.), *H. Migo s.n.* (IBSC [0519284]); Zijin hill, 22 Oct. 2022 (veg.), photo by Jia-Hao Shen (Figure 4D); **Liuhe District (former Liuhe County)**, Sihe Township, Zhishan, southeast hillside, 29 July 1958 (fl. & fr.), *Xin-Lian Zhu 9553* (HHBG [HZ048330], HIMC [0026616], NAS [NAS00129958], WUK [0210025]). **Suzhou City:** Lion Park, 7 June 1955 (fl.), *Anonymous 2043* (PE [01023775]). **Suqian City (former Suqian County):** Gujushan, 15 Sept. 1958 (fl.), *Fang-Xun Liu 11079* (NAS [NAS00129984]). **Wuxi City: Yixing City**, Zhangzhu, near Zhuling Primary School, 100 m, piedmont, 25 Aug. 1960 (fl. & fr.), *Wen-Zhe Fang et al. 289* (HIB [0101690], KUN [0267391], LBG [00084709], NAS [NAS00129979], PE [01023762], WUK [0220279]). **Xuzhou City:** east of downtown, limestone mountain, 31 Aug. 1952 (fl.), *Xian-Pu Wang 20397* (PE [01552347]); **Feng County**, Yuezhuang, Dashahe Orchard, 1 Aug. 1958, *Fang-Xun Liu et al. 10334* (IBSC [0519229], NAS [NAS00129963]); **Jiawang District**, Dalu Mountain, grassland at the foot of the mountain, 9 Sept. 2008 (fr.), *Kang Ye et al. YZH-343* (NAS [NAS00597032]); **Pei County**, Fruit Forest Farm, farmland, 30 July 1958 (fl.), *Fang-Xun Liu 10323* (NAS [NAS00129985]); **Pizhou City**, Aishan, 60 m, grasses on hillside, 18 Aug. 2011 (fl.), *Qi-Xin Liu & Yu-Ning Xiong 3366* (NAS [NAS00582254, NAS00582255]); **Quanshan District**, Qiligou, orchard, 23 July 1958 (fl.), *Fang-Xun Liu 10223* (NAS [NAS00129961]); **Tongshan District (former Tongshan County)**, Zhengji, corrosion at the edge of a tomb, 18 July 1958 (fl.), *Nanjing Zhongshan*

Botanical Garden 20028 (HHBG [HZ048323, HZ048325, HZ048326, HZ048327, HZ048329, HZ048332], IBSC [0519231], WUK [0209305, 0209516]); Wanglou Bridge, riverside, 18 July 1958 (fl.), *Nanjing Zhongshan Botanical Garden 20028* (WUK [0209486]); Hanwang Township, 24 July 1958 (fl.), *Fang-Xun Liu et al. 10264* (HHBG [HZ048328], PE [01023764]); Heishan, grasses on the top of the mountain, 27 Aug. 2011 (fl.), *Qi-Xin Liu & Yu-Ning Xiong 3348* (NAS [NAS00582249, NAS00582250]); **Xinyi City (former Xinyi County)**, Maling, Wuhuading, 2 July 1974 (fl.), *Anonymous 74072* (NAS [NAS00129978]). **Yancheng City: Sheyang County**, Shuangzeshe, roadside and wasteland, 18 July 1958, *Anonymous 15159* (HHBG [HZ048331]); **Xiangshui County**, Yunhe Township, Nanxiaogang, farmland side, roadside, 27 Oct. 2011 (veg.), *Bao-Cheng Wu et al. TT1012* (NAS [NAS00603205]). **Yangzhou City: Baoying County**, Bayu Township, roadside, 31 Aug. 1958 (fl.), *Anonymous 16076* (NAS [NAS00129995]). **Zhenjiang City: Dantu District (former Dantu County)**, Mt. Wangfeng, 21 July 1958 (fl.), *Hao-Yuan Long 7376* (IBSC [0519232]); **Danyang City (former Danyang County)**, Chenshan, 3 Aug. 1958 (fl.), *Hao-Yuan Long* 7800 (IBSC [0519235, 0519224, 0519225]).

I11. Liaoning. **Anshan City: Xiuyan Manchu Autonomous County**, Gejialing, 19 Aug. 1928 (fl.), *Y. Sato 4480* (PE [01023370]); Tangchi, back hill of Toudao, in meadow, 9 Sept. 1959 (fr.), *Wei Wang et al 1493* (HNWP [76021], PE [01647793]); Qianyingzi Reservoir, 40.2108°N, 123.1749°E, 164 m, hill slope, 11 Aug. 2016 (fl.), *Wei Cao et al. 7409* (IFP [IFP0246231, IFP0246249, IFP0246311]). **Benxi City:** in a park, 6 July 1959 (fl.), *Wei Wang et al. 246* (PE [01023521]); Mt. Pinding , on dry mountain slope, 7 July 1959 (fl.), Wei Wang et al. 246 (PE [01023520]); **Benxi Manchu Autonomous County**, caohekou, no date (fl. & fr.), *Y. Yabe s.n.* (NAS [NAS00208831]); **Huanren Manchu Autonomous County**, Xiangyang Township, Huilong Electricity Station, 41.0936°N, 125.3005°E, 199 m, flood land of riverside, 26 Aug. 2015 (fr.), *Wei Cao et al. 6452* (IFP [IFP0244501]). **Chaoyang City: Harqin Left Wing Mongolian Autonomous County**, Nanshou Town, 41.584°N, 119.464°E, 563 m, 28 July 2015 (fl.), *Jing-Hua Yu 397* (IFP [IFP0258368]); Zhongsanjia Town, Guojiagou Village, 41.584°N, 119.871°E, 542 m, 16 July 2016 (fl. & fr.), *Jing-Hua Yu 788* (IFP [IFP0258783]); Gushanzi, 41°16′00.62″N, 119°55′18.76″E, 251 m, 21 June 2018 (veg.), *Xiao-Zhen Man B196* (SYAU [SYAUF011726]); **Jianping County:** Shaoguoyingzi to Mt. Erlong, 1 July 1959 (fl. & fr.), *Chong-Shu Wang et al. 3665* (IFP [11902008x0004, 11902008x0005], PE [01023514], WUK [0219083]);; Shahai, 41.584°N, 119.729°E, 309.5 m, 1 Aug. 2015 (fl. & fr.), *Jing-Hua Yu 629* (IFP [IFP0258931]); Xutaizi, 41° 25′ 12.69″ N, 119° 35′ 43.93″ E, 485 m, 21 June 2018 (veg.), *Xiao-Zhen Man B197* (SYAU [SYAUF011727]); **Lingyuan County:** Dawangzhangzi Township, front hill of Xiaowangzhangzi Village, 41.584°N, 119.241°E, 598 m, 15 July 2016 (fl.), *Jing-Hua Yu 304* (IFP [IFP0258297]); Miaoxi Village, Niuheliang Museum, 41.584°N, 119.313°E, 690 m, 31 July 2015 (fl.), *Jing-Hua Yu 847* (IFP [IFP0258823]). **Dalian City:** Lingshui, Lingshui Temple, 15 Aug. 1930 (fl.), *M. Kitagawa s.n.* (TI [TI00204080], holotype of *Cynanchum sibiricum* var. *latifolium*); without precise locality, *Ren-Jun Yang s.n.* (SYAU [SYAUF 006034]); **Ganjingzi District**, Mt. Paoya, 13 July 2010 (fl. & fr.), photos by Dong Wang (https://ppbc.iplant.cn/tu/650626); **Lvshunkou District (former Lvshun City)**, Baiyu hill, 120 m, hill slope, 13 Sept. 1951 (fr.), *Zhan Wang et al. 923* (IFP [11902008x0006, 11902008x0007], PE [01023803]); Taiyanggou, 30 June 1910 (fr.), *Y. Yabe s.n.* (NAS [NAS00208836]); ibid., Sept. 1909 (veg.), *Y. Yabe s.n.* (NAS [NAS00208838]); without precise locality, 29 July 1933 (fl.), *Y. Sato 4497* (PE [01023512]); **Wangfangdian City (former Fu County)**, Southern hill to Baoli Temple, sandy land of riverside, 10 July 1964 (fl.), *Chang-Qing Lin 755* (IFP [11902008a0001, 11902008a0002]). **Dandong City: Fengcheng City (former Fengcheng County)**, Mt. Fenghuang, 3 July 1957 (fl.), *You-Chang Zhu et al. 965* (GZAC [GZAC0020895], IFP [11902008a0005, 11902008a0006,

11902008a0007], KUN [0267400], NAS [NAS00208830]); Fenghuang Ancient City, 40°23′07″N, 124°05′11″E, 117 m, 14 July 2013 (fl.), *Zhong-Yu Li CaoW5114* (KUN [1375690]); Hujiabao, 40.5982°N, 124.2537°E, 80 m, 16 Aug. 2016 (fl.), *Wei Cao et al. 7570* (IFP [IFP0248658, IFP0248712, IFP0248765]). **Fuxin City: Zhangwu County**, Zhanggutai, no date (fl.), *Ren-Jun Yang B0408* (SYAU [SYAUF006032]) *& B0410* (SYAU [SYAUF006033]); Zhanggutai, 250 m, 4 Oct. 1952 (fr.), *T.N. Liou et al. 5484* (PE [01023515]); Haertao to Daqinggou, roadside, 18 June 1951 (fl.), *T.N. Liou et al. 2999* (IBSC [0519269], IFP [11902008a0003, 11902008a0004]), PE [01023516]. **Huluodao City:** without prciese locality, 29 Sept. 1958 (fr.), *Chong-Shu Wang et al. 3356* (IFP [11902008x0001, 11902008x0003, 11902008x0004, 11902008x0005, 11902008x0010]); **Jianchang City**, without precise locality, 8 Oct. 1982 (fr.), *Guang-Qing Guan B158* (SYAU [SYAUF006051]); **Lianshan District**, Tashan, Duanmucong, 40.911518°N, 120.895226°E, 7 m, hill top, 8 Aug. 2015 (fl.), *Gui-Juan Du et al. HLD-P001-09* (SYAU [SYAUF009444]) *& HLD-P001-10* (SYAU [SYAUF009445]) *& HLD-P001-11* (SYAU [SYAUF009446]); **Suizhong County**, 40°26.713′N, 120°15.375′E, 96 m, 28 July 2016 (fl.), *Yan-Bo Zhou & Yi-Nuo Wang SZ-P003-017* (SYAU [SYAUF010020, SYAUF010027, SYAUF010031]); Qiansuo, Yangtun, seaside and farmland side, 15 July 1959 (fl.), *Shu-Xin Li et al. 432* (PE [01023522]); **Xingcheng County**, Xigou, 40.417°N, 120.509°E, 33 m, 25 July 2019 (fl.), *Wei Cao 8581* (IFP [IFP0259079, IFP0259080]). **Jinzhou City: Beizhen City**, Fulao, Shifo, north of Xigou, 41.663647°N, 121.785896°E, 67.6 m, 28 Aug. 2015 (veg.), *Feng-Hong Ma et al. BZ-P001-08* (SYAU [SYAUF009423]); **Linghai City**, Wendilou, Tizigou, 41.243834°N, 121.046106°E, 125.2 m, 13 Aug. 2015 (fl.), *Long Bai B187* (SYAU [SYAUF012726]); **Yi County**, Dadingbao, back hill, 41.4428513°N, 121.048576°E, 4 Aug. 2015 (veg.), 224 m, *Jui-Juan Du et al. YX-P003-10* (SYAU [SYAUF009427]); Toudaohe, Zhuanchengzi, back hill, 41.485062°N, 121.076645°E, 166.4 m, 5 Aug. 2015 (veg.), *Jui-Juan Du et al. YX-P004-05* (SYAU [SYAUF009430, SYAUF009432]) *& YX-P004-07* (SYAU [SYAUF009428]) *& YX-P004-08* (SYAU [SYAUF009429]). **Liaoyang City: Liaoyang County**, Yong Ning, Aug. 1913 (fl.), *L. Chanet & J.H. Serre 397* (part of P [P03872653]); Pai-t'a, 14 Aug. 1930 (fr.), *L. Chanet & J.H. Serre 2998* (P [P03872653]). **Shenyang City:** South bank of Hunhe River, 13 June 1953 (fl.), *Chong-Shu Wang et al. 1122* (IBSC [0519268], IFP [1190208x0008, 1190208x0009], KUN [0267399], PE [01023519], WUK [0187347]); Without precise locality, 21 July 1982 (fl.), *Guang-Qing Guan B157* (SYAU [SYAUF006050]); Dongling, 25 Aug. 1909 (veg.), *Y.Yabe s.n.* (NAS [NAS00208837]); Beiling, 24 July 1933 (fl.), *Tokioi Suzuki 10042* (NAS [NAS00208829]); **Faku County**, Dongdaling, Hongtulizi, 42.482206°N, 123.462711°E, 206 m, *Long Bai B248* (SYAU [SYAUF012787]); **Kangping County**, Shandongtun, Wangping Village, 42.907911°N, 123.421844°E, 108.4 m, 23 July 2015 (fr.), *Long Bai B417* (SYAU [SYAUF012956]); **Xinmin City**, Zhoutuozi, Dantuozi, 42.156623°N, 122.6436298°E, 57-63 m, 10 May 2016 (veg.), *Bo Qu XM-P002-059* (SYAU [SYAUF002267]) *& XM-P002-060* (SYAU [SYAUF002271]) *& XM-P002-061* (SYAU [SYAUF002255]) *& XM-P002-062* (SYAU [SYAUF002278]) *& XM-P002-063* (SYAU [SYAUF002252]) *& XM-P002-065* (SYAU [SYAUF002257]); without precise locality, 29 July 1983 (fl.), *Zhen-Fu Fang & Qing-Li Wang 2840* (IFP [11902008a0008, 11902008a0009]). **Tieling City:** Tatientze Village, 250 m, on the slope, 11 July 1930 (fl.), *H.W.Kung 472* (PE [01023874]); Without precise locality, 2 Aug. 1910 (fl. & fr.), *Y. Yabe s.n.* (NAS [NAS00208835, NAS00208827]); Ibid., 18 June 1983 (fl.), *Dong-Chang Gao & Guang-Qing Guan B19* (SYAU [SYAUF006054]); **Changtu County**, Taizimiao, 42.857282°N, 124.19714°E, 250.1 m, 5 Aug. 2015 (fr.), *Long Bai B61* (SWAU [SYAUF012599]) *& B436* (SYAU [SYAUF012975]); Changtu Town, 1 Aug. 1910 (fl.), *Y.Yabe s.n.* (NAS [NAS00208828]); Ibid., 42.815156°N, 124.189066°E, 227.6 m, 26 July 2017 (fl. & fr.), *Long Bai B150* (SYAU

[SYAUF012689]). **Yingkou City: Bayuquan District**, Xiaodongtun, 40.3186095°N, 122.1613641°E, 68-74 m, 31 July 2015 (fl.), *Bo Qu YK-P001-133* (SYAU [SYAUF000928]); **Dashiqiao City**, 40°37.596′N, 122°28.092′E, 155 m, 25 July 2016 (fl.), *Yan-Bo Zhou & Yi-Nuo Wang DSQ-P001-007* (SYAU [SYAUF010010, SYAUF010011]); 40°39.423′N, 122°26.332′E, 148 m, 25 July 2016 (fl.), *Yan-Bo Zhou & Yi-Nuo Wang DSQ-P002-009* (SYAU [SYAUF010012, SYAUF010013]); **Gaizhou City**, 40°27.535′N, 122°30.388′E, 98 m, 26 July 2016 (veg.), *Yan-Bo Zhou & Yi-Nuo Wang GZ-P005-005* (SYAU [SYAUF010009]); Eastern downtown, 18 June 1959 (fl.), *Wei Wang et al. 6* (PE [01023517, 01023518]). **Unknown:** Gongzhuling, 14 Aug. 1918 (veg.), *Y.Yabe s.n.* (NAS [NAS00208834, NAS00208833], PE [01023643]); Qiaozi, 7 Aug. 19110 (fr.), *Y. Yabe s.n.* (NAS [NAS00208839]); *Pei-Yun Fu* 671 (IBSC [0519265]).

I12. Nei Mongol. **Alxa League: Alxa Left Banner**, Shuanghei Mt., piedmont plain, 4 Aug. 1958 (fl.), *PE Herbarium 0065* (PE [01023533, 01647794]), WUK [0346378]; Alashan Mts., 20 July 1984 (fl.), *S.Y. Hu 19482* (PE [02090917]); Bayan Hot Town, Kuletu ditch, 1 Sept. 1980 (veg.), *Yi-Zhi Zhao et al. 2541* (HIMC [0026614]); 10 km north to Bayan Hot Town, 39.127213°N, 105.65401°E, 1322 m, 30 July 2014 (fl.), *You-Sheng Chen et al. 141073* (PE [02034772]); Tengger Els Town, 37.7458814°N, 104.9476263°E, 1358 m, *Lei Zhang QTP-LJQ-1237-3004* (KUN [1476769]); **Alxa Right Banner**, Badain Jaran Town, No. 766 County Road, 39.1948207°N, 101.7063529°E, 1525.12 m, 31 July 2020 (fl.), *Lei Zhang et al. QTP-LJQ-CHNO029-3043* (KUN [1499658]); Longshou Mts., 26 Sept. 1986 (fr.), *Shu-Run Liu 509* (HIMC [0026610]); Ibid., 26 July 1982 (fl.), *Shan-Ting Lei et al. 273* (HIMC [0026612, 0026613]); Without precise locality, 1980 (fr.), *Grassland Station s.n.* (HIMC [0026611]). **Baotou City:** Dengkou, 2.5 km north Maoqilai, Xigoumen Valley, calcareous rock soil, 13 Sept. 1951(fr.), *Xue-Yu Hou 12530* (PE [01581141]); Ural Mts., Hondelen ditch, 13 Aug. 1991 (fr.), *Yi-Zhi Zhao 91-313* (HIMC [0026666, 026668]); **Darhan Muminggan United Banner**, Mongolia australis: Hutjertu Gol, Camp. VIII, 19 June 1927 (fl.), *D. Hummel 1147* (S [S-GH-2040]); 23 June 1959 (fr.), *Agr. & Animal Husbandry Coll. R-43* (HIMC [0026603]); Hongqi Farm, 1300 m, 28 July 1975 (fl.), *Yu-Quan Ma & Zhong-Quan Liu 47* (HIMC [026604]); Rui'an Ecological Base, 11 July 1999 (fl.), *Bio. & Envir. Eng. Depart. 1997 Class 1103* (HIMC [0026664]); Ibid., 12 July 1999 (fl.), *Bio. & Envir. Eng. Depart. 1997 Class 2117* (HIMC [0026665]); **Tumote Right Banner**, Jiufeng Mt., *Dong-Lai Liu 76* (PE [01023311]); Ula Mts., Qingshui ditch, 6 Aug. 1991 (fl.), *Yi-Zhi Zhao 073* (HIMC [0026667]). **Bayannur City:** Mongolia australis, prope Hoburin-nor [Khoburin-nor], inter Camp. XXII et XXIII, in steppo arenoso, 29 Aug. 1927 (veg.), *D. Hummel 1494* (S [S05-9612, S-GH-2041]); **Urat Front Banner**, side of Yellow River, 10 July 1960 (fl.), *Ulanqab Wild Pl. Exped. 2* (HIMC [0026630]); **Urat Middle/Back Banner**, former Urat Middle & Back Banners, 12 July 1976 (fl.), *Ri-Ji Shi s.n.* (HIMC [0026608]); **Urat Back Banner (former Qog Banner)**, Yinggot, 15 July 1978 (fr.), *Yong & Yi-Zhi Zhao s.n.* (HIMC [0026609]). **Chifeng City:** vicinity of the suburban forest farm, 23 Sept. 1962 (fr.), *Wei Wang et al. 3151* (IBSC [0519274], PE [01023547]); **Aohan Banner**, front of the Sheep Farm, gentle hill slope, 19 June 1962 (fl.), *Mengning Exped. 125* (PE [01532718]); **Ar Horqin Banner**, Bayan Mandu, 20 July 1984 (fr.), *Shu-Run Liu 2860* (HIMC [0026660]); **Baarin Leaf Banner**, Chagan Hadassa Sumu, 26 July 1959 (fl.), *Agr. & Animal Husbandry Coll. Wu-18* (HIMC [0026602]); **Baarin Right Banner**, near Mt. Dabannanshan, sand land, 16 July 1962 (fl.), *Mengning Exped. 1236* (PE [01532721, 01023529, 01532720]); vicinity of Chinchou, near Dairen, June 1930 (fl.), *P.H. Dorsett & W.J. Morse 5855* (P [P03872502]) *& 5856* (P [P03872503]); **Hexigten Banner**, Ulyasta Mt., 45.6672°N, 116.7851°E, 1048.5 m, 7 Aug. 2015 (fr.), *Guo-Jin Zhang XM430* (PE [02115369, 02115370, 02115371, 02311853]); 45.6672°N, 116.7851°E, 923-1115 m, 7 Aug. 2015 (fl.), *Hai-hua Hu XM213-2* (PE [02115368]) *& XM213-3* (PE [02311854]) *& XM213-4* (PE [02115367]); East of Dalinoer

Lake, 43.210°N, 117.286°E, 1276 m, 24 June 2018 (fl. & fr.), *Quan-Sheng Chen CF02-4004* (PE [02246806]); **Linxi County,** East Yingtaogou, 43°33′29.94″N, 118°02′11.19″E, 820 m, 27 May 2019 (fl.), *Yan Xue 150424190527016LY* (IATM [IATM0003672]); **Ningcheng County**, Mt. Nanshan, Niangniang Temple, 3 June 1962 (fl.), *Mengning Exped. 25* (PE [01532716]); **Wengniute Banner**, Xiaoyujiawopu, wasteland, 29 June 1962 (fl.), *Mengning Exped. 581* (PE [01532717, 01532719]). **Hohhot City:** near Mt. Daqingshan ("Tats'ingshan"), 19 July 1931 (fl. & fr.), *W.Y. Hsia 2647* (PE [01023583, 01023327], WUK [0012170]); Back garden of Inner Mongolia Universit, artificial turf, 27 July 2000 (veg.), *Li-Qing Zhao 128* (HIMC [0026627]); Holgol, 3 June 1961 (fl.), *Anonymous 153* (HIMC [0026640]); Wusutu, 2 June 1956 (fl.), *Anonymous s.n.* (HIMC [0026634]); **Horinger County**, [illegible], 1138 m, 18 July 2014 (fr.), *Zhi-Ping Zhang & Qin-Qin Li 20140718007* (NMTC [00000109]); **Tumd Left Banner**, Nanshuangshu, vicinity of Guoshunyingzi, 12 July1958 (fl. & fr.), *Guang-Wei Wu 25* (HIMC [0026615]); Ibid., June 1958 (fl.), *Anonymous s.n.* (HIMC [0027068]); 26 miles north of Hohhot, Forest Station in Baishitou Valley, Yin Shan Mountains, metasedimentary substrate, 22 July 1990 (fl. & fr.), *S. Boyd et al. 4752* (BRY [V0164313], NY [04185092], RSA [RSA0286741]); Sharqin Town, Chezhouying, 1202.5 m, 9 Sept. 2018 (fr.), *Bi Qu et al. 150121180909004LY* (IATM [IATM0001888]). **Hinggan League: Chenbarhu Banner**, Hadatu Team 10, 17 July 1975 (fl. & fr.), *Wasteland Exped. 126* (HIMC [0026593]); **Horqin Right Front Banner**, 2.5 km southwest of Mt. Arshan, gravel Land on sunning slope, 6 July 1963 (fl.), *Pei-Yun Fu et al. 2482* (PE [01023309]); back mountain of Haoren, 610 m, stony hillside, 28 Aug. 1982 (fr.), *Med. Invest. Exped.1085* (HIMC [HIMC0026583]); Qarsan, 21 July 1983 (fr.), *Med. Invest. Exped. 1389* (HIMC [0026633]); **Horqin Right Middle Banner**, Bayan Huxu, 45.0846°N, 121.5618°E, 260 m, 29 July 2018 (fl.), *Quan-Lai Zhou et al. 2-1029* (IFP [IFP0253984, IFP0253985, IFP0253986]); Tule Mod, 45.5567°N, 1121.1715°E, 522 m, 26 July 2018 (fl.), *Quan-Lai Zhou et al. 2-785* (IFP [IFP0253757, IFP0253758, IFP0253759]); **Hulunbuir City:** Vicinity of Hailar, 580 m, 8 June 1951 (fl.), *Zhan Wang et al. 566* (IBSC [0519267, 0519270], KUN [0267397, 0267398], NAS [NAS00208825], PE [01023525, 01023550], WUK [0187715, 0040570]); Hailar National Forest Park, 49.2081355°N, 119.7134820°E, 649 m, 9 June 2019 (fl.), *En-De Liu & Binbin Ren 9100* (KUN [1534361]); West Hill Park, 49°12′28.17″N, 119°43′06.19″E, 628 m, 26 June 2019 (fl.), *Chong-Ling Zhang 150702190626046LY* (IATM [IATM0004869]); Hailar, 49°12′28.17″N, 119°43′06.19″N, 628 m, 11 June 2019 (fl.), *Chong-Ling Zhang 044* (YAK [YAK0007170]); **Arun Banner**, Fuxing, 48° 9′ 37.1″ N, 123° 6′ 21.8″ E, 391 m, 3 June 2016 (fl.), *Chong-Ling Zhang 105* (YAK [YAK0006894]); **Morin Dawa Daur Autonomous Banner**, Erhe, 49°19′22.21″N, 125°11′28.9″E, 237.6 m, 9 July 2018 (fr.), *Li-Jing Wo 150722180709002LY* (IATM [IATM0002071]); **New Barag Left Banner**, Amugulang Town, 47.9599°N, 118.6682°E, 701 m, 15 July 2018 (fl.), *Quan-Lai Zhou et al. 1-889* (IFP [IFP0251031, IFP0251032, IFP0251033]); Cuogang Town, 49.277°N, 118.0829°E, 557 m, 10 July 2018 (fl.), *Quan-Lai Zhou et al. 1-478* (IFP [IFP0249902, IFP0249903, IFP0249904]); **Tuquan County**, Tuquan Town, 45.3876°N, 121.5965°E, 292 m, 28 July 2018 (fl. & fr.), *Quan-Lai Zhou et al. 2-1014* (IFP [IFP0251779, IFP0251780, IFP0251781]); Baoshi Town, 46.022°N, 121.3912°E, 526 m, 25 July 2018 (fl.), *Quan-Lai Zhou et al. 2-739* (IFP [IFP0254332, IFP0254333, IFP0254334]); **Ulanhot City**, Yileleeteuk, 46.1486°N, 122.0408°E, 335 m, 22 July 2018 (fl.), *Quan-Lai Zhou et al. 2-664* (IFP [IFP0252562, IFP0252563, IFP0252564]); **Yakeshi City**, Urqihan, North Mt., 700 m, stony hillside, 1 Aug. 1981 (fl.), *Hui-JunMeng Xing-646* (HIMC [0026586]); Muyuan, 758 m, 18 July 2018 (fl.), *Chong-Ling Zhang 606* (YAK [YAK0005560]). **Ordos City (former Ih Ju League):** around Ordos, 15 July 1923 (fl. & fr.), *Licent 7037* (TIE [00053527, 00053528]); Taomiotan, 1500 m, moist grass plot, 8 Aug. 1933 (fl.), *W.Y. Hsia 3801* (PE [01023807, 01023400]); "Mongolia occidentalis, Terra

Ordos, valle fl. Hoang-ho", 24 Jul. – 5 Aug. 1871 (fl.). *N.M. Przewalsky 298* (LE [LE01036690], lecotothype of *Vincetoxicum sibiricum* var. *australe*); isolectotypes: K [000872724], LE [LE01036688, LE01036689], P [P03872677]. **Dalat Banner**, 1 km south of the banner, lime-smoky soil, 26 Aug. 1951 (fl. & fr.), *Xue-Yu Hou 12617* (PE [01581132, 01581133]); Zandanzhao, 4 July 1963 (fl.), *Mengning Exped. 389* (HIMC [0026629]); **Ejin Horo Banner**, Dalhut District, grassland, 9 July 1956 (fl.), *Yellow River Exped. 7291* (PE [01023734, 01614043], WUK [0087972]); **Jungar Banner:** Shatan Village, near the Yellow River, sand land, 6 Sept. 1956 (fr.), *Xue-Zhong Lang 291* (PE [01023549, 01580168, 01580167]); Yangshitasaibuna Forest Farm, 25 July 1975 (fl.), *Nei Mongol. Exped. 81* (HIMC [HIMC0026596, HIMC0026597]); Mazha Village, 20 June 1963 (fl.), *Mengning Exped. 165* (HIMC [HIMC0026598]); **Otog Banner**, Hatu Temple 1250 m, 30 June 1976 (fl.), *Yu-Quan Ma & Zhong-Ling Liu 76-067* (HIMC [0026600]). **Uxin Banner**, Hujitu, 25 June 1960 (fl.), *Nei Mongol Univ. Practice Team 25* (HIMC [HIMC0026595]); Zhadazhan, 1300 m, 10 Aug. 1953 (fl. & fr.), *K.T. Fu 7231* (IBSC [0519258], PE [01023717], WUK [0058494]); Uxin Ju, 25 July 1972 (fl.), *Zhi-Jie Xu 85* (HIMC [0026599]); Ibid., 28 July 1973 (fl.), *Zhi-Jie Xu 150* (HIMC [0026635]). **Tongliao City (formr Jirem League):** Horqin District, Melhit Town, Sanjiazi village, 43.4215°N, 122.1046°E, 173.2 m, 8 July 2016 (fl. & fr.), *Yue Zhang et al. XM893* (IFP [IFP0239583, IFP0244629, IFP0239571]); **Hollingol City**, 46.276724°N, 120.850763°E, 559 m, 26 July 2017 (fl. & fr.), *Bao Nie et al. XM18376* (PE [02311800]) & *XM18390* (PE [02311813]) & *XM18388* (PE [02311818]) & *XM18080* (PE [02311836]); **Horqin Left Wing Middle Banner,** Jiamatu Town, Qianxin Aile village, 43°55′59.12″N, 123°05′11.19″E, 141 m, 11 July 2019 (fl.), *Xiao-Fang Wang 150521190711004LY* (IATM [IATM0003379]); Zhurihe, 204.20 m, 31 July 2015 (fl. & fr.), *Yue Zhang et al. XM642* (IFP [IFP0238806, IFP0238944, IFP0239010]); **Horqin Left Wing Rear Banner**, Zheliuke Left No. 5 Banner, Daqinggou, 280 m, sand land, 11 June 1980, *Zheliuke Exped. 21* (HIMC [HIMC0026573]); **Hure Banner**, Wujiazi, 42.5275°N, 121.4736°E, 301.4 m, 31 July 2015 (fl.), *Yue Zhang et al. XM692* (IFP [IFP0238852, IFP0238925, IFP0238992]); **Jarud Banner**, 6 Aug. 1984 (fl. & fr.), *Jin-Shan Wu 3714* (HIMC [0026661, 0026662]); **Naiman Banner**, Baogutu Desert Scenic Spot, 17 July 2016 (fl.), *Yue Zhang et al. XM1233* (IFP [IFP0240453, IFP0240616, IFP0240535]). **Ulanqab City: Fengzhen City,** Xinchengwan, 40°26′14.2″N, 113°11′43.7″E, 1183.6 m, 27 July 2018 (fl.), *Li-Zeng Pang 150981180727044LY* (IATM [IATM0000987]); **Qahar Right Wing Middle Banner**, Huanghuagou, 41°08′15″N, 112°31′57″E, 1879 m, 14 May 2018 (fl.), *Baoyinde Ligeer 150927180514048LY* (IATM [IATM0000522]); **Shangdu County,** Tianjia village, 9 Aug. 1941 (fl.), *Qahar Province Agriculture and Forestry Division 209* (WUK [0406016]); **Siziwang Banner**, 15 km south to the Banner, 1600 m, 17 July 1964 (fr.), *Mengning Exped. 225* (HIMC [0026601]); Ulaanhua, 5 July 1991 (fl.), *Yi-Zhi Zhao 1157* (HIMC [0026656]); Bijia Mt., 5 July 1991 (fl.), *Yi-Zhi Zhao 2225* (HIMC [0026658]) *& 3136* (HIMC [0026657]); Hujitu, Xindi, 41°28′57.8″N, 112°02′13″E, 1725 m, 3 Aug. 2018 (fl.), *Batu Bao 150929180803012LY* (IATM [IATM0001726]); **Zhuozi County**, Fushengzhuang, 6 July 1974 (fl.), *Yu-Quan Ma & Wu 168* (HIMC [0026606]). **Xilingol League: Abag Banner**, Xilian Banner, State-run Breeding Farm, Gunaimiao Winter Camp, gentle slope, 26 July 1952 (fl. & fr.), *Shi-Ying Li et al. 314* (PE [01023310, 01023809, 01552531, 01552532]); 43.898661°N, 115.361967°E, 1144 m, 31 July 2017 (fl. & fr.), *Bao Nie et al. XM20996* (PE [02311799]) & *XM20997* (PE [02311797]) & *XM20998* (PE [02311796]) & *XM20999* (PE [02311798]) & *XM21037* (PE [02311811]) & *XM21120* (PE [02311810]) & *XM21121* (PE [02311809]) & *XM21335* (PE [02311789]) & *XM21337* (PE [02311788]) & *XM21015* (PE [02311792]) & *XM21297* (PE [02311795]) & *XM21298* (PE [02311793]) & *XM2199* (PE [02311794]) & *XM21122* (PE [02311819, 02311820]) & *XM21039* (PE [02311822]) & *XM21038* (PE [02311862]); Naren Boragasu, 44.0361°N, 114.3428°E, 1180

m, 16 Aug. 2015 (fr.), *Bo-Han Jiao XM1812* (PE [02311855]); **Duolun County**, south of Dayingfeng, 42.2383°N, 116.8353°E, 26 Aug. 2016 (fr.), *Xin Li XM6992* (PE [02311843]) & *XM6995* (PE [02311844, 02311845]) & *XM7002* (PE [02311842]) & *XM7003* (PE [02311857]) & *XM7005* (PE [02311839]) & *XM7006* (PE [02311858]) & *XM7014* (PE [02311840]) & *XM7018* (PE [02311841]); Songshukeng, 42.5148°N, 116.6323°E, 24 Aug. 2016 (fr.), *Xin Li XM4371* (PE [02311830]) & *XM4373* (PE [02311831]); Langweiba Mt., 42.0719°N, 116.0089°E, 30 Aug. 2016 (fl.), *Xin Li XM10084* (PE [02311829]) & *XM10086* (PE [02311828]); **East Ujimqin Banner**, 44.914681°N, 116.106099°E, 871 m, 30 July 2017 (fl. & fr.), *Bao Nie et al. XM20056* (PE [02311833]) & *XM20057* (PE [02311832]) & *XM20058* (PE [02311834]); 46.5828°N, 117.8203°E, 799 m, 12 Aug. 2015 (fl.), *Bo-Han Jiao XM1178* (PE [02311856]); 45.9608°N, 116.2647°E, 773 m, 13 Aug. 2015 (fl. & fr.), *Bo-Han Jiao XM1419* (PE [02311823, 02311824]); **Erenhot City**, Olympic Park, 43°39′08.88″N, 111°57′38.44″E, 933 m, 2 July 2019 (fl.), *Jiang Chang 152501190702073LY* (IATM [IATM0003880]); **Plain and Bordered White Banner,** Ulanqab town, Shargayin village, 42°50′51.82″N, 114°51′42.27″E, 1179 m, 16 July 2019 (fl.), *Buhechaolu Wang 152529190716007LY* (IATM [IATM0003151]); **Plain Blue Banner:** 7 Sept. 1957 (veg.), *Yu-Shu Liu 43* (HIMC [0026581]); 42.523901°N, 115.99918°E, 1328.8 m, 21 July 2017 (fl.), *Bao Nie et al. XM14615* (PE [02311808]) & *XM14659* (PE [02311801]) & *XM14660* (PE [02311850]); 42.837715°N, 115.880896°E, 1291.42 m, 21 July 2017 (fl.), *Bao Nie et al. XM14699* (PE [02311814]) & *XM14700* (PE [02311827]); **Sonid Left Banner**, Mandalt Industrial Park, 43°49′49.76″N, 113°38′34.45″E, 1027 m, 9 Sept. 2019 (fr.), *Buteegq 152523190909050LY* (IATM [IATM0004159]); Ecological Garden of Mandalt, 43°51′08.16″N, 113°37′38.86″E, 1020 m, 9 Sept. 2019 (veg.), *Siqin Batter 152523190909021LY* (IATM [IATM0004160]); 43.729696N, 112.618353E, 901 m, 2 Aug. 2017 (fl. & fr.), *Bao Nie et al. XM22109* (PE [02311807]) & *XM22110* (PE [02311812]) & *XM22111* (PE [02311825]); 43.833599N, 113.817768E, 1140 m, 31 July 2017 (fl. & fr.), *Bao Nie et al. XM21605* (PE [02311821]); **Sonid Right Banner**, Wendur Temple, 28 June 1974 (fl. & fr.), *Zhong-Quan Liu 74-293* (HIMC [0026605]); **West Ujimqin Banner**, 44.43847°N, 117.036682°E, 1051.88 m, 23 July 2017 (fl.), *Bao Nie et al. XM16209* (PE [02311805]) & *XM16216* (PE [02311806]) & *XM16217* (PE [02311803]) & *XM16221* (PE [02311804]); **Xilinhot City**, Hushuo Branch of Baiyinxile Ranch, 1050 m, typical grassland, 23 July 1983 (fl.), *Wen-Sheng Yang 128* (HIMC [HIMC0026576, HIMC0026578]); Qianzi Mountain, shady slope, 28 July 1983, *Wen-Sheng Yang et al. 357* (HIMC [HIMC0026577]); Ximeng, Baiyinxile Ranch, sand land, 25 June 1979 (fl.), *Shu-Xun Liu 543* (HIMC [HIMC0026579]) ; Taolin Tara, Roadside, 10 Apr. 1964 (fl.), *Nasan et al. Tao55* (HIMC [HIMC0026580]); Grassland Research Station, Baiyinxile Livestock Farm near Xolinhot, low sand hills just N of the field stat., 26 July 1990 (fl. & fr.), *S. Boyd et al. 5122* (RSA [RSA0286738]); Huiteng Xile, 43°25′03.6″N, 116°05′21.63″E, 1459 m, 30 June 2019 (fl. & fr.), *Tian-Xi Sun 152502190630014LY* (IATM [IATM0004537]); Baiyin Xile Botanical Garden, 43°55′19.01″N, 116°04′01.53″E, 975 m, 8 Aug. 2019 (fl.), *Qing-Hua Bai 152502190808031LY* (IATM [IATM0004538]); 43.932189°N, 115.789436°E, 1142 m, 31 July 2017 (fl. & fr.), *Bao Nie et al. XM20675* (PE [02311817]) & *XM20760* (PE [02311787]) & *XM20761* (PE [02311791]) & *XM20762* (PE [02311790]) & *XM20781* (PE [02311802]) & *XM20676* (PE [02311815]) & *XM20779* (PE [02311816]) & *XM20780* (PE [02311826]); 44.349295°N, 115.930751°E, 919 m, 30 July 2017 (fl. & fr.), *Bao Nie et al. XM20354* (PE [02311835]); 9 June 2018 (fl.), photo by Xin-Xin Zhu (Figures 3A, 4C: g, h; http://ppbc.iplant.cn/tu/4693104, http://ppbc.iplant.cn/tu/4693118); **without precise locality:** 9 Aug. 1958 (fl.), *Anonymous 23* (HIMC [HIMC0026575]). **Unknown:** Mongolia australis, Mont. Muni ula, 1871 (fl.), *N.M. Przewalski s.n.* (K [K000872725]); Mongolia australis, gobi, 1871 (fl.), *N.M. Przewalski s.n.* (E [E00780575]); Mongolia australis: prov. Sui-yüan,

Khonin-tsaghan-chölo-gol (Camp. Norinii XVI), in dry river bed, 22 July 1927 (fl.), *K. Georg Söderbom 6686* (S [S-GH-2025]); Mongolia australis, inter Camp. XI et XII, prope pag. Tso hwan tai, in valle fluminis, 3 Aug.1927 (fl. & fr.), *D. Hummel 1336* (S [S-GH-2040]); Wula Shan, 26 July 1957 (fl.), *Xue-Yu How 212* (RSA [RSA0286734]); Mandjourie, Kingham, 500 m, 6 July 1896 (fl.), *Chaffanjon 1423* (P [P03872535]); Mandjourie, Kingham, 500 m, 7 July 1896 (fl.), *Chaffanjon 1423* (P [P03872699]); Mongolie orientale, 1869-1874 (fl. & fr.), *A. David 1806* (P [P03872688]); Mongolia australis, gobi, 1871 (fl.). *Przewalski s.n.* (P [P03872687]); no date (fl.), *Yi-Zhi Zhao s.n.* (HIMC [0026651, 0026652]); Xilingol League (?), no date (fl.), *Teachers College s.n.* (HIMC [0026631]); Hunshandake Sandy Land, 16 Aug. 2007 (fl. & fr.), *2005 Class No. 6 Team 0506114* (HIMC [0038374]), *2005 Class No. 1 Team 01133* (HIMC [0038375]), *2005 Class No. 3 Team 170* (HIMC [0038376]).

I13. Ningxia. Shizuishan City: Pingluo County, "Taole County", Taole Farm, 16 Sept. 1952 (fr.), *Zhen-Wan Zhang 489* (NAS [NAS00208842], PE [01023741]). **Wuzhong City (former Wuzhong County):** Sunjiatan, 1220 m, sandy grassland, 24 Aug. 1981(fl. & fr.), *Yang-Peng Xu & Zhao-Ying Yu 1947* (PE [01647802], WUK [0433036, 0449364]); **Qingtongxia City:** Shaogang Town, 38.1207082°N, 105.8329521°E, 1235 m, desert steppe, 31 July 2019 (fr.), *Lei Zhang QTP-LJQ-1238-3028* (KUN [1476840, 1476841]); **Tongxin County**, Mt. Luoshan, photos seen from Li et al. (2017: 216); **Yanchi County**, Huamachi Town, Tongjiquan Village, 37°42′5″N, 107°20′18″E, 1390 m, woodland, 30 June 2017 (fl.), *Zhi-Bin He et al. HHZA0249* (KUN [1456385]); Aug. 1989, *Yu-Jiu Li & Duan-Zheng Lu 198908* (BJFC [00017467, 00017468, 00017469, 00017470]); Yanchi Grassland Station, 1500 m, sandy wasteland, 19 July 1977 (fl.), *Zhi-Ying Zhang & Hong-Jie Wang 94* (WUK [0414060]); near Gaoshawo,1600 m, semifixed dunes, 17 June 1877 (fl.), *Yi-E Chen et al. 62* (WUK [0414114, 0322552]). **Yinchuan City:** July 1961, *Ningxia University s.n.* (HIMC [0026623]); Western Suburbs, 1100 m, in the middle of sandy fields, 19 Sept. 1980 (fr.), *Zhao-Ying Yu 973* (WUK [0449366]); **Helan County**, Jinshan, South of Summer Camp, 1150 m, alluvial fan and roadside, 12 Aug. 1980, *Zhao-Ying Yu 184* (WUK [0449368]); south of the Helan County Grassland Station, 1150 m, alluvial fan, 14 Aug. 1980 (fl. & fr.) , *Zhao-Ying Yu 303* (WUK [0449367]); Helan Mountain Pangou, 1500 m, inside the valley, 25 May 1959 (fl.), *Ye-Qi He 2479* (WUK [0388845, 0156964]); Helan Mts, Xiaokouzi, 1500 m, in the valley, 24 May 1959, *Ye-Qi He 2411* (PE [01023732], WUK [0388690]); Helan Mts, Suyukou, 1700 m, hillside, 22 Aug. 1959 (fl. & fr.), *Ye-Qi He 7256* (WUK [0156211]]); Helan Mts, 2 Aug. 1983 (fl. & fr.), *Anonymous 5043* (HIMC [0026680]); Ho Lan Shn Mountains, 1375-2400 m, 10-25 May 1923 (fl.), *R.C. Ching 195* (PE [01023785]); **Lingwu County**, Baitan, 1030 m, sandy beach, 25 Aug. 1959 (fl.), *Ye-Qi He 6658* (PE [01023733]). **Yongning County**, Helan Mts. Reserve, on the way from Yinchuan to Azuoqi, 38°21′19.15″N,105°52′10.85″E, 1429 m, open gobi by the road, 17 July 2014 (fl. & fr.), *Jie Cai et al. 14CS9322* (KUN [1375682]). **Zhongwei City: Haiyuan County**, Pailou Mountain, hillside, 9 July 1956 (fl.), *Yellow River Exped. 5311* (PE [01023740, 01614045], WUK [0085765]); Xi'anzhou, 1800 m, ditch slope, 15 Aug. 1976 (fl.), *Li-Hua Zhou 2929* (HNWP [62897]); **Shapotou District**, sand land, 11 June 1956 (fl.), *Ming-Gang Li et al. 656* (IFP [11902008w0001]).

I14. Qinghai. **Haidong City: Ledu District ("Luotu hsien")**, Kaomiao, 2000 m, sandy district, 23 Jul. 1930 (fl.), *K.S. Hao [Hopkingson]* 736 (P [P03872698], PE [01023748], WUK [0012165]); **Minhe County**, between Yangjiadian and Gaomiao, 1190 m, fontes des rochens, 12 July 1936 (fl. & fr.), *K.M. Liou 5846* (WUK [0067295]); **Ping'an District (former Ping'an County)**, Ping'an Town, 1969 (fl.), *Zhen-Chang Zuo s.n.* (HNWP [0297342]); **Xunhua Salar Autonomous County:** Mengda, 1850 m, 11 Aug. 1973 (fl.), *Yu-Zeng Zhang 161* (HNWP [35783], NAS [NAS00208873]); Ibid., 2000 m, roadside, 25 June

1981 (fl.), *Ben-zhao Guo & Wei-Yi Wang 25381* (HNWP [98853]); Ibid., 2200 m, 6 June 1981 (fl.), *Ben-zhao Guo & Wei-Yi Wang 25141* (HNWP [98906]); Ibid., Qianpinggou, 2200 m, 25 June 1987 (fl.), *Ben-zhao Guo & Wei-Yi Wang 25362* (HNWP [98834]). **Hainan Tibetan Autonomous Prefecture: Gonghe County**, Laping, 2700 m, June 1965 (fl. & fr.), *Zhen-Duo Wei 9* (WUK [0296553]); **Guide County**, bank of Yellow River, 2200 m, dry hillside, 23 July 1989 (fl.), *T.N. Ho 1123* (HNWP [153571]); Pontoon Bridge, along the Yellow River, 2220 m, 2 July 1957 (fl.), *Anonymous 2217* (PE [01023749]); **Guinan County**, Bacang Farm, 10 July 1970 (fl. & fr.), *Anonymous s.n.* (HNWP [77215]); **Tongde County**, Lajiaxiang on N side of Hunghe, 34°41′N, 100°41′E, 3100–3200 m, on steep crumbling mudstone slopes, some rocky outcrops near river, 21 July 1993 (fl.), *T.N. Ho et al. 96* (PE [01023848]); **Xinghai County**, Heka, Yangqu, 2700 m, semidesert grassland, 23 June 1965 (fl.), *T.N. Ho 060* (HNWP [11992], NAS [NAS00208874], WUK [0290251]); Tangnaihai, 2800 m, river beach, 15 July 1963 (fl.), *Abandoned Farmland Exped. 279* (HNWP [136786]); Heka, Yangqu [erroneously recorded as Daheba, Qinggen Bridge], 2900 m [erroneously as 3900–4000 m], grassy hillside, 11 Aug. 1964 (fl. & fr.), *T.P. Wang 20020* (HNWP [136785]). **Xining City:** 2250 m, ditchside, 26 June 1958 (fl.), *P.C. Tsoong 8158* (HNWP [0100303], PE [01023728, 01023358], WUK [0100303]); Huangshui Forest Farm, 2350 m, hillside, 12 Aug. 2010 (fl.), *Qiang-Huang Zhang 10-131* (HNWP [HNWP00000474]); Chengbei District, Mt. Balang, 2462 m, 14 July 2022 (fl.), photo by Shun-Bang Zhao (Figure 4A; http://ppbc.iplant.cn/tu/10981604). **Huangnan Tibetan Autonomous Prefecture: Jianzha County**, Cambra, Shangluobu, 2135 m, in terrace, 29 June 1070 (fl.), *Li-Hua Zhou & Li-Nan Sun 845* (HNWP [0214925]); **Jianzha County**, vicinity of downtown, 2080 m, river beach, 23 June 1970 (fl.), *Shang-Wu Liu & Da-Shang Luo 1046* (HNWP [26000]); **Jainca County**, without precise locality, 25 June 1972 (fl.), *Ben-Zhao Guo 10024* (HNWP [32134]); **Tongren County**, Longwu River, 2600 m, river beach, 31 May 1972 (fl.), *Ben-Zhao Guo 10199* (HNWP [32332, 241410]).

I15. Shandong. **Binzhou City: Zhanhua District (former Zhanhua County)**, 9 Sept. 1955, *Jing Zhou 212* (PE [01023858]); **Zouping City,** Mt. Heban, 36.765597°N, 117.722907°E, 278.0 m, 1 Aug. 2018 (fl.), *Jian-Lin Lan* 201808001 (QFNU [QFNU0047558]); Mt. Huixian, 36.848658°N, 117.675784°E, 454.0 m, 23 Apr. 2018 (fl.), *Jian-Lin Lan 201804035* (QFNU [QFNU0047559]). **Dongying City: Guangrao County:** 5 Aug. 1959 (fl. & fr.), *Tai-Yan Zhou et al. 5648* (NAS [NAS00208885]); **Hekou District:** Xianeh Town, NE Erhe, 37°57′15.35″N, 118°41′29.28″E, 5.0 m, 16 July 2019 (fl.), *Yuan-Tong Hou et al. 370503190716137LY-1* (QFNU [QFNU0051442]) *& 370503190716137LY-2* (QFNU [QFNU0051443]) *& 370503190716137LY-3* (QFNU [QFNU0051444]) *& 370503190716137LY-4* (QFNU [QFNU0051445]); Yihe Town, west of Chengnjiuwuzi, 37°57′17.11″N, 118°35′32.23″E, 3.0 m, 16 July 2019 (fl.), *Yuan-Tong Hou et al. 370503190716131LY* (QFNU [QFNU0051441]); Gudao Farm, No. 3 division, 21 July 1959 (fl.), *Tai-Yan Zhou et al.* 5496 (NAS [NAS00208888]). **Heze City: Dongming County**, Aug. 1961 (fl.), *Xiao Fang 454* (HENU [0170210]). **Jinan City:** eastern suburbs, Quanfuzhuang, 6 June 1951 (fl.), *Anonymous K7360* (PE [01023679]); **Changqing District**, Wande Town, Yuhang, 200 m, 6 July 2005 (fl.), *Cheng-Yong Guo 055180-10* (PE [01647806]); from Geshi Town, Wande Town to Yanling Mt., 150 m, 17 July 2005 (fl.), *Cheng-Yong Guo 055242-1* (PE [01647815]); **Laiwu District**, East of the Main Teaching Building of Laiwu Vocational and Technical College (https://ppbc.iplant.cn/tu/462416). **Jining City: Gunzhou District (former Gunzhou City)**, Shaoling Park, 35°32′32.28″N, 116°46′37.21″E, 60 m, 9 July 2011 (fl.), *Cheng-Yong Guo 201101030* (GXMG [GXMG0071411], QFNU [QFNU0038199]); **Jinxiang Coutny:** Donggou River, south side of Chunji Bridge, 35.015637°N, 116.357882°E, 30.20 m, 26 Sept. 2016 (fr.), *Yuan-Tong Hou et al. 201609131-1* (QFNU [QFNU0029091]) *& 201609131-2* (QFNU [QFNU0029092]); Old Wanfu River, 29.6 m, 16

Oct. 2016 (fr.), *Yuan-Tong Hou et al. 201610149-1* (QFNU [QFNU0029093]) *& 201610149-2* (QFNU [QFNU0029094]) *& 201610149-3* (QFNU [QFNU0029095]) *& 201610149-4* (QFNU [QFNU0029096]) *& 201610149-5* (QFNU [QFNU0029097]); **Qufu City**, Jiuxianshan Hill, SE of Longweizhuang, 300 m, 19 Aug. 2004 (fl.), *Cheng-Yong Guo 1504105-1* (PE [02108870]); Kongzi Lake, SE of Zhangma River, 35.45773390°N, 117.19056760°E, 135.79 m, waterside, 20 July 2017 (fl.), *Cheng-Yong Guo et al. 20170720247-1* (QFNU [QFNU0031845]) *& 20170720247-2* (QFNU [QFNU0031846]) *& 20170720247-4* (QFNU [QFNU0031848]) *& 20170720247-5* (QFNU [QFNU0031849]); Ibid., 35.45754560°N, 117.19038460°E, 125.22 m, waterside, 20 July 2017 (fl.), *Cheng-Yong Guo et al. 20170720250-1* (QFNU [QFNU0031857]) *& 20170720250-2* (QFNU [QFNU0031858]) *& 20170720250-3* (QFNU [QFNU0031859]); Shimen Mt., 35°46′30.15″N, 117°06′01.64″E, 146 m, 5 June 2011 (fl.),*Yuan-Tong Hou 11624* (GXMG [GXMG0071318], QFNU [QFNU0009641]); Dayihe River, 35°34′37.40″N, 116°58′45.55″E, 59 m, 30 June 2011 (fl.), *Yuan-Tong Hou11822* (GXMG [GXMG0071425], QFNU [QFNU0009644]); **Sishui County**, Xingcun Town, Beichennan, 150 m, 11 Aug. 2002 (fl.), *Cheng-Yong Guo 15044-2* (PE [01647811]); Xingcun Town, 150 m, 4 July 2005 (fl.), *Cheng-Yong Guo 054211-6* (PE [01647812]); Jinzhuang Town, Xiyu Village, Red Hill, 35.569672°N, 117.220818°E, 235.7 m, 13 Sept. 2013 (fl. & fr.), *Cheng-Yong Guo et al. 13010466* (QFNU [QFNU0009635, QFNU0009636, QFNU0009637, QFNU0009638]); Quanlin Town, Qinglongshan Village, Mt. Qinglong, 35.666770°N, 117.531403°E, 247.7 m, 30 June 2013 (fl.), *Yuan-Tong Hou et al. 13010143* (QFNU [QFNU0009645, QFNU0009646]); **Weishan County**, Liangcheng Town, Dushanxi Village, vicinity of Chaoyang Cave, 35.101007°N, 116.750250°E, 78.9 m, 8 June 2014 (fl.), *Yuan-Tong Hou et al. 140144* (QFNU [QFNU0014998, QFNU0014999, QFNU0015000, QFNU0015001, QFNU0015002]); Northeast side of the Xue River Grand Bridge, 34.785606°N, 117.158857°E, 32.9 m, 12 July 2014 (fl.), *Yuan-Tong Hou et al. 140355* (QFNU [QFNU0014990, QFNU0014991]); Liangcheng Town, Grand Dingzi Mts., 35°09′24.747″N, 116°45′08.936″E, 115.39 m, 14 Aug. 2014 (fl.), *Yuan-Tong Hou et al. 3708261408140029LY* (QFNU [QFNU0014992, QFNU0014993, QFNU0014994, QFNU0014995, QFNU0014996, QFNU0014997]); Liangcheng Town, Dushan Isalnd, top of hill in the northwest, 35°06′07.428″N, 116°44′44.665″E, 58.16 m, 20 Sept. 2015 (fr.), *Kang-Man Wang et al. 370826150920341LY* (QFNU [QFNU0020353, QFNU0020354, QFNU0020355, QFNU0020356, QFNU0020357]); **Zoucheng City (former Zou County)**, Tianhuang Town, Huangshan Hill, 300 m, 15 July 2005 (fl.), *Cheng-Yong Guo 052226-6* (PE [01647804]); Ibid., 150 m, 15 July 2005 (fl.), *Cheng-Yong Guo 052252-1* (PE [01647813]); Mt. Yishan, 300 m, 5 Sept. 2004 (fr.), *Cheng-Yong Guo 1506133-1* (PE [01456934]); Tianhuang Town, Chaoyangsi Village, Fenghuang Hill, 35.395535°N, 117.330234°E, 262.55 m, 4 June 2016 (fl.), *Cheng-Yong Guo et al. 160111* (QFNU [QFNU0020341, QFNU0020342, QFNU0020343, QFNU0020344, QFNU0020345]); Tianhuang Town, E side of Fenghuang Hill, 35.389715°N, 117.338758°E, 350.8 m, 20 July 2012 (fl. & fr.), *Yuan-Tong Hou & Cheng-Yonng Guo 12010247* (QFNU [QFNU0038200, QFNU0038202], SDFGR [SDF1009283, SDF1009285]) *& 12010247-A* (QFNU [QFNU0001478, QFNU0001479, QFNU0001480]) *& 12010247-B* (QFNU [QFNU0006871, QFNU0006872]); Tianhuang Town, Liuzhuang Village, N side of Fenghuang Hill, 35.388210°N, 117. 337943°E, 382 m, 23 Sept. 2012 (fr.), *Yuan-Tong Hou et al. 12010662* (QFNU [QFNU0001475, QFNU0001476, QFNU0001477, QFNU0038201], SDFGR [SDF1009284]); Tianhuang Town, Sanxian Mts, foot of Fuxian Hill, 35.425877°N, 117.286648°E, 199.44 m, 5 June 2016 (fl.), *Cheng-Yong Guo et al. 160203* (QFNU [QFNU0020346, QFNU0020347, QFNU0020348, QFNU0020349, QFNU0020350, QFNU0020351, QFNU0020352]); Yishan Town, back hills of the Yishan Scenic Area, 35.329153°N, 117.016062°E, 154.2 m, 12 July

2012 (fl.), *Yuan-Tong Hou et al. 12010051* (QFNU [QFNU0001481, QFNU0001482, QFNU0001483, QFNU0038203], SDFGR [SDF1009279]); Tianhuang Town, Shibatang, 35.44591510°N, 117.33844230°E, 189.70 m, 29 May 2017 (fl.), *Yuan-Tong Hou et al. 20170529495-1* (QFNU [QFNU0038204]) & *20170529495-2* (QFNU [QFNU0038205]) & *20170529495-3* (QFNU [QFNU0038206]) & *20170529495-4* (QFNU [QFNU0038207]); Mt. Yishan, 8 Aug. 1959 (fl.), *Tai-Yan Zhou et al. 0006* (NAS [NAS00208891]). **Linyi City: Feixian County**, Tashan Hill, 11 July 2006 (fl.), 350 m, *Cheng-Yong Guo 20064-277-10* (PE [01647808]); Datianzhuang Town, Niulan, 300 m, 9 Sep 2006 (fr.), *Cheng-Yong Guo 20064-370-4* (PE [02108869]); **Mengyin County**, Mengshan, Water Curtain Cave, by farmland, 21 July 1959 (fl.), *Tai-Yan Zhou 6082* (NAS [NAS00208887], PE [01647796]); Yedian Town, Yanmazhuang, 250 m, 8 Sept. 2006 (fr.), *Cheng-Yong Guo 20063-363-3* (PE [01883549]) & *20063-363-4* (PE [01883549]); Liancheng Town, 300 m, 9 July 2006 (fl.), *Cheng-Yong Guo 20063-266-1* (PE [00869658]); Meng Mts., 14 Aug. 1959 (fl.), *Tai-Yan Zhou et al. 1147* (NAS [NAS00208893]); **Pingyi County**, Wutai Town, 300 m, 12 July 2006 (fl.), *Cheng-Yong Guo 20062-296-7* (PE [00869660]); Baotai Town, Dongyu, 250 m, 7 Sept. 2006 (fr.), *Cheng-Yong Guo 20062-388-7* (PE [01894365]); Vicinity of Pingyi Railway Station, 35°30′20.43″N, 117°38′27.07″E, 149 m, 18 June 2011 (fl), *Yuan-Tong Hou 11686* (GXMG [GXMG0071391], QFNU [QFNU0009642]); Meng Mts., Beishanjian, 12 July 2016 (fl.), *Bio. Sc. Class No. 4 Team 201604031* (QFNU [QFNU0020339]); Ibid., 12 July 2016 (fl.), *Bio. Sc. Class No. 10 Team 201610040* (QFNU [QFNU0020338]); Ibid., 12 July 2016 (fl.), *Bio. Sc. Class No. 6 Team 201606042* (QFNU [QFNU0020340]); Ibid., 12 July 2016 (fl.), *Hui-Ying Yu et al. 160712030* (QFNU [QFNU0020336, QFNU0020337]); **Yishui County**, Panchi Township, Shenjiadong, Langwo, 36°06′22.96″N, 118°33′04.61″E, 349 m, 22 Aug. 2013 (fl.), *Yuan-Tong Hou 12282* (QFNU [QFNU0009647]). **Qingdao City** ("Tsingtao"): Li Chuan, along ditches, 28 Jul. 1930 (fl.), *C.Y. Chiao 2878* (E [E00780684], IBK [IBK00097291], IBSC [0519262], NAS [NAS00208898], PE [01023857, 01023794]); no date (fl.), *Y. Yabe s.n.* (NAS [NAS00208897]); **Jiaozhou City**, recd. 15 Oct. 1904 (fl.), *H. Green s.n.* (IBSC [0519239]); **Laoshan District**, Vicinity of Mt. Laoshan Forestry Farm, 280 m, 13 June 1959 (fl.), *Anonymous 1295* (NAS [NAS00208894]); **Pingdu City**, campus of Pingdu No.1 Middle School (https://ppbc.iplant.cn/tu/988120); campus of Pingdu No. 1 Middle School, 9 July 2010 (fl.), photos by Gui-Xi Liu (https://ppbc.iplant.cn/tu/988120). **Rizhao City: Lanshan District**, Jufeng, Yeji Temple, 11 Aug. 1959 (fl.), *Tai-Yan Zhou10* (NAS [NAS00208890]). **Tai'an City: Feicheng City (former Fei Hsien)**, Mengshan, 250 m, on slope, 11 July 1936 (fl.), *T.Y. Cheo & L. Yen 16* (P [P03872700]); **Tai'an City, Ningyang County:** Geshi Town, 150 m, 2 July 2005 (fl.), *Cheng-Yong Guo 051207-6* (PE [01647814]); **Taishan District**, Mt. Taishan, Jingshiyu, 29 Aug. 1925 (veg.), *Y.Yabe s.n.* (NAS [NAS00208882]); Mt. Taishan, Chaoyang Cave, 18 Aug. 1951, *Shandong Univ. 21* (NAS [NAS00208886], PE [01023856]); Mt. Taishan, East gate, East Heilaopo Hill, 7 Sept. 1959, *Shandong Wild Pl. Exped. /Tai-Yan Zhou 7016* (NAS [NAS00208889], PE [01023673]); **Xintai City**, Shilai Town, Baimasi Hill, 300 m, 13 Aug. 2004 (fl.), *Cheng-Yong Guo 1505122-1* (PE [02108873]); Shilai Town, Liujiashan, 300 m, hillside wasteland, 26 June 2006 (fl.), *Cheng-Yong Guo 20061-298-7* (PE [00869659]) *& 20061-298 -10* (NY [04185091]); Ibid., 250 m, 12 July 2006 (fl. & fr.), *Cheng-Yong Guo 20061-628-1* (PE [02108860]); Ibid., Liujiashan, 250 m, on slope, 13 July 2006 (fl.), *Cheng-Yong Guo 20061-315* (NY [04185090]); Ibid., 250 m, hillside wasteland, 8 Sept. 2006 (fr.), *Cheng-Yong Guo 20061-417-1* (NY [02729999]); Ibid., 35°45′8.25′N, 117°25′48.5″E, 210 m, 9 Aug. 2011 (fl.), *Cheng-Yong Guo 201102024* (GXMG [GXMG0071350]). **Weifang City: Changle County**, Tangbu, 23 Aug. 1959 (f.), *Tai-Yan Zhou et al. 5363* (NAS [NAS00208896]); **Gaomi City (former Gaomi County)**, Shuangyu, 14 Aug. 1959 (fl.), *Tai-Yan Zhou et al. 2374* (NAS [NAS00208892]); **Linqu County**, Yishan Mts., 36°11′05.05″N, 118°35′13.35″E, 471 m, side

of farmland, 1 June 2011 (fl.), *Yuan-Tong Hou 11557* (GXMG [GXMG0071328], QFNU [QFNU0009639]); **Qingzhou City**, "Ching Chou fu,", I tu hsien [Yidu], recd. 1895 (fl.), *S. Couling 41* (E [E00780677]). **Weihai City: Huancui District**, Tiger Hill, 37.23°N, 122.10°E, 103.0 m, 9 July 2020 (fl.), *Qi-Ke Yu 201841534754* (QFNU [QFNU0059856]); Zhengqi Mt., Zhuyujie, 31 May 1959 (fl.), Tai-Yan Zhou et al. 2020 (NAS [NAS00208895]). **Yantai City:** "Tchéfou dunes", [1860] (fl.), *O. Debeaux 79* (P [P03872669], ex herb. O. Debeaux, lectotype of *Vincetoxicum sibiricum* f. *linearifolium*, Figure 2C); "Tchéfou dunes", [1860] (fr.), *O. Debeaux 79* (P [P00877371], ex herb. O. Debeaux, possible original material of *Vincetoxicum sibiricum* f. *linearifolium* (P [P00877371]); Tchéfou, dunes, 9 Oct. 1860 (fr.), *O. Debeaux s.n.* (P [P03872649], possible original material of *Vincetoxicum sibiricum* f. *linearifolium*); Tchéfou, dunes, 1860 (fl.), *O. Debeaux s.n.* (P [P00877376], possible original material of *Vincetoxicum sibiricum* f. *linearifolium*); Tche'fou, dunes maritimes a Yan-tai, 4 Sept. 1860 (fr.), *O. Debeaux 91* (P [P00877375], possible original material of *Vincetoxicum sibiricum* f. *linearifolium*); Tche'fou, dunes maritimes a Yan-tai, 1860 (fl.), *O. Debeaux 91* (P [P00877374], possible original material of *Vincetoxicum sibiricum* f. *linearifolium*); Tche'fou – granules Dunes de la baie de Ten-cheou-fou – Yan-tai, 9 Oct. 1860 (fl. & fr.), *O. Debeaux 79* (P [P03872679], possible original material of *Vincetoxicum sibiricum* f. *linearifolium*); Tche'fou – granules Dunes de la baie de Ten-cheou-fou – Yan-tai, 14 July 1860 (fl.), *O. Debeaux 79* (P [P00877373], syntype of *Vincetoxicum sibiricum* f. *linearifolium*); Tche'fou, Dunes, 23 Aug. 1860 (fr.), *O. Debeaux s.n.* (P [P03872692], syntype of *Vincetoxicum sibiricum* f. *linearifolium*); Tche'fou, Dunes, 14 July 1860 (fl.), *O. Debeaux s.n.* (P [P00877370], syntype of *Vincetoxicum sibiricum* f. *linearifolium*); Che'fou, sables à Yan-tai, bord de la me [Sands at Yan-tai, at the edge of the sea], 23 Aug. 1860 (fr.), *O. Debeaux 103* (P [P03872678], possible original material of *Vincetoxicum sibiricum* f. *linearifolium*); Che'fou, sables à Yan-tai, bord de la me [Sands at Yan-tai, at the edge of the sea], 12 July 1860 (fl.), *O. Debeaux 103* (P [P00877372], possible original material of *Vincetoxicum sibiricum* f. *linearifolium*); Che-foo, no date (fl. & fr.), *M. Fauvel s.n.* (P [P03872691]); Chefoo, SE hills, 30 m, 6 Oct. 1933 (fr.), *K.M. Liou 1803* (PE [01023677, 01023678]); 37°33′34.81″N, 120°32′5.33″E, 245.0 m, 25 July 2020 (fl.), W*ei-Qing Sun 202007077* (QFNU [QFNU0059855]); **Laiyang City**, Wandi Town, Fushui Village, 11 June 2013 (fl.), photos by Zhi-Xue Lv (https://ppbc.iplant.cn/tu/1366423); **Muping District (former Muping County)**, Kunyu Mts, 300 m, grassy hillside, 29 Oct. 1957 (veg.), *K.M. Liou 720* (PE [01478387]); Vicinity of Mt. Kunyu Forestry Farm, roadside, 14 July 1956 (fl.), *PE Herb. 3182* (PE [01023852]); Dianhou Village, 80 m, 19 Aug. 1933 (fl.), *T.N. Liou & K.M. Liou 1205* (PE [01023675, 01023675, 01023855]); Longquan Town, Nanguan, 37°19′18.32″N, 121°46′48.54″E, 44 m, 7 Aug. 2011 (fl.), *Yuan-Tong Hou 12114* (GXMG [GXMG0071339], QFNU [QFNU0009640]); Longquan Town, 37°18′18.37″N, 121°46′21.37″E, 82 m, 9 Aug. 2011 (fl.), *Yuan-Tong Hou 12192* (GXMG [GXMG0071435], QFNU [QFNU0009643]); **Qixia City**, Mt. Yashan, Dazhegou, 1 June 1959 (fl.), Tai-Yan Zhou 4157 (NAS [NAS00208884], PE [01023674]). **Zaozhuang City: Shanting District**, Sangcun Town, Dongluoshan Village, 39.96°N, 116.40°E, 136.0 m, 10 July 2020 (fl.), *Li Zhang 27* (QFNU [QFNU0059853]); **Shizhong District,** Mt. Zhuoshan, hillside, 24 May 2009 (fl.), photos by Ming-Xiao Sun (https://ppbc.iplant.cn/tu/235992); **Tengzhou City**, Jiehe Town, Longshan, 300 m, 28 Aug. 2005 (fl.), *Cheng-Yong Guo 053277-8* (PE [01647807]). **Unknown:** 1869 (fl.), recd. *Alt Willamson* (E [E00780680] upper left); *Anonymous 331* (PE [01023680]); *Anonymous 457* (PE [01023681]); Aug. 1950 (fl.), *Zi-Xiao Zhao 16039* (PE [01023766]).

I16. Shaanxi. **Ankang City: Xunyang City (former Xunyang County),** Qili, hillside farmland, 30 June 1970 (fl.), *Shaanxi Herbal Med. Exped. 1577* (WUK [0284915], XBGH [XBGH012272]); Ganxi Town, Zhangping, Yangjiayuanzi, 20 Aug. 1959 (fl.), *Pei-Yuan Li*

8884 (KUN [0267394, 0267395], WUK [0137227, 0356081]); Miaoling, Shizhaipo, 15 Aug. 1959 (fl.), *Pei-Yuan Li 8838* (KUN [0267393], WUK [0356555, 0137215]). **Baoji City: Chencang District,** vicinity of Hudian Station, hillside, 820 m, along the railway line, 3 Aug. 1960 (fl. & fr.), *K.T. Fu 13830* (WUK [0161472, 0362537]); **Feng County:** Tang Zang, 1240 m, on the rocky beach, 2 July 1960 (fl.), *K.T. Fu 12951* (WUK [0162554, 0362833]); **Qianyang County**, Donghegou, 700 m, hillside, 24 Aug. 1974 (fl. & fr.), *Zhao-Ying Yu 98* (WUK [0301459, 0301460]); **Mei County**, Taibai Mts., Heihu Pass, 5 July 1937 (fl.), T.*N. Liou & P.C. Tsoong 1927* (WUK [0020658]); **Taibai County**, West Taibai Mts., Baiyun Gorge, 1050 m, river beach in valley, 5 July 1958 (fl.), *Zhi-Ping Wei 1131* (WUK [0106296]). **Hanzhong City: Foping County**, Shimudi Township, 500 m, hillside farmland, 25 June 1998 (fl.), *Wei-Qing Li 90* (NAS [NAS00209909]); **Lueyang County**, Baishuijiang, Maliutanggou, 720 m, roadside, 15 Aug. 1978 (fl.), *Zhi-Ying Zhang 18030* (WUK [0365843, 0365844]); North Pass, 530 m, farmland, 14 Oct. 1958 (veg.), *Chang-Lin Tang 702* (WUK [0104521]); **Mian County**, "Mianyang", 600 m, roadside, 23 Aug. 1956 (fl.), *K.T. Fu 5527* (IBK [IBK00097319], IBSC [0519277], PE [01023714, 01023778], WUK [0060229]); south bank of Hanhuichu head, 20 July 1942 (fl.), *K.T. Fu 3798* (NAS [NAS00208864], PE [01023343, 01023709], WUK [0020678, 0359 014]); **Ningqiang County**, Yangping Pass, 1200 m, hillside, 23 Sept. 1958 (veg.), *Ying-Lin Qiao 136* (WUK [0165822]); **Xixiang County**, Yankou Town, 500 m, hillside, flat wasteland, 15 June 1999 (fl.), *Fu-Gui Li 334* (NAS [NAS00603227, NAS00603228]); **Yang County**, from downtown to Xiecun Bridge, 550 m, on the road, 5 Aug. 1952 (fl.), *K.T. Fu 5327* (IBK [IBK00097318], IBSC [0517934], PE [01023712]). **Shangluo City: Danfeng County**, Longwangmiao, 950 m, by farmland of hillside, 19 June 1964 (fl.), *Jin-Xiang Yang & Yi-Min Liang 3033* (WUK [0228421]); **Shanyang County**, Manchuan, Xiaohekou, 7 July 1960 (fl.), *No. 4 Internship Team 0275* (HNWP [71614]); **Shangnan County**, Jiangxigou to Baiyu, 400 m, by farmland, 9 Oct. 1958 (veg.), *Ben-Zhao Guo 4222* (WUK [0112649]); **Shangzhou District (former Shang County)**, Heishan Street, 1100 m, hillside wasteland, 12 Aug. 1952 (fl.), *T.P. Wang 16096* (HIB [0101413], PE [01023713], WUK [0062032]); Sanchahe, Laowu, 1000 m, by the hillside road, 14 Aug. 2013 (fl.), *Si-Feng Li et al. 17885* (XBGH [XBGH009100, XBGH014142]); **Zhen'an County**, Zheshi, Mt. Jinlong, 850 m, by the ditch, 10 Aug. 1958 (fl.), *Zhi-Ping Wei 1675* (HNWP [91045], WUK [0107475, 0403801]). **Weinan City: Huayin City (former Huayin County)**, Hua Mts., West Xianyukou, 480 m, valley mouth, roadside, 7 Aug. 1973 (fl. & fr.), *K.T. Fu 16960* (WUK [0064135, 0363352]). **Xi'an City: Huyi District (former Hu County)**, Tze-Wu-Chin, Hua-Yuan-Tze, side of field, 28 Aug. 1934 (fl. & fr.), *Y.Y. Pai 1152* (PE [01023706, 01023744]); Lao-Yu-Kow, on slope, 7 Sept. 1934 (fr.), *Y.Y. Pai 1245* (PE [01023704]); **Lantian County**, Wangchuan, roadside on mountain slope, 31 Aug. 1972 (fl.), *Ping Ke 358* (WUK [0293101]); **Zhouzhi County**, Ma-Ying, field side,on the slope, 14 Sept. 1934 (fl.), *Y.Y. Pai 1335* (PE [01023705, 01023743]). **Xianyang City: Jingyang County**, near Jinghui Farm, 15 Sept. 1954 (fl.), *Anonymous 96* (PE [01567352, 01567353]); **Qian County**, Qianling, 880 m, in forest, 9 Aug. 2010 (fl.), *Ce-Ming Tan & Gui-hua Yi 10589* (JJF [JJF00026780, JJF00026781]); **Weicheng District**, Zhouling Town, Chou Ling, 5 Sept. 1934 (fl.), *Fenzel 2367* (WUK [0072668]); **Yangling District**, vicinity of Yangling Town, July 1958 (fl.), *620 Commando s.n.* (WUK [0291899]); Vicinity of Wukung, 1937 (fl.), *S.T. Wang s.n.* (WUK [0079765]). **Yan'an City: Huanglong County**, Huanglong Mts, Shibao Town, Yehu Village, on road bank, 11 Aug. 1939 (fl.), *K.T. Fu 3194* (PE [01023708], WUK [0055122]); Hongshiya, 900 m, in grasses of hillside, 21 June 1985 (fl.), *Jin-Xiang Yang & Suo-Lin Huang 6299* (WUK [0468126, 0468127]); **Luochuan County**, Huaibai Town, in forest, 18 July 2016 (fl.), *Rong Yang YR20160718-2* (BNU [0027334]); **Wuqi County**, Zhengfugou, 100-1500 m, 25 July 2008 (fl.), *Jian Zhao 114* (BJFC [BJFC00058512]); **Zhidan County**, Yongning, 20 July 1974 (fl.), farmland,

Tian-Min Pan 270 (WUK [0304730]); **Zichang City (former Zichang County)**, Lejiaping, 990 m, 6 Sept. 1953 (fr.), *K.T. Fu 7632* (IBSC [0519257], PE [01023715], WUK [0058491]); Sangshuping Village, roadside, 26 July 2016 (fl.), *Rong Yang YR20160726-14* (BNU [0027333]). **Yulin City: Dingbian County**, near the Great Wall in the west of the city, on the sandy soil beside the road, 1000 m, 28 July 1972 (fl.), *Zhi-Ying Zhang 17544* (WUK [0291468, 0364562]); **Hengshan District (former Hengshan County)**, Dianji Temple, dry farmland of hillside, 20 July 1956 (fl.), *Yellow River Exped. 7490* (PE [01023718], WUK [0088279]); **Jingbian County**, Zhangqugou, sunny place at hill foot, 4 Aug. 1956 (fl. & fr.), *Yellow River Exped. 7654* (PE [01023700, 01614044], WUK [0086841]); 2.5 km south from Zhangjiapan, roadside, 26 Aug. 1953 (fl.), *Y.W. Tsui 10516* (PE [01023344, 01023865]); Dongkeng, 3 Aug. 2016 (fl. & fr.), *Rong Yang YR20160803-1* (BNU [0027335]); **Mizhi County**, Dujiashi Town, Chuanbao, dry farmland, 9 June 1956, *Yellow River Exped. 6584* (PE [01023742, 01614046], WUK [0087268]); **Shenmu City (former Shenmu County)**, south of suburbs, sand dunes, 7 July 1960 (fl.), *Wen-Ding Zhang 86* (PE [01023863], WUK [0166086]); **Suide County**, Jiuyuangou, 1150 m, hillside, 12 Aug. 1957 (fl.), *Zhen-Wan Zhang 660* (WUK [0089341]); **Yuyang District**, Southwest of Shahekou, 1160 m, sand dune, 6 July 1952 (fl.), *K.M. Liou 11534* (PE [01023711] , WUK [0067479]); **Yuyang District**, Mahe, Denglaxia,1460 m, grassland, 27 June 1956 (fl.), *Yellow River Exped. 7133* (PE [01023719, 01614048]); Mt. Qingyun, 1040 m, sand dune and shady slope, 5 Aug. 1953 (fl.), *Shaanxi–Gansu Exped. [Y.W. Tsui] 10389* (CDBI [CDBI0117812], KUN [0267365], PE [01023342, 01023866]). **Unknown:** Northern Shaanxi, 31 July 1922 (fl.), *P. Licent 6846* (PE [01023780], TIE [00051150]); Tai Kung Ling, 3 July 1934 (fl.), *Fenzel 2292* (WUK [0072666]); Tai Kung Ling, 30 July 1934 (fl.), *Fenzel 2283* (WUK [0072669]).

I17. Shanxi. **Datong City:** Tatung-fu, N. Montagnes, 18 July 1914 (fl.), *R.P. Licent 193* (P [P03872515], TIE [00026561]); near Tatung, 1433 m, sandy flat, 6 Aug. 1934 (fl. & fr .), *Tso-Pin Wang 2303* (PE [01023656, 01023699]). **Changzhi City: Qinyuan County**, Baizi, Xiguan River Beach, roadside, 20 June 1959, *Ke-Jian Guan & Yi-Lin Chen 731* (HNWP [136807], HSIB [HSIB012386]], PE [01023328, 01023669], WUK [0325834]). **Jincheng City:** "Tsincheng hsien", Taihangshan, 850 m, pres des champs, 16 July 1937 (fl.), *K.M.Liou 7279* (PE [01023703], WUK [0020670]); **Lingchuan County**, Gujiao, Niupigou, 22 July 1959 (fl.), hillside grassland, *Shi-Ying Bao & Sheng-Jun Yan 359* (PE [01023331, 01023849]). **Jinzhong City: Lingshi County**, by the water field, 4 July 1954 (fl.), *Shanxi Exped. 081* (PE [01553379]). **Linfen City:** without precise locality, 25 Sept. 2015 (fl.), *2014 Traditional Chinese Medicine Class FJ002106* (SXTCM [SXTCM0016296]). **Lvliang City: Lishi District (former Lishan County)**, downtown, Wangjiagou, Sangqingkou, 1 July 1955 (fl.), *Yellow River Exped.1549* (PE [01614040, 01614041]); **Lin County,** on the way from Lin County to Gancaogou, hillside, 11 Aug. 1955 (fl.), *Yellow River Exped. 1788* (PE [01023662, 01614047], WUK [0077429]); 5 km north to Sanjiao Town, 1000 m, on the sunny side of the hill, 14 June 1955 (fl.), *Yellow River Exped. 1396* (PE [01023661, 01614042]); **Liulin County**, Wangjiagou, 2000 m, 26 July 1957 (fl.), *Y.W. Tsui 10319* (HIB [0101696]); **Shilou County**, Sijiang Village, sunny mountain slope, 12 June 1955 (fl.), *Yellow River Exped. 1587* (PE [01614053, 01023660], WUK [0077440]); **Xing County**, Caijiaya, mountain slope, 25 Aug. 1955 (fl . & fr.), *Yellow River Exped. 2152* (PE [01023851, 01614038, 01023871], WUK [0077449]); Ibid., mountain slope, 22 Aug. 1955 (fl.), *Yellow River Exped. 2085* (PE [01023663, 01614039], WUK [0077450]). **Taiyuan City:** on the way from Wuliyuan Temple to Dongliu Temple, 21 Aug. 1932 (fl.), *Anonymous 2928* (PE [01023657]); neighbourhood outside Shanxi University, 28 June 1973 (fl.), *Ben-Liang Li s.n.* (SXU [SD00015367, SD00015368]); Mt. Tianlong, 37.713159°N, 112.427553°E, 958 m, 3 June 2018 (fl.), *Dong-Mei Kong k1138* (SXU [00026534]); Mt. Tianlong, 29 Sept. 2016 (fl.), *Kai-Li Cui s.n.* (SXU [00024370]). **Xinzhou City: Fanzhi**

County, Xiyankou, 39°17′54.78″N, 113°34′34″E, 1140 m, by the farmland, 20 July 2014 (fl.), *Chang-Hong Duan Dch0415* (PE [02014761]); Anlezhuang, 39°20′12.87″N, 113°40′18″E, 1273 m, 6 July 2014 (fl.), *Chang-Hong Duan Dch0197* (PE [02034119, 02034120]); **Hequ County**, Quta Village, 750 m, at the hillside, 6 Sept. 1955 (fr.), *Yellow River Exped. 3102* (PE [01023659, 01614052, 01614035], WUK [0077427]); **Ningwu County**, Damiao Village, 2000 m, in the shade place of grassland, 27 July 1957 (fl.), *K.M. Liou et al. 1918* (PE [01023666, 01478384]); **Pianguan County**, on the way from downtown to Lougou Port, 1000–1300 m, at the sunny side of the hill, 27 Aug. 1955 (fr.), *Yellow River Exped. 3017* (PE [01023664, 01614037] , WUK [0077434]); **Wutai County**, Menxianshi, Houjiazhuang village, farmland, 2 Jul. 1959 (fl.), *Ke-Jian Guan & Yi-Lin Chen 2055* (HNWP [136808], HSIB [HSIB012385], PE [01023330, 01023671]); Gengzhen, 14 July 1959 (fl.), 1800 m, *Ke-Jian Guan & Yi-Lin Chen 2347* (HNWP [136803], HSIB [HSIB012388], PE [01023329, 01023670]); Gengzhen, 16 Aug. 2015 (veg.), *Xiao-Lu Wang s.n.* (SXU [00021256, 00021257, 00021260]). **Yuncheng City:** Zhongtiao Mts., 11 July 1954 (fl.), *Shanxi Exped. 158* (PE [01553380]); **Wanrong County**, Jiacun, 35° 21′ N, 110° 37′ E, 560 m, 31 July 2020 (fl.), *Yan-Jing Feng 201941325977* (QFNU [QFNU0059852]) & *02* (QFNU [QFNU0059854]). **Unknown:** "Chansi N", 17 July 1914 (fl.), *P. Licent 7386* (PE [01023790]); Vallee Vouy Ho, 800 m (Province du Chansi), *Legendre 120* (P [P03872655]); "W. Shensi", 3000-4000 ft, open filed-side, 25 July 1929 (fl. & fr.), *T. Tang 1206* (IBSC [0519261], NAS [NAS00208826, NAS00208850], PE [01023781, 01023782]).

I18. Sichuan (new record). Guangyuan City, Eastern hill, 600 m, *Guangyuan Exped. 6001* (SM [SM716700642]).

I19. Tianjin. **Jizhou District:** vicinity of downtown, 92 m, roadside 29 Aug. 1952 (fl.), *T.N. Liou et al. 4496* (IFP [11902008r0002, 11902008r0003, 11902008r0004], PE [01023568]); near downtown, 92 m, roadside, 29 Aug. 1952 (fl.), *Shen-Xuan Liu et al.* 4496 (IFP [11902008r0001, 11902008r0002, 11902008r0003, 11902008r0004], PE [01023568]); Xiaying, Changzhou, 250 m, at the foot of the mountain, 11 July 1976 (fl.), *Jia-Yi Liu Ji0323* (TIE [00026778]); Xiaying, 8 July 1976 (fl.), *Jia-Yi Liu s.n.* (TIE [00026777]); Baitan, Qingshan Ridge, 14 Sept. 1975 (fr.), *Cai-Ling Wang s.n.* (TIE [00026771]); Xiaying, Huangyaguan, 8 July 1976 (fl.), *Jia-Yi Liu & Cai-Ling Wang 0263* (TIE [00026775]); [locality illegible], 29 July 1982 (fl.), *Cai-Ling Wang s.n.* (TIE [00026776]); Qingguang Medicinal Materials Factory, 3 Aug. 1978 (fl.), *Rui Chen & Jin Xue 0731* (TIE [00026779]); Beigou, 5 Sept. 1975 (fr.), *Rui Chen & Cai-Ling Wang s.n.* (TIE [00026774]); Baitan, Qingshanling, 10 Sept. 1975 (fr.), *Ying Yan et al. s.n.* (TIE [00026773]); Baitan, *Ying Yan &Wang-Lei Wang s.n.* (TIE [00026772]). **Xiqing District:** Yangliuqing, 3 May 2007 (veg.), photos by Chi-Yi Zan (https://ppbc.iplant.cn/tu/184933). **Unknown:** Tientsin, 26 July 1912 (fl.), *Mary Strong Clemens 1919a* (E [E00780689]); Tientsin, 1912 (fr.), *Mary Strong Clemens 1918b* (E [E00780675]); Tientsin, Leichwang, 23 Aug. 1912 (fl.), *Mary Strong Clemens 1918* (E [E00780674]).

I20. Xinjiang. **Alashankou City:** Alashankou to Tuori, at 509km of Provincial Highway S318, 45°33′47.16″N, 82°34′25.70″E, 1468 m, stony hillside, 15 June 2015 (fl.), *Cheng Liu & Ji-Dong Ya 15CS10295* (KUN [1375677]). **Altay Region: Burqin County,** 120 km north of Burqin County, Hailuo Beach, wasteland at the bottom of arid valley, 14 Sept. 1956 (fr.), *R.C. Ching* 3105 (PE [01023753, 01023754]); 450 m, 25 Sept. 1997 (veg.), *Duan-Zheng Lu 199709* (BJFC [00017455, 00017456, 00017457]); **Habahe County**, Habahe Foresty farm, in gobi, 10 Sept. 1964 (fr.), *Ge-Lin Zhu et al. 6689* (NAS [NAS00208872], PE [01023752], WUK [0232769]); **Qinghe County**, Altay Mts., Altay Valley, 2000 m, 13 Aug. 1956 (fr.), *R.C. Ching/ Xinjiang Exped. 1541* (IBSC [0519264], KUN [0267402, 0267403]); Chagan River Basin, 2000 m, arid land, 8 or 9 Aug. 1956, *R.C. Qing 1541* (PE [01023755, 01023758]); Southwestern mountain slope to the Altay refuse landfill, 47°48′9″N, 88°5′11″E,

950 m, *Ji-Pei Yue & Hong-Liang Chen YC*-XZ028 (KUN [1478254, 1478262]). **Tacheng Region: Bukser Mongolia Autonomous County**, the Fourth District of Hamusti Kaziersai, sunny mountain slope, 1400 m, 3 July 1959 (fl.), *An-Ren Li & Jia-Nan Zhu /Xinjiang Exped. 10846* (PE [01023750, 01023751, 01023757]); 202 km of 318 Provincial Highway, 49° 39′47″N, 85°38′31″E, 1344 m, in mountain meadow, 10 July 2017 (fl. & fr.), *Yi He & Yu Zhou BNU2017XJ192* (BNU [0039518, 0039519, 0039550, 0039551]); In the Scrub Grassland Community, 24 Sept. 2000 (fr.), *Yi-Zhi Zhao 139* (HIMC [HIMC0038469]); **Tuoli County**, about 245 ㎞ on the S221 Highway from Tuoli to Kuitun, 45°24′28.35″N, 84°14′11.08″E, 1118 m, stony dry hillside, 11 Sept. 2016 (fr.), *Ji-Dong Ya et al. 16CS13879* (KUN [1445155, 1462755]). **Urumqi City:** 850 m, July 1957 (fr.), *Bayi Agricultural College 22475* (WUK [0348267]); Tianshan Kemqi, 10 July 1955 (fr.), *Bayi Agricultural College 1770* (PE [01023771]).

I21. Without precise localities: *Fung-Yuan Hwang 553* (WUK [0050865]); *Fung-Yuan Hwang 561* (IBSC [0519281], PE [01023818]); *K.T. Fu 82* (WUK [0050867]); *Anonymous 40229* (IBSC [0519282]); Northeast China, *Liaoning Tieling Agricultural School 42* (PE [01023802]); Northeast China, 21 Aug. 1925 (fr.), *J. Kozlov 673* (TIE [00058990, 00058991, 00058992]); 10 Sept. 1917 (fr.), *P. Licent 5867* (PE [01023776]); 12 June 1924 (fl.), *P. Licent 7386* (PE [01023792]); 16 July 1927 (fr.), *P. Licent 8265* (PE [01023791]); 15 July 1923 (fl. & fr.), *P. Licent 7037* (PE [01023788]); 14 Aug. 1930 (fl. & fr.), *P. Licent 9852* (PE [01023779, 01023789]); *Xue-Yi Hou 12503* (PE [01023774]); *Ki-Mon Liou 4340* (PE [01023773]); *Anonymous 444* (PE [01023784]); “Benchang” , 1 May 1906 [or 1917?], *Anonymous 28* (PE [01023783]); 1956 (fl.), *Yellow River Exped. 1077* (PE [01023745]); *T.P. Wang 2850* (PE [01023623]); *Kun-Tsun Fu 82* (PE [01023819]); Jehol, in Gobi, 12 June 1924 (fl.), *Licent 7386* (TIE [00069615]); Mongol Camp, Sunliuliang, 7 Sept. 1984 (fr.), *Yun-Bin Zhang 2102* (NEFI [089001007004001]); *Anonymous 1547* (PE [01478385]); 18 June 1962 (fl.), *Chen & Kong 50* (PE [01532859]); *Anonymous 21117* (PE [01566446]); Eastern China, 1910 (fl.), *I.B. Balfour 130* (E [E00780685 & E00780686]); Eastern China, 1910 (fl. & fr.), *I.B. Balfour s.n.* (E [E00780676]); trias-touen, prefecture de tchao-tchao, 10 Aug. 1905 (fl.), *abbé Clauch 71* (E [E00780682]); Sib. China, 1866 (fl.), plantes env. Par l’Academie de S. Tetersbout. G, ex Herb. Maille, in Herb. Th. Delacour, *Anonymous s.n.* (E [E00780680 right plant]); P [P04968892]); Yang chia ping, July 1905 (fl.), *A.K. Schindle 25* (E [E00780672]); “fl. a Ch.”, no date (fl.), *Bunge s.n.* (E [E00780570]); China borealis, 1831 (fl.), *Bunge s.n.* (P [P03872673, P03872674 left plant]); China, no date (fl.), ex Herb. Petrop., *Anonymous s.n.* (L [L.2723145]); China borealis, Mongholia, ex Herb. Bunge, 1831 (fl.), *Bunge s.n.* (P [P03872681]); 500 km env au sud. de Pékin, 1903–1935 (fl.), *L. Chanet & J.H. Serre s.n.* (P [P03872651]); Montagnes de Ping yun sse, 12 June 1912 (fl.), *Anonymous 86* (P [P03872648]); Chine septentrionale, 1863 (fl.), *Simon 2* (P [P03872690]); Chine septentrionale, 1863 (fl.), *Simon 69* (P [P03872689]); Chine septentrionale, 1863 (fl. & fr.), *Simon 32* (P [P03872650, P03872670, P03872654]); no date (fl. & fr.), *Anonymous s.n.* (WUK [0020669]); no date (fl.), *Anonymous s.n.* (PE [01023747, 01023793, 01023801]); no date (fl.), *Anonymous 167* (PE [01023800]); no date (fl. & fr.), *Anonymous s.n.* (PE [01023772]); Northeast China, 21 Aug. 1925 (fr.), *J. Kozlov 673* (TIE [00058990, 00058991, 00058992]).

II. KAZAKHSTAN. East Kazakhstan: North-east part of the Zaisan depression, environs of the village Kizil-Tas, along road on road metal soil, 29 June 1997 (fr.), *Isa O. Baituli et al. s.n.* (P [P03903169]); Songaria chin. ad lacum Saisang-Nor. [Lake Zaysan], 1840 (fl.), *Anonymous s.n.* (E [E00780574, E00780572, E00780568], P [P04464925, P03872504]); Katon-Karagai district, Katon-Karagai State, R. Bukhtarma, 49.4430 °N 85.0736°E, rocky steppe slopes, 23 June 2020 (fl.), *G.A. Bolbotov et al. s.n.* (ATLB [1100038495]). **Pavlodar region:** Bayanaulsky Gorno-Forest array, Sev. Sklon Lake

Jayasybay, 6 JuLy 1978 (fl. & fr.), *N.T. Lalayan s.n.* (SVER [VER0572129]); Bayanaul Mountain Lesnaya, near the lake, 29 June 1979 (fl.), *N.T. Lalayan s.n.* (SVER [SVER0572130]); Bayanaul Mountain Lesnaya, in the crevices of the slabs on the southern slope, 3 July 1978 (fl. & fr.), *N.T. Lalayan s.n.* (SVER [SVER0572131, SVER0572132]). **Unknown:** 1840 (fl.), *G.S. Karelin & I.P. Kirilov 323* (NW [MW0120062]); [locality illegible], 17 July 1936 (fl.), *R.A. Elenevsky 304* (MW [MW0120061]).

III. NORTH KOREA. Pyongyang: Pyongyang City, 20 June 1959 (fl.), *Pyongyang Bot. Garden s.n.* (PE [01572927]); ibid., 21 July 1959 (fl. & fr.), *Pyongyang Bot. Garden* s.n. (PE [01572924]). **Nampo**: secus vias Chinampo, June 1910 (fl.), *U. Faurie 736* (P [P03872684, P03872666, P03872667]); Chinampo, Sep. 1901 (fl.), *U. Faurie 737* (P [P03872665]); in herbidis Chinampo, Aug. 1906 (fl.), *U. Faurie 754* (E [E00780690]).

IV. SOUTH KOREA. Gyeonggi-do: Gimpo-si, Wolgot-myeon, Munsusan Mt., 17 Aug. 2019 (fl.), *B.M. Nam & S.Y. Yang 190817-001* (ANH, not seen; field photos seen from Nam et al. 2020: Figure 2).

V. MONGOLIA. Arkhangai: 22 June 1979 (fl.), *I.A.Gubanov 341* (MW [MW0187939]); 25 km south from Bat-Tsengel settlement, on dry slopes along the right bank of the Urd-Tamir river, 29 Jul. 1980 (veg.), *I.A. Gubanov 1244* (MW [MW0187987]). **Bayan-Ölgii:** Mongolian Altai, at the river, 49°04′58.2″N, 90°15′42.3″E, 9 Aug. 2008 (fr.), *V.L. Semerikov s.n.* (SVER [SVER0572128]). **Dornod**: Sandy shore of Lake Buir-Nur near the mouth of the Khalkhin-Gol River, 3 km west of the village Buir, 10 Sept. 1980 (fr.), *I.A. Gubanov 5729* (MW [MW0187946]); Eastern aimag Khalkh-Gol., southeast of Lake Buir-Nur, 28 June 1975 (fl.), *O.V. Zhurba 769* (MW [MW0187953]). **Dornogovi [East Gobi]**: 200 km south of Sayn-Shand, Khutag Mts., among the stones, 1250 m, 10 Jul. 1982 (fl.), *I.A. Gubanov 5196* (MW [MW0187980]); Saikhan-Dulan, 20 km south-west of Sain-shanda town, rocky desert, 8 Jul. 1975 (fl.), *O.V. Zhurba 631a* (*C. gobicum*; MW [MW0187935]); 35 km south from Dzun-Bayan. Mount Tahyat-ula. 900 m, down the gorge, 7 July 1982 (fl.), *I.A. Gubanov 4965* (*C. gobicum*; MW [MW0187931]) & *4966* (*C. gobicum*; MW [MW0187930]). **Govisümber**: [locality illegible], 27 June (fl.), *G.N. Ogureeva s.n.* (MW [MW0187991]). **Khentii**: Norovlin, Eren-daba ridge (between Onon and Uldza rivers), 30 km to the northwest from the village Norvlin, on the rocky outcrops among the sospak, 4 Aug. 1985 (fr.), *I.A. Gubanov 10122* (MW [MW0187970, MW0187971]); [locality illegible], 3 July 1985 (fl.), *E.N. Alekseeva IV-26* (MW [MW0187993]); Onon River, near the mouth of the river, 49.06852°N, 111.86137°E, 13 Aug. 2005 (veg.), *A.V. Galanin s.n.* (VBGI [VBGI56095]); Onon River, near the mouth of the river, 48.58832°N, 107.92825°E, 10 Oct. 2005 (fl.), A.V. Galanin s.n. (VBGI [VBGI 56094]). **Khovd**: southern part of the ridge Munkh-Khairkhan, 30 km north of the village Bulgan, deserted slopes in the Ulyastan-Gol valley (the left tributary of the Bulgan), 1450 m, 4 Aug. 1979 (fr.), *I.A. Gubanov 7425 & 7425a* & 7425b (MW [MW0187938, MW0187941, MW0187942, MW0187947]); SWW slope of the northern mountain of the Jergalant-St. 30 km southeast from the southern coast of Khara-Us-Nur. Piedmont rocky desert, 24 July 1979 (fr.), *I.A. Gubanov 6621* (MW [MW0187943, MW0187944]). **Ömnögovi [South Gobi]**: Dzun-Saikhan mountains, commencement of northern trail along the road from Dalan-Dzadagad to pass through Gurban-Saikhan, on rocks in the gorge, 22 Jul. 1943 (fl.), *A. Yunatov 12902* (LE [LE01036905], holotype of *Antitoxicum lanceolatum*); 8 km west of Shuulin border post, Arshantin-nuru mountain range, on slopes, 1 Aug. 1989 (fl.), *I.A. Gubanov & Grubov 243* (*C. gobicum*; MW [MW0187936]); 8 km to the east from the Shuulin border post, in periodically flooded places in saury, 800–880 m, 4 Aug. 1981 (fl.), *I.A. Gubanov 3326* (MW [MW0187986]); Southern spurs of the Western Gurvan-Saihen, 80 km SW from Dalan-Dzadgad, rocky slopes of Bayan, under mountains, 6 Aug. 1981 (veg.), *I.A. Gubanov 3323* (*C. gobicum*; MW[MW0187932, MW0187933]); 8 km east of Shuulin border post, in

intermittently flooded sites, in ravines, 800–880 m, 4 Aug. 1981 (fl.), *I.A. Gubanov 3322* (*C. gobicum*; MW [MW0187934]); 100 km south from Nomgon settlement, 8 km to the east from the Shul border post, in periodically flooded places in saury, 800–880 m, 4 Aug. 1981 (fl.), *I.A. Gubanov 3326* (MW [MW0187986]); [locality illegible], 4 Aug. 1981 (fl.), *I.A. Gubanov 3324* (MW [MW0187985]); eastern spurs of the Gobi Altai Mountains Khurkh-Ul, 110 km from the city of Dalan-Dzadgada, stony gravelly slopes, 28 June 1980 (fl.), *I.A. Gubanov 5949* (MW [MW0187940, MW0187945]); [locality illegible], 30 July 1989 (fl.), *I.A. Gubanov et al. 90* (MW [MW0187973]); against Canyon Bayanzag, 43.57014°N, 104.38321°E, 16 Aug. 2005 (fl.), *A.V. Galanin s.n.* (VBGI [VBGI56099]). **Sükhbaatar**: 30 June 1985 (fl. & fr.), *E.N. Alekseeva 3* (MW [MW0187992]). **Töv:** merger of the Kirkuna and Balgzhi rivers, 47.98992°N, 106.17188° E, 11 Aug. 2005 (fl.), *A.V. Galanin & A.V.Belikovich s.n.* (VBGI [VBGI60202]). **Ulaanbaatar**: Bogd Khan Mountain, 20 km east to southeast of Khan-Bogdo settlement, 1000 m, along ravines, 3 Sept. 1982 (fl.), *I.A. Gubanov s.n.* (*C. gobicum*; MW[MW0187929]). **Uvs**: 10 km east of Ulangom, deserted hillocks near the airfield, 1100 m, 27 Aug. 1979 (fr.), *I.A. Gubanov 8040* (MW [MW0187959]); coast of Baga-Nur (Ubs-Nur Bay on the east side), 15 km southwest of Tes village, steppe on the sands, 780 m, 27 Aug. 1979 (fr.), *I.A. Gubanov 8128* (MW [MW0187989]); circa lacus Ubsa [Uvs Lake], 1879 (fl.), *G.N. Potanin s.n.* (E [E00780576]).

VI. RUSSIA.

VI1. Far East. Amur: Skovorodino District, Ignashino village, slope, 27 June 1982 (fl.), *E. Boyko & V. Starchenko s.n.* (RSA [RSA0286750]); Blagoveshchensk, 1906 (fl.), *F.K. Karo s.n.* (WIS [v0395871]); Svobodnensky District, mouth of the river Guran, coastal cliff, 50.90077°N, 129.00324°E, 127 m, 12 July 1990 (fl.), *V.M. Starchenko s.n.* (ABGI [489]); Svobodnensky District, mouth of the river Guran, 51.40728°N, 127.96577°E, 180 m, 12 July 1990 (fl.), *V.M. Starchenko s.n.* (ABGI [492]); Skovorodinsky District, 53.89416°N, 123.98597°E, 401 m, 9 July 1990 (fl.), *V.M. Starchenko s.n.* (ABGI [494]). **Buryatia**: Baikal Reserve, foot of the southern slope of Khamar-Daban, 950 m, 16 Jul. 2015 (fl.), *N.S. Gamova BR_1966* (MW [MW0159556]); River Chikoy, sands, 26 June 2002 (fl. & fr.), *P.V. Kulikov s.n.* (SVER [SVER0542755]); Selenginskii District, 15 km west to the left bank of the River Selenges, 51.02349°N, 106.47268°E, 4 July 2007 (fl.), *L.M. Dolgaleva et al. s.n.* (VBGI [VBGI56086]). **Zabaykalsky**: wüste Orte um Nerczynsk, July-Aug. 1888 (fl. & young fr.), in Herb. J. Freyn, *K.F. Karo 127* (BRNM [15481/36], holotype of *Vincetoxicum thesioides*); wüste Orte um Nerczynsk, July-Aug. 1888 (fl.), *K.F. Karo s.n.* (PR [PR793598, PR 793599], PRC [PRC455105], possible isotypes of *Vincetoxicum thesioides*); Nertschinsk, auf Sandhügeln, 1889 (fl.), *F. Karo 145* (B [B101097078], L [L.2723144], K [K000872723], Z [Z-000001810]); Nerczynsk, Steisosge souccige Bargathaupe, July 1890 (fl. & fr.), *F.K. Karo 359* (B [B101129282, B101129283], E [E00780569], P [P03872534, P03872697, P03872701], WIS [v0395874]); Nertschinsk, auf sandigen Feldern, 1892 (fl.), *F.K. Karo 145* (E, not see, photo B [B101097079, B101097080]; P [P03872693, P03872711], WA [WA0000027747]); in apricis et rupestribus transbai calensibus [in sunny and rocky hills], 1829 (fl. & fr.), *Turczaninov s.n.* (P [P05341437]); Transbaicalia, provincia Tschita (Czita [Chita]), prope oppid. Borzia, loco glareoro, 5-6 Aug. 1988 (fl.), *A.K. Skvortsov s.n.* (RSA [RSA0286739]); "Mountain Steppe" Reserve, 49.43565°N, 112.14331°E, 7 Aug. 2005 (fr.), *E.N. Roenko s.n.* (VBGI [VBGI 56107]); Kyra village, Kyra River, 49.57207°N, 111.98368°E, 20 June 2010 (fl.), *E.N. Roenko s.n.* (VBGI [VBGI56109]); Ibid., 49.57052°N, 111.97098°E, 20 June 2010 (fl.), *E.N. Roenko s.n.* (VBGI [VBGI 56100]); 15 km south of Starotsurhaytuy, Argun River, 50.15569°N, 119.29833°E, 25 July 2005 (fr.), *A.V. Galanin s.n.* (VBGI [VBGI56110]); Ibid., 50.14073°N, 119.29421°E, 25 July 2005 (fl.), *A.V. Galanin s.n.* (VBGI [VBGI56112]); 49.76137°N, 111.84713°E, cool slope with rocks by the river, 12 July 2006 (fl.), *A.V. Galanin & A.V. Belikovich s.n.* (VBGI [VBGI56120]); S. Kuta, 5 km

below the Argun River, 50.06204°, 119.17478°E, 25 July 2005 (fr.), *A.V.Galanin s.n.* (VBGI [VBGI56114, VBGI56115]); "Agin steppe" Reserve, coast of the Lake Ioji, 50.84623°N, 114.83914°E, 670 m, 31 July 2008 (fl.), *E.N.Roenko & Kozyr s.n.* (VBGI [VBGI56116]); Ibid., 50.86356°N, 114.82004°E, 670 m, 31 July 2008 (fl.), *E.N. Roenko & I.V. Kozyr s.n.* (VBGI [VBGI 56104]); Menzhiken River, 52.06466°N, 113.35580°E, 12 July 2006 (fl.), *A.V. Galanin & A.V.Belikovich s.n.* (VBGI [VBGI60199]); Lower Tsasuch, left bank of Onon River near the bridge Ilmovniki, 50.52110°E, 115.13120°E, 22 July 2005 (fl. & fr.), *A.V. Galanin s.n.* (VBGI [VBGI60205]); Kulusutay, 50.23290°N, 115.68041°E, 7 Aug. 2005 (fr.), *E.N. Roenko s.n.* (VBGI [VBGI 56108]). **Unknown:** Daourie [Transbaikal], *Herb. Maire s.n.* (P [P03872672 right part]).

VI2. Siberia. Altai Republic: mouth of Chuya River, 1st dry, sandy steep mountains, 21 Aug. 1978 (fr.), 650 m, *T.S. Elias et al. 4394* (NY [04171670], PE [01457015], RSA [RSA0286746]); Gorno-Altai a.o. Ongudaysky district, the mouth of the river B. Yaloman, Fine-grained Yu slope], 28 June 1988 (fl.), *S.A. Kurbatsky et al. s.n.* (MW [MW0120074]); frequens in deserto soyng-kirgh. (K) ad Katunjaes & fl. Tschulyschman [Chulyshman River], May–July 1820 (fl.), *Bunge 324* (P [P03872675]). **Altai Krai**: Turksib, 5 km south of Barnaul, along the railway canvases, 14 Jul. 1981 (fr.), *M.S. Ignatov & Ignatova s.n.* (MW [MW0120064]); Klyuchevskiy district. okr. from. Novovoznesenka [c. 52.1933°N, 79.1953°E], typical steppe, , 16 Aug. 1945 (fl. & fr.), *E. Vandakurova s.n.* (MW [MW0120065]). **Irkutsk**: Lake Baikal Region, 158 km NE of mouth of Angara River, an arid zone along Lake Baikal at Bukhta Aya (Aya Bay), 52°47′N, 106°37′E, 460–650 m, desert steppe with loose coarse sandy to gravelly bare “soil”, rarely with open grass cover, 30 June 1979 (fl.), *H.H. Iltis et al. 252* (NY [04171682], WIS [v0383167]); South Baikal region, Olkhon district, Cape Zama [Zhima?], on a steppe sandy slope, 12 Aug. 1977 (fl.), *L. & Z. Malyshevy 1018* (WIS [v0383168]); Distr. Balagansk, in vicinia pag. Ustj-Ossinskaja in declivibus aridis, 29 June–19 Aug. 1909 (fl.), *N. Maltzev 3761* (E [E00780573], MW [MW0120059]). **Khakassia:** dry steep slope of cuesta along Abakan River between Abakan and Askiz, 400 m, 23 Jul. 1987 (fl. & fr.), *D. Murray et al.537* (RSA [RSA0286748]); Abakan, 2 July 1971 (fl.), *A.H. Berkutenko s.n.* (SVER [SVER0572133]); Askiz district, 53°17.923′N, 90°39.548′E, 6 June 2017 (fl.), *M.S. Knyazev s.n.* (SVEG [SVER0926861]); meadow steppes near the railway Art. Tigei (north of Abakan), 11 July 2019 (fl.), *M.S. Knyazev s.n.* (SVEG [SVER0933484]). **Kemerovo:** Tisulsky district, on the way from southern slope of Bolshoy Berchikul to Lake Berchikul, 55°38′00.1″ N, 88°19′30.1″ E, 377 m, 25 June 2015 (fl.), *S.A. Sheremetova s.n.* (KUZ [KUZ008332]); Prokopyevskiy district, 1 km to the west of the village of Northern Maganak (mountain Karaul), foot of the mountain, 7 Aug. 2006 (fr.), *S.A. Sheremetova & T.E. Buko s.n.* (KUZ [KUZ008335]); Chebulinsky district, ca. 55.8952°N, 87.9773°E, 3 July 2001 (fl.), *O.E. Kravets & M.S. Evdokimova KEM 00529* (KUZ [KUZ008331]); Belovsky district, Mount Krutaya, 54.158°N, 86.3143°E, 22 July 2006 (fl.), *A.L. Ebel et al. KEM18150* (KUZ [KUZ008333]); Prokopyevsky district, 1 km west of the village of North Maganak (Mount Karaul), 53.9264°N, 86.773°E, 7 Aug. 2006 (fr.), *S.A. Sheremetova & I.E. Buko KEM00526* (KUZ [KUZ008334]). **Krasnoyarsk**: surrounding the village Sorokino, Mount Turan, stony steppe, 12 June 1965 (fl.), *A. Kuminova & I. Neyfeld s.n.* (MW [MW0120079]); Minusinsky district, okr. village Lugavskoe [c. 53.5206°N, 91.794°E], 2 July 1964 (fl.), *G. Zvereva & N. Drobyshevskaya s.n.* (MW [MW0120083]); dry S slope along the Zakachinskaya channel, 1 June 1910 (fl.), *Anonymous s.n.* (P [P03872501]). **Tomsk**: Region de Tomsk, Jul. 1910 (fl.), *Fedjensko s.n.* (L [L.2723150]); Garchouebeya, near Sorchoe y Ochr Bropbebcroe–Loecoburoba, 23 June 1913 (fl. & fr.), *N.I. Kuznetsov 1428* (WIS [v0395873]). **Tuva**: Tandinsky district, bitter-salty lake Dus-Khol (Svatikovo), 40 km south of Kyzyl, 51°21′N, 94°25′E, 712 m, sandy semidesert, 6 Jul. 2003 (fl. & fr.), *V.V. Nikitin et al. 1268(2)* (PE [01917085]); Ovyursky

district, 20 km from the lake Ubsu-Nur, ok. Ak-chira settlement, snake-vananophyte steppe, 2 Aug. 1973 (fr.), *S. Timokhina & L. Danilyuk 1202* (MW [MW0120078]); Tannu-Tuva, 22 July 1934 (fr.), *B.G. Varvarin s.n.* (MW [MW0120067]); West Sayan Mountains, ca. 40 km S of Kazil, sandy soils and sand dunes near salt lakes Bus-Khol and Svatikovo, 20 July 1998 (fr.), *T. Elias et al. 7898* (RSA [RSA0286747]). **Unknown:** Siberia, [*Gmelin*] *s.n.*, Herb. Linn. No. 310.35 (LINN, lectotype of *Asclepias sibirica*); from Irtysch to Davuria, in sandy soil, *Anonymous s.n.* (M [M0175061]); Dahuria, no date (fl.), ex Herb. Petrop., *Fischer s.n.* (L [L.2723146]); [locality illegible], 20 July 1904 (fl.), *K.F. Karo 170* (MW [MW0120033]); "H. Vuisob.", *Anonymous s.n.* (MO [2365886]); "In lapidufis ad Selengirfir et cels", 1829 (fl. & fr.), *Turczinov s.n.* (E [E00780571]); *Anonymous s.n.* (P [P03872671]); Sib[eria], Herb. de Schihatches, *C.A. Meyer s.n.* (P [P03872676 lower right]); Sibiria, *P.S. Pallas s.n.* (M [M0175062]); Siberia, no date (fl.), *Hohenacker s.n.* (WAG [WAG.1662894]); Siberia, no date (fl.), ex herb. de Franq Leville, *Ledebour s.n.* (P [P03872672 left part]); Siberia, ex Herb. Maire, *Scht. s.n.* (P [P03872676] upper four branches); "Flora Altaica", *Bunge s.n.* (P [P03872674 right part]); "Habitat in Sibiria" , *Anonymous s.n.* (B [B-W 05283 -01 0, B -W 05283 -02 0]); "Habitat in Sibiria; in praealtis Obi ripis", *Anonymous 70* (B [B -W 05283 -03 0]).

VII. Unknown. Ex herbario Linnei, no date (veg.), *Anonymous s.n.* (LD [1743343], possibly an original material of *Asclepias sibirica*); Ex herb. Siegesbeckia, no date (fl.), *Anonymous s.n.* (B [B101006546]).
