# Supplementary figures and images for: ﻿A taxonomic revision of Cynanchumthesioides (Apocynaceae) with two new synonyms
Source: PhytoKeys. 2023 Jan 19;219:11–25. doi: 10.3897/phytokeys.219.93514 (PMC10210048; doi:10.3897/phytokeys.219.93514)

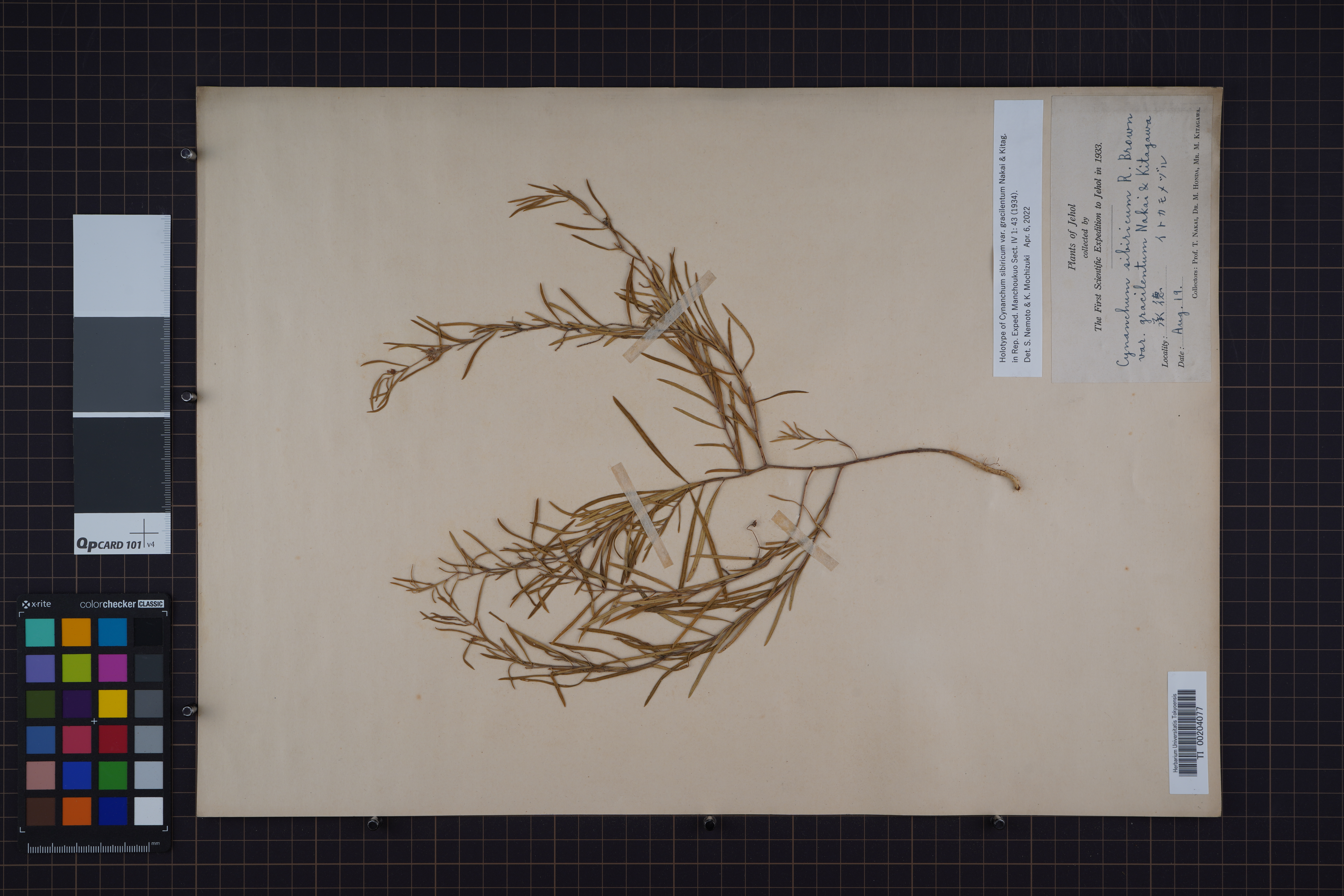

Supplement: Supplementary material 2 — Holotype of Cynanchumsibiricumvar.gracilentum (TI [TI00204077]) [file phytokeys-219-011_article-93514__-s002.jpg]

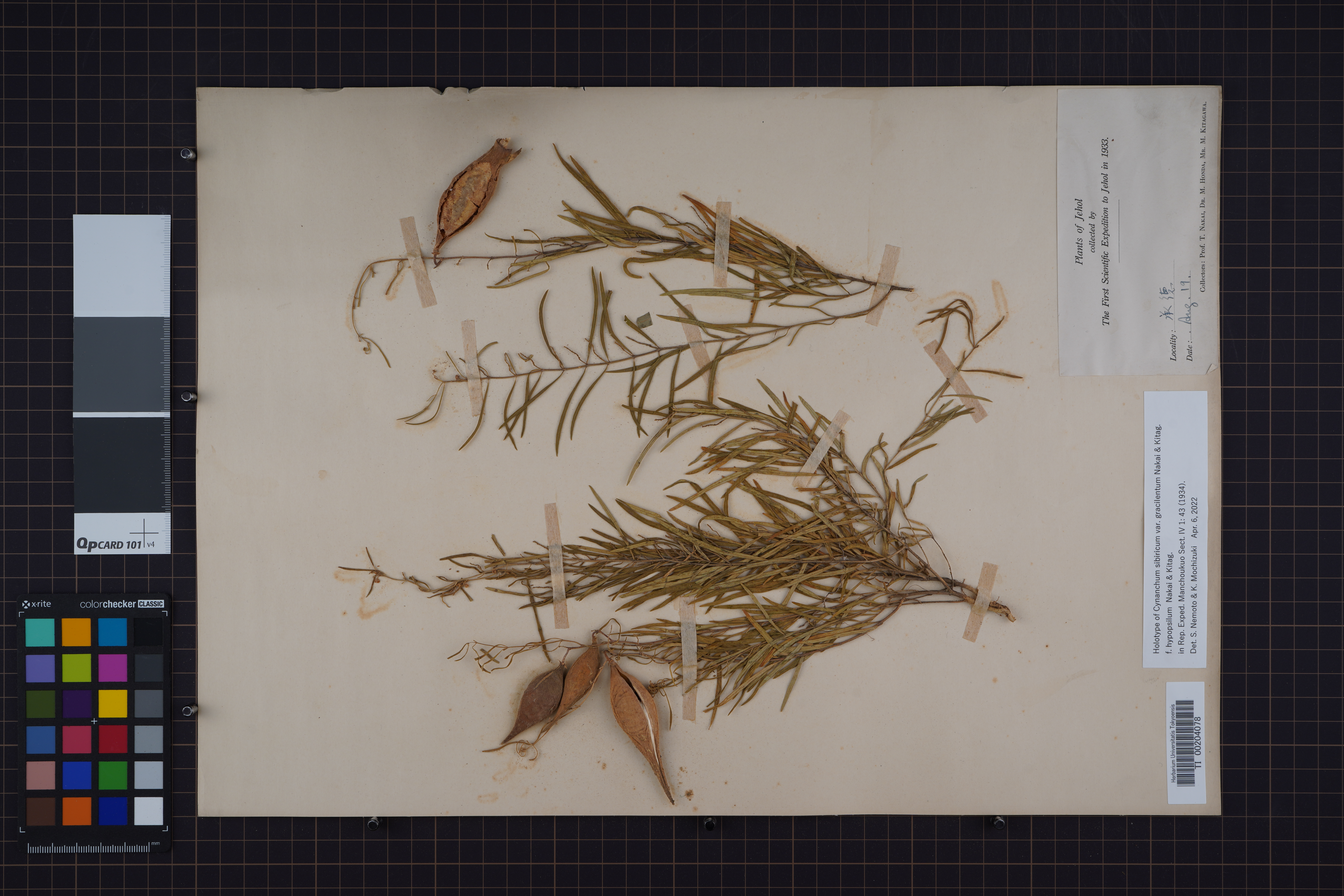

Supplement: Supplementary material 3 — Holotype of Cynanchumsibiricumf.hypopsilum (TI [TI00204078]) [file phytokeys-219-011_article-93514__-s003.jpg]

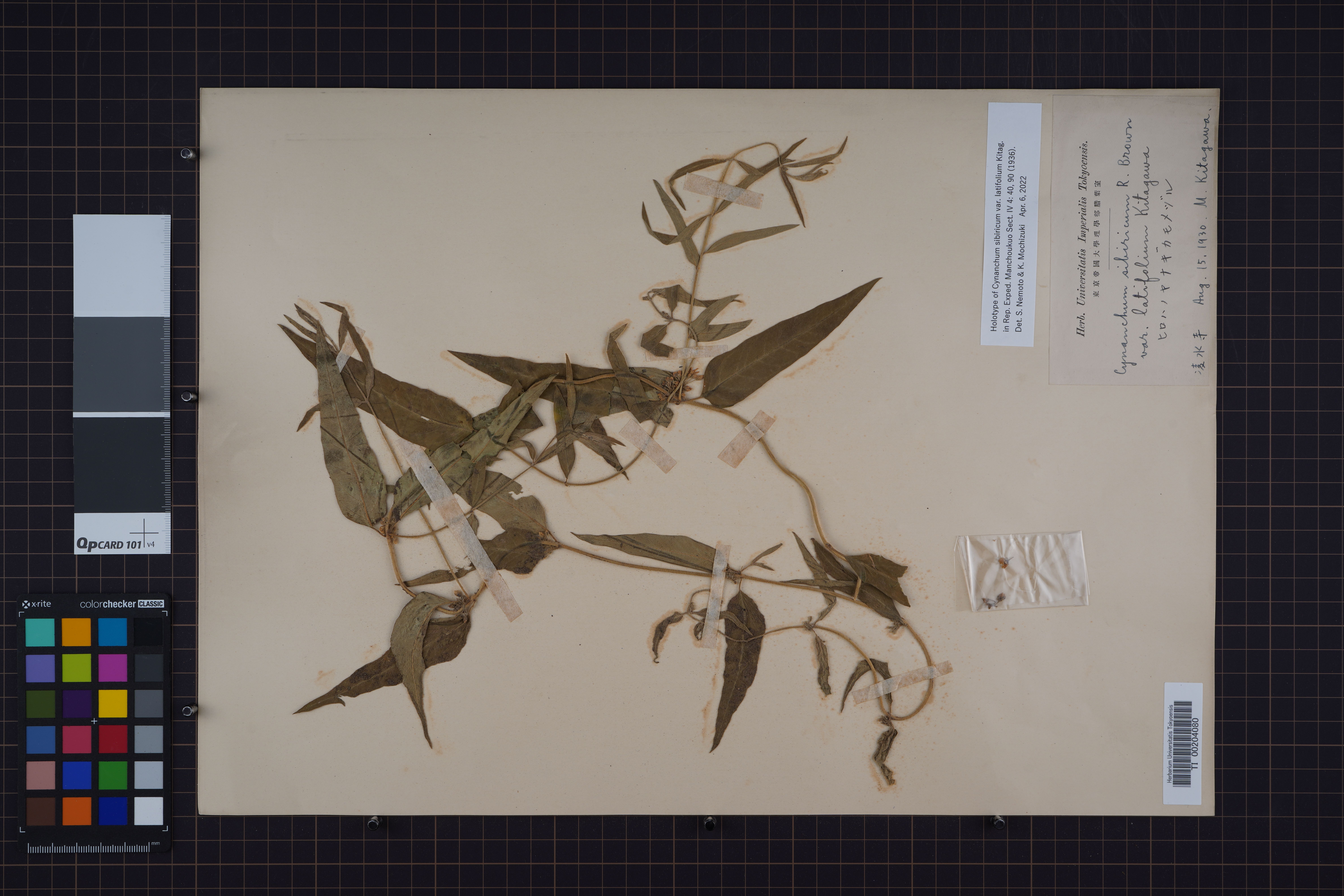

Supplement: Supplementary material 4 — Holotype of Cynanchumsibiricumvar.latifolium (TI [TI00204080]) [file phytokeys-219-011_article-93514__-s004.jpg]
